# Genome-wide definition of selective sweeps reveals molecular evidence of trait-driven domestication among elite goat (*Capra* species) breeds for the production of dairy, cashmere, and meat

**DOI:** 10.1093/gigascience/giy105

**Published:** 2018-08-27

**Authors:** Bao Zhang, Liao Chang, Xianyong Lan, Nadeem Asif, Fanglin Guan, Dongke Fu, Bo Li, Chunxia Yan, Hongbo Zhang, Xiaoyan Zhang, Yongzhen Huang, Hong Chen, Jun Yu, Shengbin Li

**Affiliations:** 1 College of Medicine & Forensic, Health Science Center, Xi'an Jiaotong University, Xi'an, Shaanxi, 710061, People's Republic of China; 2 College of Animal Science and Technology, Northwest A&F University, Shaanxi Key Laboratory of Molecular Biology for Agriculture, Yangling, Shaanxi, 712100, People's Republic of China; 3 Institute of Biochemistry and Biotechnology, University of Veterinary and Animal Sciences, Lahore, 54000, Pakistan; 4 CAS Key Laboratory of Genome Sciences and Information, Beijing Institute of Genomics, University of Chinese Academy of Sciences, Beijing, 100101, People's Republic of China

**Keywords:** goat, resequencing, trait-driven domestication

## Abstract

**Background:**

The domestication of wild goats and subsequent intensive trait-driven crossing, inbreeding, and selection have led to dramatic phenotypic purification and intermediate breeds for the high-quality production of dairy, cashmere wool, and meat. Genomic resequencing provides a powerful means for the direct identification of trait-associated sequence variations that underlie molecular mechanisms of domestication.

**Results:**

Here, we report our effort to define such variations based on data from domestic goat breeds (*Capra aegagrus hircus*; five each) selected for dairy, cashmere, and meat production in reference to their wild ancestors, the Sindh ibex (*Capra aegagrus blythi*; two) and the Markhor (*Capra falconeri*; two). Using ∼24 million high-quality single nucleotide polymorphisms (SNPs), ∼1.9 million insertions/deletions, and 2,317 copy number variations, we define SNP-desert-associated genes (SAGs), domestic-associated genes (DAGs), and trait-associated genes (TAGs) and attempt to associate them with quantitative trait loci (QTL), domestication, and agronomic traits. A greater majority of SAGs shared by all domestic breeds are classified into Gene Ontology categories of metabolism and cell cycle. DAGs, together with some SAGs, are most relevant to behavior, immunity, and trait specificity. Whereas, TAGs such as growth differentiation factor 5 and fibroblast growth factor 5 for bone and hair growth, respectively, appear to be directly involved in growth regulation.

**Conclusions:**

When investigating the divergence of *Capra* populations, the sequence variations and candidate function-associated genes we have identified provide valuable molecular markers for trait-driven genetic mapping and breeding.

## Background

As one of the most popular farm mammals, goats (*Capra hircus*, NCBI:txid9925) were domesticated ∼10,000 years ago [1]. In early domestication, crucial factors were selected, including docility toward humans and loss of wild-type behavioral characteristics [2]. Following the initial domestication events for crops in the Fertile Crescent, together with culture diffusion over Europe, Africa, and Asia, animal domestication had spread rapidly as an integral part of the Neolithic Revolution [3]. Once farming developed in the Middle East and Asia in ∼7000 B.C., human settlements became permanent, and domesticated animals ensured a better supply of food and clothing [4]. After a long period of so-called soft selection, the situation changed dramatically around 200 years ago with the emergence of the *breed* concept [3]. Selection increased intensively in local populations, followed by standardization of trait performance, and reproductive breeding among breeds was seriously reduced, leading to fragmentation of the initial gene pools. More recently, selection pressure has increased again via the use of artificial insemination, resulting in a few industrial breeds with high trait performance, low effective population size, and profound phenotypic changes [5], such as the case of trait-driven breeding for dairy, cashmere, and meat [6].

Goats number ∼800 million in population and in ∼560 breeds (12% of the total recorded mammalian breeds). They are one of the most adaptable livestock on all continents [7] and supply milk, meat, and fiber for human consumption, while thriving on meager fodder and in harsh environments [8]. Despite the importance of this species, the study of goat genomes is still in its infancy compared to that of other farm animals [9]. Nevertheless, positional cloning has demonstrated that the polled intersex syndrome is located on 1q43 of the goat genome [10]; transcriptomic studies have paved the way for in-depth genomics, including various trait-relevant tissues, such as mammary glands, skeletal muscle, and hair follicles. Some genetic studies have also been performed on traits and disease resistance [11]. Although genome-wide studies of goat quantitative trait loci (QTL) and genome sequences have advanced the field [12, 13], it is still lagging behind those of other domestic animals, such as cattle, pig, dog, and chicken.

Our experimental design involves the resequencing (∼29 × in sequencing depth and 99% in genome coverage) of 15 domestic goats representing 3 breeds and 4 wild goats from 2 distinct species. The high-quality sequence data allow us to use high-quality genetic markers (single-nucleotide polymorphism [SNP]; insertion/deletion [indel]; and copy number variation [CNV]) to define artificial selection–related genes in the history of goat domestication. In particular, studies of trait-associated genes (TAGs) provide candidate loci for marker-assisted breeding of domestic goats.

## Results and Discussion

### Sequence variation identified in five goat groups

We sequenced three elite domestic goat breeds (five each), including dairy (Saanen), cashmere (Liaoning cashmere), and meat (Leizhou), and two wild goat species (Sindh ibex, *Capra aegagrus blythi* and Markhor, *Capra falconeri*; two each) as controls (Supplementary Fig. S1). Both the Sindh ibex and Markhor are Pakistan wild goats; the latter categorized as endangered on the International Union for Conservation of Nature Red List (Supplementary Fig. S2). We generated 1,346 Gb (28.8 ×) and 379 Gb (28.6 ×) of raw data for the domestic and wild goats, respectively (Table 1; Supplementary Table S1 and Supplementary Fig. S3). Referenced to the *Capra hircus* genome (GenBank Accession: GCA_003 17765.1), we identified 23,924,294 SNPs,  1,899,827 indels, and 2,317 CNVs.

**Table 1:** Summary of sequencing and variation for domestic and wild goats

Group	N	Raw data (Gb)	Average Uniquely mapped bases (Gb)	Mapping rate	Mean depth	Total SNP (x10^6^)	Total SNP NS/S	no. (x10^6^)	CNV no.	CNV length (Mb)
Domestic	15	89.75	63.43	70.67	28.84	19.04	0.86	1.54	2,028	32.0
Dairy_SN	5	89.23	67.79	75.97	29.11	11.38	0.82	1.02	1,161	20.5
Cashmere_LN	5	88.46	67.33	76.11	28.46	12.33	0.83	1.02	1,096	18.5
Meat_LZ	5	91.56	68.36	74.66	28.96	9.19	0.85	0.83	1,725	21.8

Wild	4	94.72	67.29	71.04	28.64	11.66	0.97	0.99	1,616	19.2
Markhor	2	86.27	62.71	72.69	26.89	4.42	0.87	0.64	1,220	15.4
Sindh ibex	2	103.16	71.87	69.66	30.39	7.63	1.06	0.73	1,352	15.3
Total	19	90.80	64.24	70.75	28.80	23.92	0.95	1.90	2,317	35.5

Note: Locations where goat breeds are farmed are labeled, Saanen or SN, Liaoning or LN, and Leizhou or LZ. Ratios of synonymous and nonsynonymous SNPs are listed under NS/S.

#### Single-nucleotide polymorphisms

We analyzed the high-quality SNPs with a criterion of a minimum depth ≥8 in every individual sample (Supplementary Fig. S4). In addition, we validated the SNP calling accuracy rate (97.43%) using a sequence-capture next-generation sequencing (NGS)-based genotyping method (Genesky Biotechnologies, Shanghai, China; Supplementary Note).

First, the SNPs were partitioned into intergenic (76.20%), intronic (23.06%), and protein-coding (0.74%) SNPs, and subsequently, the ratio of nonsynonymous to synonymous substitutions (NS/S) was calculated as 0.95 on average. However, the NS/S ratio shows variable distributions when correlated to minor allele frequency (MAF) in the low SNP rate region or the SNP desert (Supplementary Table S2; Supplementary Figs. S5 and S6). Second, we identified millions of SNPs within and between the wild and domestic goat groups. Although the number of wild goat–specific SNPs is smaller than that of the domestic group ( 5,598,396 vs.   12,434,312 and  6,061,698 shared), this result may reflect biased sampling (15 vs. 4) rather than true genetic heterogeneity in the groups. Third, among the SNPs unique to each domestic breed, the dairy breed appears to have slightly more unique SNPs, indicating the recent introduction of genetic heterogeneity [14, 15], as opposed to the cashmere breed, which appears to have more in total when breed-shared SNPs are considered (Supplementary Fig. S7). At low MAFs, there is a higher proportion of breed-specific SNPs than the total, but there is a transition at MAF 20%, where the breed-specific SNP proportion becomes obviously less than the total (Supplementary Fig. S8). In addition, the meat breed has more ancient SNPs with higher MAFs than the other two breeds, whereas the cashmere breed is relatively young or less selected as it has more low-frequency SNPs (Supplementary Fig. S8). Fourth, we compared heterozygous SNPs across all chromosomes and found that the meat breed has significantly lower heterozygosity (*P* = 0.0022) than the two other breeds, suggesting that there may be strong or long-term selection during its breeding (Supplementary Table S3).

#### Insertions/deletions

We categorized  1,899,827 indels with nearly equal numbers of insertions and deletions, of which ∼0.13% (2,420) were found in protein-coding sequences and partitioned into 32.72% (792) in-frame (3-bp indels) and 1,628 out-of-frame indels that lead to an average of 499 pseudogenes per individual sample. Similar to the trend observed for SNPs, there are much rarer indels in the total (Supplementary Figs. S9 and S10); we observed more indels that were domestication specific than wild specific ( 924,352 vs.  363,589) and more indels in the dairy and cashmere breeds compared to the meat breed (Supplementary Fig. S11; Supplementary Table S4).

In addition, based on our indel data, *AADAC* (arylacetamide deacetylase) appears to be selected in the dairy breeds, encoding an enzyme responsible for the hydrolysis of drugs [16]. The *MYT1L* (myelin transcription factor 1-like) marker associated with syndromic intellectual disability and early-onset obesity has shown a meat-specific high-frequency indel frame-shift polymorphism [17] (Supplementary Table S5).

#### Copy number variation

In the 1-kb window, there are 2,028 (246 genes; spanning a 32.0-Mb genomic region) and 1,616 CNVs (144 genes; spanning a 19.2-Mb genomic region) in the domestic and wild goats, respectively. The wild goats and the meat breeds have relatively higher numbers of CNVs than their domestic counterparts and two other domestic breeds (Supplementary Table S6). The meat goats have more breed-specific CNVs compared to dairy and cashmere breeds (38 vs. 33 and 22); 11 CNVs were shared by all three breeds (Supplementary Fig. S12).

Interestingly, consistent with a previous study [18], we observed high-frequency domestication-specific CNVs in the region including *ASIP* (agouti signaling protein) and *AHCY* (adenosylhomocysteinase) genes, which are related to skin pigmentation and coat color in sheep [19]. Goats with white hairs (Saanen and Liaoning cashmere goats) have many more copies of *ASIP* and *AHCY* than those goats with colored hairs (Leizhou and wild goats; Supplementary Table S7). This result was also confirmed in our larger population sampling with a white and black coat population (n = 54; Supplementary Fig. S13). Thus, the*ASIP-AHCY* region is a domestication locus in goat and may be select for coat color.

Finally, to validate whether CNV loci were associated with dairy traits, 12 candidate dairy-specific CNV loci were detected in 130 Guanzhong dairy goats using the AccuCopy assay (Genesky Biotechnologies, Shanghai, China). Our CNV association study points to two CNV loci (including *APOL3* and *NEM6*; *P* < 0.01) for dairy and growth traits (Supplementary Table S8). *APOL3* (apolipoprotein L3) is a lipid-transport and metabolism-associated gene that is also highly duplicated in beef breeds [20], and *NME6* (NME/NM23 nucleoside diphosphate kinase 6) is suggested to play a role in cell growth and the cell cycle [21]. This study indicates that duplication of the *APOL3* locus conferred a selective advantage to dairy, while there is a negative correlation between the duplication of *NME6* and growth, and need further investigation in dairy goat.

### Population structures of domestic and wild goats

**Figure 1:**
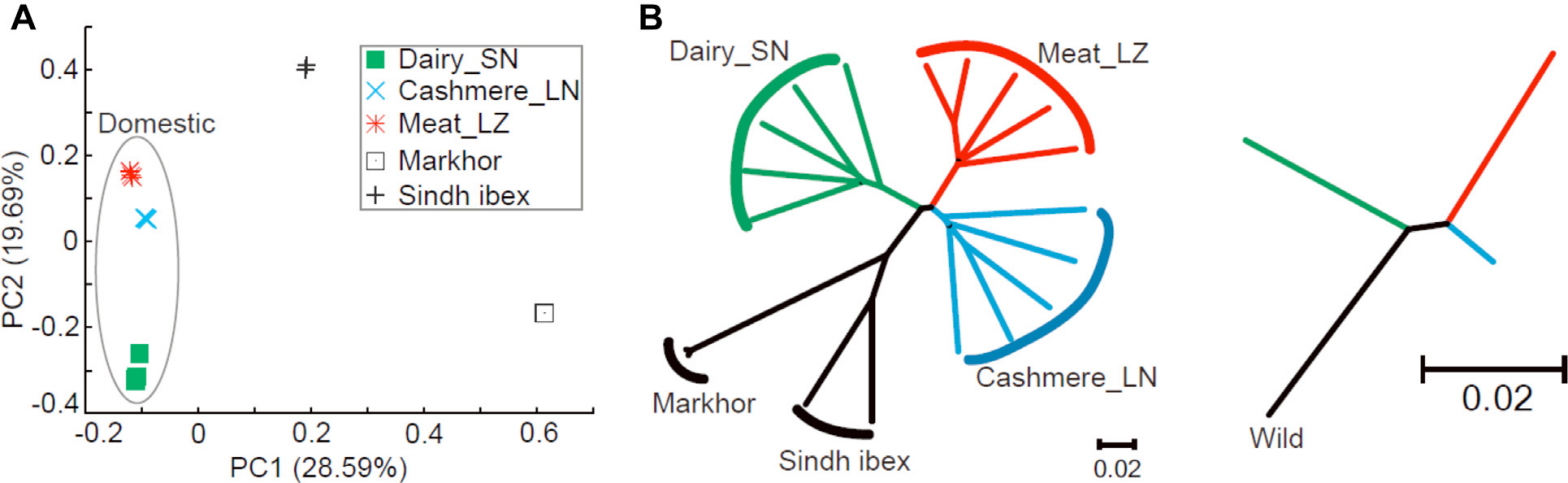
Phylogeny and population structure of goats. **(A)** PCA based on all identified autosomal SNPs. **(B)** Neighbor-joining tree based on autosomal SNPs. SN: Saanen dairy goats, LN: Liaoning cashmere goats, and LZ: Leizhou goats. Markhor and Sindh ibex are wild goat ancestors.

We used principal component analysis (PCA) and phylogeny reconstruction to evaluate the population structure of the domestic breeds. First, based on our genome-wide SNP data, we found that Sindh ibex is genetically closer to the domestic breeds compared to Markhor, which is consistent with previous reports [22]. Second, our PCA result suggests that the domestic breeds and the wild breeds are both distant and distinct (Fig. 1A), where, as the neighbor-joining tree shows, the two Chinese domestic breeds are closer to each other and the Saanen breed is closer to the wild goats than the other two domestic breeds. Third, all results collectively suggest that the domestication traits for dairy production may occur ahead of cashmere and meat in goat domestication [15] (Fig. 1).

### SNP desert–associated genes

**Figure 2:**
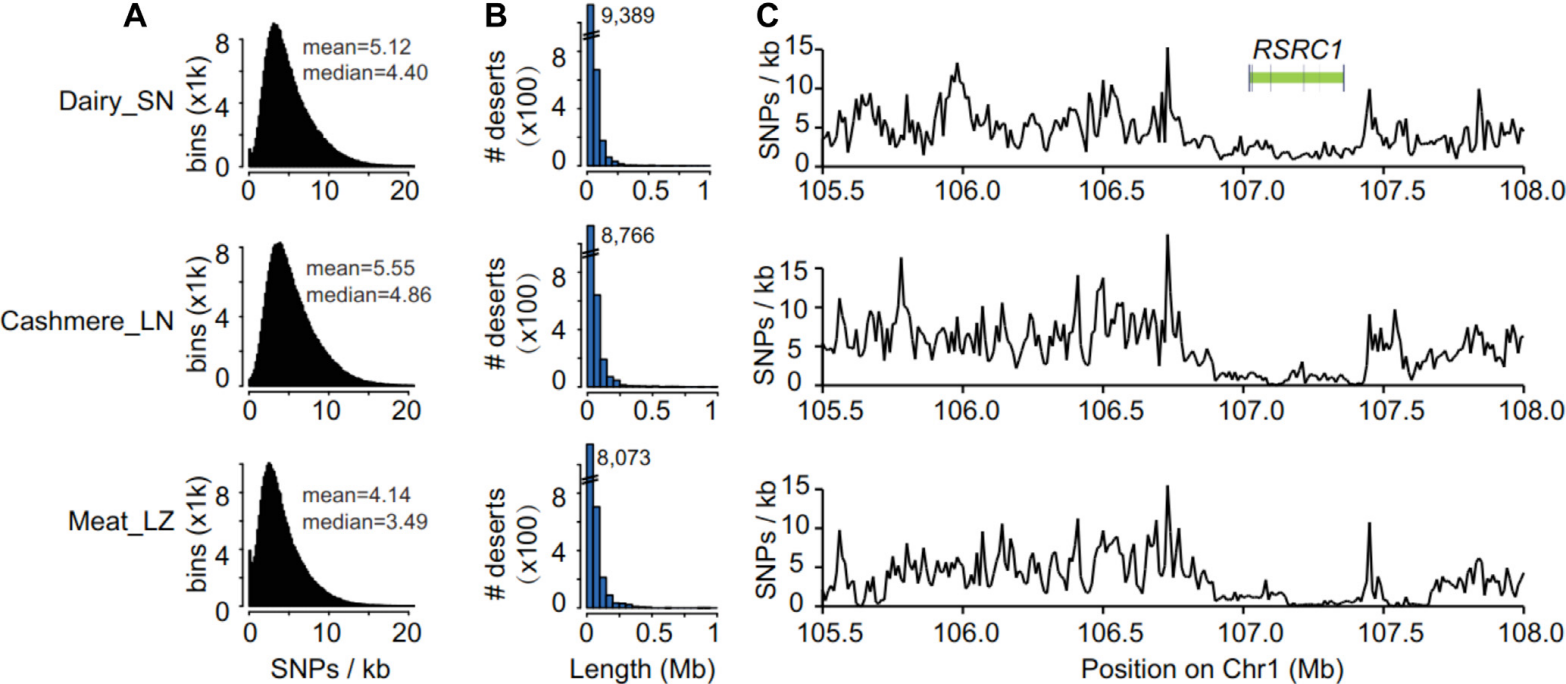
SNP deserts of three domesticated breeds. **(A)** SNP rate distribution. Mean and median SNP rates are labeled by peaks of distributions. **(B)** SNP desert length distribution. **(C)***RSRC1*(arginine and serine rich coiled-coil 1) in a SNP desert region shared by all three breeds. SN: Saanen dairy goats, LN: Liaoning cashmere goats, and LZ: Leizhou goats.

SNP deserts are often linked to beneficial mutations as selective sweeps that are subjected to strong purifying selection [23]. The SNP deserts are defined as genomic regions with the lowest 10% SNP rates (10-kb windows). SNP desert-associated genes (SAGs) are selected if they are harbored by SNP deserts (>30%; Fig. 2). We noticed that there is a bimodal SNP rate-only distribution in the dairy and meat breeds; the large absence of SNP-poor regions suggests the effect of both stronger recent purifying selection and a lack of recent introduction of genetic heterogeneity in the cashmere breed compared to the two domestic breeds. In addition, the lower mean and median SNP rates of the meat breed (Fig. 2A; Supplementary Fig. S14) suggest overall poorer genetic heterogeneity or heavier inbreeding. In total, 277.39-Mb (3,950 SAGs), 278.33-Mb (4,395 SAGs), and 273-Mb (3,447 SAGs) SNP deserts were detected for the dairy, cashmere, and meat goat genomes, respectively (Fig. 2B; Supplementary Table S9). For the 1,196 SAGs shared among the domestic breeds, Gene Ontology (GO) enrichment shows only two major categories: metabolism and cell cycle regulation (Supplementary Fig. S15a; Supplementary Table S10).

To further investigate, we also examined the large SNP deserts (>100 kb in length) as well as the top 10 larger deserts unique to each domestic breed. For the SNP deserts >100 kb in length, it is consistent that the dairy (1,112) and cashmere (1,503) breeds had more SAGs than the meat breed (1,044); the function of the 231 breed-shared SAGs appears to be related to signal transduction (such as *RSRC1*; Fig. 2C and Supplementary Figs. S15b and S16). To provide alternative insights, we scrutinized the top 10 SNP deserts in three breeds. When looking for breed-shared SAGs in the top 10, we observed only one SNP desert, including *AR* (androgen receptor) gene on chr X. *AR* is a hormone-inducible DNA-binding transcription factor that plays an essential role in male reproduction; its knock-out male mice display severely impaired reproductive tracts and sexual behavior [24], which indicate male reproduction may have been an important evolutionary force during goat domestication. For the top 10 SNP deserts found in each breed, the meat breed has two unique loci, and the dairy breeds have four, but none for the cashmere breed (Supplementary Discussion, Supplementary Table S11–S13).

### Domestication-associated genes

**Figure 3:**
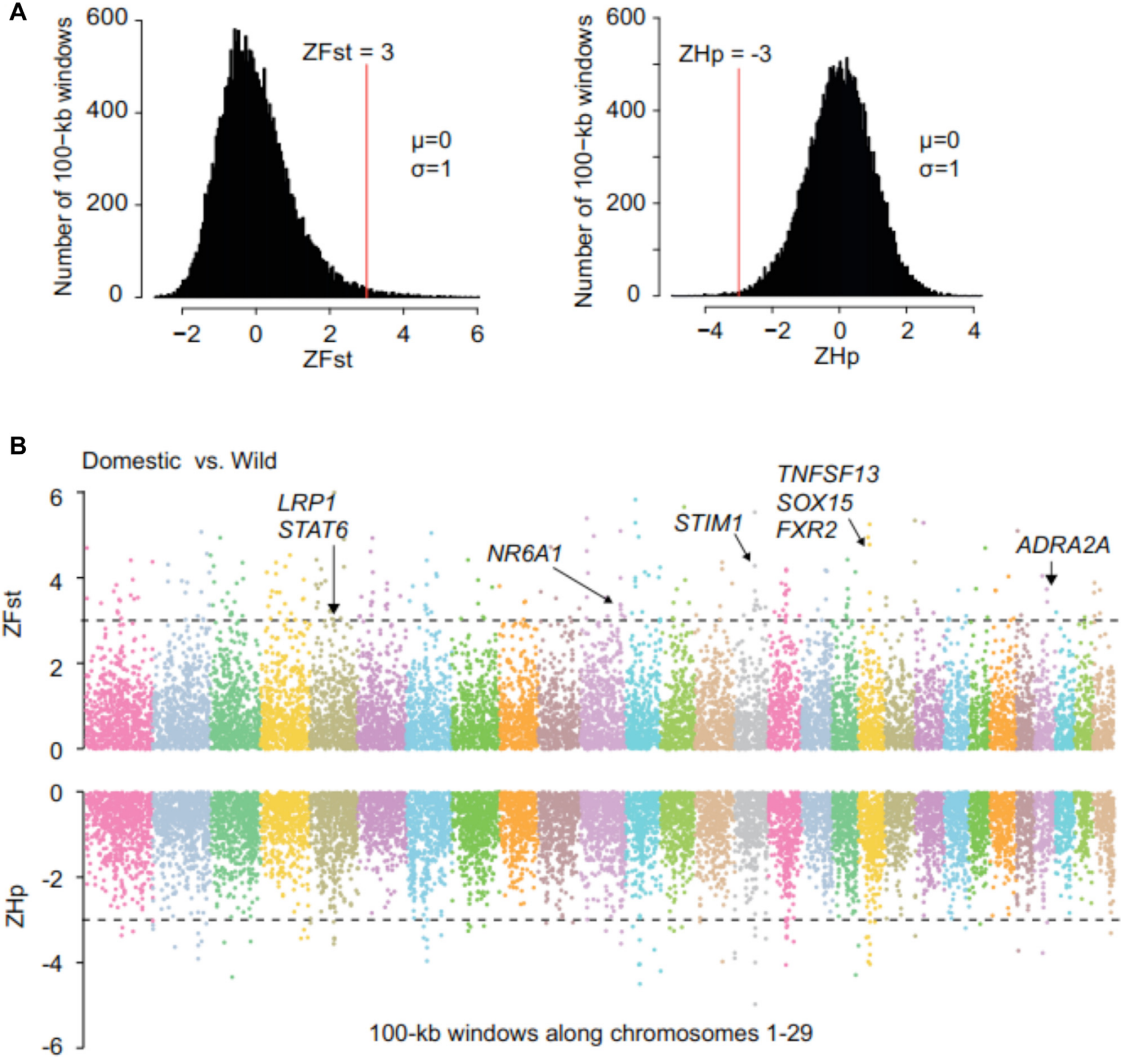
Candidate regions for DAGs. **(A)** Distribution of Z-transformed pooled heterozygosity (ZHp) in 15 domestic goats, and Z-transformed fixation index (ZFst) between wild and domestic goats for autosomes 1 to 29. Red vertical lines indicate thresholds. **(B)** Positive end of ZFst distribution (ZFst >3) and negative end of ZHp distribution (ZHp<-3) used for extracting outliers. Dashed lines indicate cutoff values. DAGs labeled are discussed in the text.

To detect the sequence signature of selective sweeps over large genomic regions, we first calculated the pooled heterozygosity (Hp) using autosomal SNPs from all individuals of the domestic breeds in a 100-kb sliding window. We also calculated the fixation index (Fst), which indicates population differentiation between domestic and wild populations in a 100-kb sliding window based on autosomal SNPs. We then transformed the Hp and Fst into Z (Hp) and Z (Fst), respectively, and the protocol defined 67 DAGs in a collective genomic length of 3.2 Mb (Fig. 3). The 67 DAGs are all overlapped with SAGs in one, two, or three goat breeds (Supplementary Fig. S17, Supplementary Table S14). Our GO enrichment analysis indicates that the significant categories (false discovery rate (FDR), q <0.001) are negative regulation of gene expression, protein import into the nucleus, and docking (Supplementary Fig. S18, Supplementary Table S15).

This set of DAGs may contribute to behavioral, immune, and morphological differences between domestic and wild goats. First, genes that directly influence the nervous system and behavior include *ADRA2A* (alpha-2-adrenergic receptors, which regulate neurotransmitter release) and *FXR2* (fragile X mental retardation, autosomal homolog 2, which are required for the presence of behavioral circadian rhythms) [25, 26]. Second, *TNFSF13* (tumor necrosis factor (ligand) superfamily, member 13) and *STIM1* (stromal interaction molecule 1) are located in the region associated with cattle body weight gain [27, 28] and regulation of B-cell development and T cell-mediated immune regulation during chronic infection [29, 30], respectively. Third, the morphological difference involves genes: *NR6A1* (nuclear receptor subfamily 6 group A member 1), which affects the number of vertebra, one of the most characteristic morphological changes in domestic pigs [31], and *STAT6* (signal transducer and activator of transcription 6), which is associated with body weight as well as carcass and growth efficiency traits [32] (Supplementary Discussion). These findings based on the analysis of sequence variations support the idea that frequent artificial selection in the processes of domestication lead to preferred behavior; such as docility, improved immunity for infectious diseases, and higher trait-associated quality such as meat and milk production.

### Trait-associated genes of domestic breeds

To uncover genetic variants involved in local adaptation and selection in the three breeds, we performed Fst and cross-population extended haplotype homozygosity (XP-EHH) in a 100-kb window on SNPs from one breed against a pool of the two other breeds. Using the criterion of Fst >4 and a top 1% outlier of XP-EHH, we defined 54 TAGs (Supplementary Table S16).

To further explore artificial selection–related genes in the TAGs, 200 SNP genotype frequencies at nonsynonymous sites within TAGs were detected using 287 individuals representing seven populations in China. First, consistent with 19 sequencing individuals, the *GDF5* (growth differentiation factor 5) T217C (amino acid changed: R73G) locus of meat breeds (Leizhou and Hainan) is dominant with the C allele, and the T allele is dominant in the dairy, cashmere, and wild goats. As R is conserved among all other known mammal sequences except goat, we suggest that T allele is ancestral whereas C is selected. In addition, we looked into the breeding history of Leizhou and learned that the body size is smaller than that of Sannen and Liaoning cashmere goats. *GDF5* is a member of the TGF-beta superfamily, which is involved in height [33] and multiple skeletal structures [34] (Fig. 5, Supplementary Fig. S2). Second, *LRP4* (low-density lipoprotein receptor-related protein 4) is detected with a conserved amino acid change (TT at the 266th nucleotide) in goats, and it also showed opposite selection in the meat and other goat breeds (Supplementary Fig. S19). A functionally related candidate gene that affects bone-mass homeostasis and with a central role for high bone mass is syndactyly, a sclerostin receptor [35].

**Figure 4:**
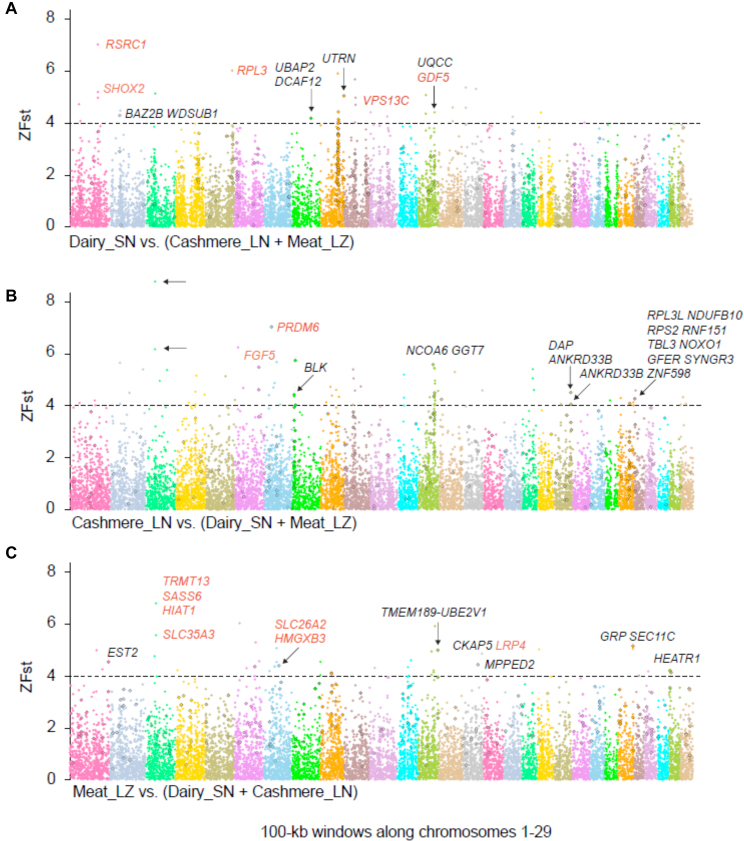
Candidate selective sweep analysis for each economic breed. Selective sweeps and their associated genes are shown in three breeds: **(A)** Saanen, **(B)** Liaoning cashmere, and **(C)** Leizhou. Windows passed the threshold ZFst >4 and the top 1% XP-EHH scores are extracted as selective sweeps. TAGs labeled in color are discussed in the text.

The TAGs are cross-referenced with data on co-localization with cow and sheep QTLs and SAGs (Fig. 4) [36]. A striking correlation was detected between putative selective sweeps and SNP deserts; there are 660, 912, and 1,841 genes shared by SAGs and QTLs unique to the dairy, cashmere, and meat breeds, respectively (Supplementary Fig. S20). Most of these genes were enriched in GO categories of metabolic process, biological regulation, and response to stimulus, whereas the trait-specific categories include reproduction and growth (Supplementary Fig. S21). Therefore, our results illustrate the important role of biological pathways influencing growth in the goat.

**Figure 5:**
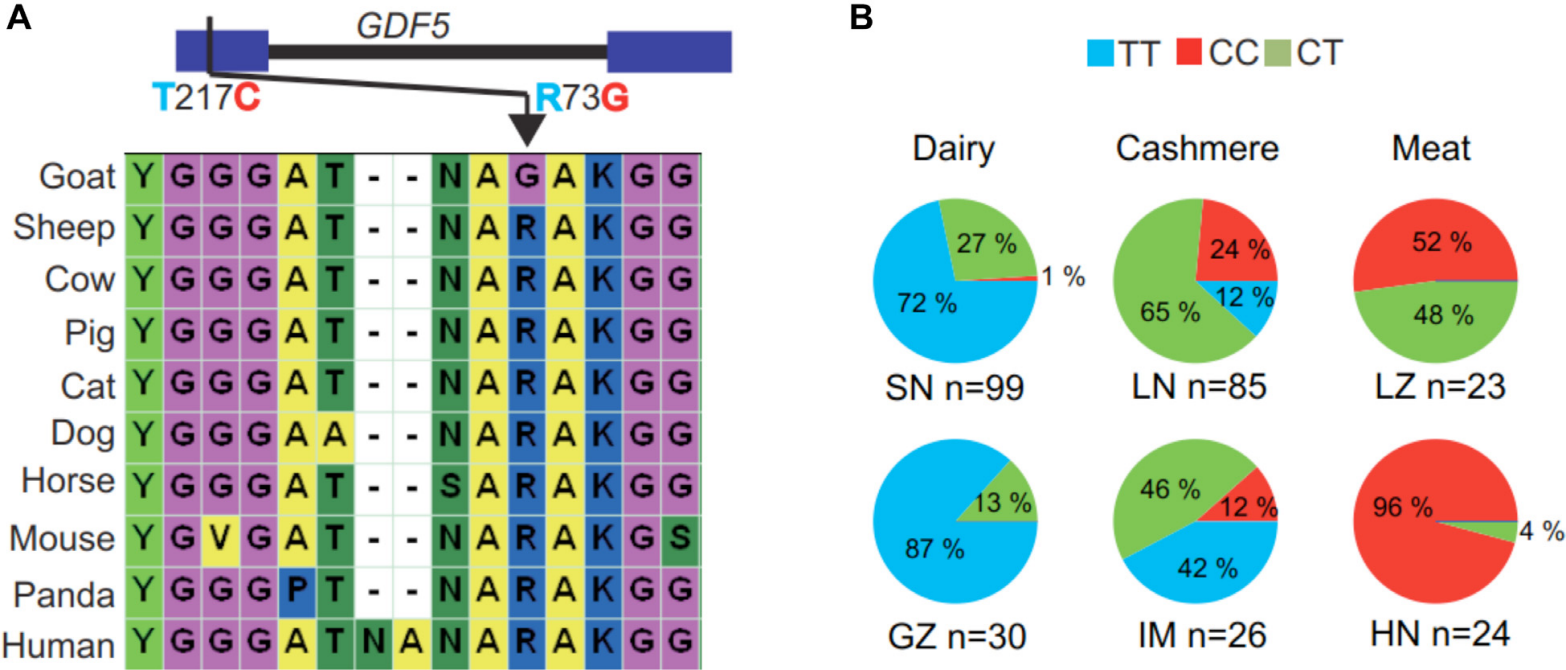
Opposite selection for sites in *GDF5* of dairy and meat breeds. **(A)** Nonsynonymous SNP T217C (R73G) located in the first exon of *GDF5*. Amino acids at this position are highly conserved in other mammals. **(B)** Frequency diverged in different economically relevant traits. Allele T is dominant in the dairy breeds (Saanen and Guanzhong), whereas C is dominant in the meat breeds (Leizhou and Hainan). SN: Saanen goat, LN: Liaoning cashmere goat, LZ: Leizhou goat, GZ: Guanzhong goat, IM: Inner Mongolian cashmere goat, HN: Hainan goat, MA: Markhor, SI: Sindh ibex. Note: Sequencing data are as follows: LZ: CC CC CC CC CC, LN: TC CC CC TC TC, SN: TT TT TT TT TT, MA: TT TT, SI: TT TT.

#### TAGs in the dairy breed

There is no gene shared by TAGs, SAGs, XP-EHH, and QTL (Supplementary Fig. S20a). Many TAGs are associated with milk traits and growth development. Among them, the region including *RSRC1* and its neighbor gene *SHOX2* (short stature homeobox 2) located in chr 1 was under high selection. Polymorphism of *RSRC1* is associated with altered brain function in schizophrenia [37] and height detected in the tails [38]. *SHOX2* is involved in height and chondrogenesis [33, 39] (Fig. 4A). These findings imply that the RSRC1-SHOX2 region may be related to selection for height. Additionally, *RPL3* (ribosomal protein L3) is reported to be highly expressed in the breast milk fat globule, involved in regulation of energy balance, suggesting that translational pressure is at work during lactation [40]. Specifically, *VPS13C* (vacuolar protein sorting 13 homolog C) is suggested to act on glucose homeostasis for high milk production in dairy cows [41], and another member of the same gene family, *VPS13A*, has also been reported in pigs undergoing directional selection for heat adaptation [42]. *VPS13B* was detected within a QTL associated with leg morphology, related with fertility and milk production in cattle and buffalo [43]. We propose that genetic variants within VPS13 family genes may have been selected during farm animal domestication, and this family may play an important role for farm animal production and adaptation.

#### TAGs in the cashmere breed

Among the trait-related regions, there are two neighbor loci located in chr 3 with high powerful selection including no gene. The region on chr 10 with strong support contains *PRDM6* (PR domain containing 6), belonging to the PRDM family of transcriptional repressors, and is reported to be highly expressed in NOTCH1-deficient mice embryos. We expected *PRDM6* to be a candidate gene for the cashmere trait because *NOTCH1* is considered to control follicular proliferation rates and melanocyte populations [44]. Moreover, *FGF5* (fibroblast growth factor 5) stands out because it is an inhibitor of hair elongation and is associated with hair growth and length in mammals [45, 46]. To further annotate the goat *FGF5*, we performed an association analysis between exonic mutations and cashmere-related traits in 224 Inner Mongolian cashmere goats by Sanger sequence. Cashmere production and body weight appear to be associated with one synonymous mutation in exon 3 of *FGF5* (Supplementary Fig. S22). The genes we described could be used as markers for improved cashmere goat production and breeding better cashmere goats, or they may be potential targets for genetic manipulation.

#### TAGs in the meat breed

The most important TAGs to the meat breed are four genes: *HMGXB3, SLC26A2, goat_GLEAN_10 018 710*, and *GOAT_ENSBTAP00000044216* by TAGs, SAGs, XP-EHH, and QTL (Supplementary Fig. S18c). Mutations in the solute carrier family 26 sulfate transporter, member 2 gene (*SLC26A2*) altered residual sulfate transporter activity, associated with short stature and skeletal dysplasias [47]. Among other trait-related genes, the region on chr 3 with the two next highest intensity signals include *SASS6, HIAT1*, and *SLC35A3* genes; the *HIAT1* gene may encode a novel sugar transporter and disruption causes globozoospermia and infertility in mice [48]. The mutations in the *SLC35A3* gene were associated with vertebral and multiple organ malformations in cattle and human [49, 50]. Functional characterization of these genes is likely to provide insights to improve the economic benefit of meat goat, which are appealing candidates for further investigation.

## Conclusion

In this study, we interrogated whole genome sequences from three trait-driven goat breeds and assessed three categories of sequence variation SNPs, indels, and CNVs to search for the functional relevance of three categories of candidate genes SAGs, DAGs, and TAGs. First, we used several methods, including SNP desert, fixation index, pooled heterozygosity, and XP-EHH, to define these candidate genes based on the allelic frequencies of the different sequence variations. Although the sampling itself is rather limited for each breed, a number of follow-up studies with increased population sampling showed consistent results. Second, we grouped the breeds and the data in various ways for detailed analyses, sometimes *casting a larger net* (such as SNP desert and QTL data) and investigating discrete lists in other cases, trying to provide an overview of the genetic landscape of selection-centric genetic heterogeneity in the up-to-date molecular terminology. Third, the candidate genes we described as DAGs and TAGs are complex in function but were clearly biased toward certain functional categories. It is essential to validate them in a specialized breed with a larger population before any mechanistic studies. Finally, NGS technology provides an efficient tool for systematically deciphering the genetic background of domestication and trait selection in a thorough way for goats and other farmed animals. We should heckle GO and expression information at the same time while we are expecting thousands of gene sequences to become available in the years to come.

## Methods

### Sample collection and sequencing

We sequenced DNA samples from 19 goats: 4 from wild female goats (2 Markhor and 2 Sindh ibex) and 15 from domestic male goats (5 Saanen collected in 2008), 5 Liaoning cashmere goats (collected in 2006), and 5 Leizhou goats (collected in 2007). To validate the sequence variation at the population level, we genotyped using sequencing polymerase chain reaction amplicons in 7 domestic breeds, including 99 Saanen, 85 Liaoning cashmere, 23 Leizhou, 16 Dera Din Panah, 30 Guanzhong, 26 Inner Mongolian cashmere, and 24 Hainan goats. The samples from Markhor and Sind ibex were collected from skin biopsies, Quetta, Pakistan. Blood samples from the domestic goats were collected in China. DNA sequences were obtained using paired-end sequencing (2 × 150 bp) technology on the Illumina HiSeq X10 platform. The institutional review board of the Xi'an Jiaotong University Health Science Center approved the study protocol (project identification code 2011–054).

### Processing raw reads

The procedure to remove low-quality reads included meeting one or more of the following criteria: N content more than 10%; >60% read length below Q7; reads overlapping >10 bp with the adapter sequence and a maximum of 2 bp mismatches to the adaptor sequence; paired-end reads overlapped by >10 bp with others; and duplicated reads. We also trimmed up to 10 bp at the 5’ end or 30 bp at the 3’ end of a read if the local N content was >20%.

### Read mapping and quality control

We used BWA 0.5.9 (BWA, RRID:SCR_010910) to map the clean reads onto the reference genome of *Capra hircus* genome V1. The command “aln -t 4 -e 10” was used to find the suffix array coordinates of the good hits of each read. Then, we used the command “sample -a 500” to convert suffix array coordinates into chromosomal coordinates and paired reads. Other parameters were set to the defaults. We filtered the alignments as follows: a mapping quality score lower than 20; nonunique alignments; and duplicated alignments.

### Calling and validation of SNPs, indels, and CNVs

First, SNPs were called at the population scale using ANGSD, with parameters referring to a previous publication [51]. We filtered out the locus with a minimum depth <8 in all individuals and called a heterozygous SNP in one individual only when both alleles were supported by at least four reads. We validated the SNP calling rate (97.43%) using an NGS-based target region genotyping method by Genesky Biotechnologies (Shanghai, China). Second, Dindel v1.01 was used to call short indels (1–5 bp) in each individual [52]. We called an indel only when the non-ref allele was covered by at least two reads on each strand. Then, we filtered out the results that met one or more of the following three criteria: quality reported by Dindel below 20, reference homopolymer length longer than 10 bp, and length of insertion or deletion longer than 5 bp. Third, the Control-FREEC software was used to detect CNV based on pairwise comparisons [53]. With a 1-kb window, we compared the coverage depth between the window and the average depth and identified CNV regions that were different from the reference. We merged the overlapped CNV regions among different samples.

### Population structure analysis

We performed principal component analysis (PCA) with all population-scale autosomal SNPs using the Eigensoft package (Eigensoft, RRID:SCR_004965) [54]. The phylogenetic tree was constructed based on all autosomal SNPs, with the evolutionary distances measured by p-distance with PHYLIP (PHYLIP, RRID:SCR_006244) [55].

### Definition of SNP deserts

Based on the SNP data, we computed the SNP rate in 10-kb sliding windows. We normalized the SNP rates over the length of the ≥ 8-fold aligned sequence in each bin rather than the bin size, and bins with less than 1 kb of the aligned sequence were rejected. We then selected the windows with the lowest 10% SNP rate of the genome data and joined these windows as a longer region if the gap between them was ≤10 kb. We defined these low SNP-rate windows or regions as “SNP deserts.”

### Selection analysis

To find a selective sweep in the domestic lines, Hp and Fst were used to extract outliers [56]. For each 100-kb window, we determined the number of reads corresponding to the most and least abundant SNP alleles (nMAJ and nMIN), Hp = 2∑nMAJ∑nMIN/(∑nMAJ+∑nMIN)^2^. With the same 100-kb window, the Fst was calculated between 15 domestic and 4 wild goats. We then transformed Hp into ZHp: ZHp = (Hp-μHp)/σHp and Fst into ZFst: ZFst = (Fst-μFst)/σFst. For DAG analysis, we applied a threshold of ZHp = -3 OR ZFst = 3 for detecting putative selective sweeps. For TAG analysis, we measured the pairwise Fst and tested one domestic breed and a pool of the other two breeds. The windows pass the threshold of ZFst = 4, and the top 1% XP-EHH [57] scores were extracted as candidate selective sweep regions. Genes residing in these extracted regions were indicated as candidate-selected genes.

### QTL mapping

We downloaded known sheep and cow QTL data from Animal QTLdb [58] and qualified the data by filtering out the terms with “trait association” or with *P* > 0.05. We aligned the genome sequences of sheep and goat with lastz (version 1.02.00) and mapped the QTL to goat chromosomes based on the axt file produced by lastz.

## Availability of supporting data

Data are available via the National Center for Biotechnology Information database, BioProject ID: PRJNA399234, SRA accession number: SRP124668. Supporting data, including the reference assembly and annotations, SNPs, indels, and phylogenetic tree data, are also available via the *GigaScience* repository GigaDB. [59].

## Additional files

**Supplementary Figure S1**. The distribution of the wild and domestic goats used in this study.

**Supplementary Figure S2**. Introduction of wild and domestic goats sequenced in this study.

**Supplementary Figure S3**. Sequencing depth distribution of domestic and wild goats.

**Supplementary Figure S4**. Minor allele frequencies (MAFs) of SNPs among the domestic breeds.

**Supplementary Figure S5**. Ratio of nonsynonymous and synonymous SNPs at different MAFs.

**Supplementary Figure S6**. Ratio of SNP-desert associated nonsynonymous and synonymous SNPs at different MAFs shared among the domestic breeds.

**Supplementary Figure S7**. A Venn diagram of SNPs among the domestic breeds.

**Supplementary Figure S8**. Distribution of total and breed-specific SNPs.

**Supplementary Figure S9**. Distribution of indels found among the domestic breeds.

**Supplementary Figure S10**. Length distribution of indels in domestic population.

**Supplementary Figure S11**. A Venn diagram of domestication-specific short indels.

**Supplementary Figure S12**. A Venn diagram of genes related to domestication-specific CNVs.

**Supplementary Figure S13**. Validation of ASIP copy number in a larger population size from 4 domestic breeds.

**Supplementary Figure S14**. SNP rate distribution with variable sliding windows.

**Supplementary Figure S15**. A Venn diagram of SNP-desert-associated genes (SAGs) in the domestic breeds.

**Supplementary Figure S16**. SNP rate distribution of *RSRC1* gene.

**Supplementary Figure S17**. A Venn diagram of SAGs and domestication-associated genes (DAGs).

**Supplementary Figure S18**. Gene Ontology (GO) enrichment analysis of DAGs.

**Supplementary Figure S19**. Opposite selection for LRP4 in the cashmere and meat breeds.

**Supplementary Figure S20**. A Venn diagram of SAGs, QTL-associated, and trait-associated genes (TAGs).

**Supplementary Figure S21**. GO categories analysis of genes shared by SAGs and QTL

**Supplementary Figure S22**. Association between phenotype and genotype of the 699th nucleotide of FGF5.

**Supplementary Table S1**. Summary of samples and sequencing.

**Supplementary Table S2**. Non-synonymous/synonymous SNP ratios across groups.

**Supplementary Table S3**. Heterozygosity in each individual.

**Supplementary Table S4**. Distribution of breed-specific indels.

**Supplementary Table S5**. Genes with frame-shift of the domestic breeds (specific≥60% mutation rate).

**Supplementary Table S6**. CNV distribution in each individual.

**Supplementary Table S7**. CNV associated genes of domestic breeds (≥60% frequency).

**Supplementary Table S8**. CNV association with economic trait in the dairy breeds.

**Supplementary Table S9**. The distribution of genes in SNP deserts.

**Supplementary Table S10**. GO enrichment of the breed-shared SAGs.

**Supplementary Table S11**. SAGs in the top 10 larger SNP deserts of the cashmere breed.

**Supplementary Table S12**. SAGs in the top 10 larger SNP deserts of the dairy breed.

**Supplementary Table S13**. SAGs in the top 10 larger SNP deserts of the meat breed.

**Supplementary Table S14**. A list of genes overlapping between DAGs and SAGs.

**Supplementary Table S15**. GO enrichment of DAGs.

**Supplementary Table S16**. TAGs in chromosomal regions.

## Abbreviations

AADAC: arylacetamide deacetylase; ADRA2A: alpha-2-adrenergic receptors; AHCY: adenosylhomocysteinase; APOL3: apolipoprotein L3; AR: androgen receptor; ASIP: agouti signaling protein; CNV: copy number variation; DAG: domestic-associated gene; FDR: false discovery rate; FGF5: fibroblast growth factor 5; Fst: fixation index; FXR2: fragile X mental retardation; GDF5: growth differentiation factor 5; GO: gene ontology; Hp: heterozygosity; indel: insertion/deletion; MAFs: minor allele frequencies; MYT1L: myelin transcription factor 1-like; NGS: next-generation sequencing; NME6: NME/NM23 nucleoside diphosphate kinase 6; NR6A1: nuclear receptor subfamily 6 group A member 1; NS/S: the ratio of nonsynonymous to synonymous substitutions; PCA: principal component analysis; PRDM6: PR domain containing 6; QTL: quantitative trait loci; RPL3: ribosomal protein L3; RSRC1: arginine and serine rich coiled-coil 1; SNP: single-nucleotide polymorphism; SAG: SNP desert-associated gene; SHOX2: short stature homeobox 2; SLC26A2: solute carrier family 26 sulfate transporter, member 2 gene; STAT6: signal transducer and activator of transcription 6; STIM1: stromal interaction molecule 1; TAG: trait-associated gene; TNFSF13: tumor necrosis factor (ligand) superfamily, member 13; VPS13C: vacuolar protein sorting 13 homolog C; XP-EHH: cross-population extended haplotype homozygosity; SN: Saanen dairy goats; LN: Liaoning cashmere goats; LZ: Leizhou goats; GZ: Guanzhong goat; IM: Inner Mongolian cashmere goat; HN: Hainan goat; MA: Markhor; SI: Sindh ibex.

## Competing financial interests

The authors declare that they have no competing financial interests.

## Funding

This work was supported by the National Natural Science Foundation of China (grants 31301949, 31272408, 31172184), the National Science Foundation for Post-doctoral Scientists of China (grant 2013M532056), and the Research Fund for the Doctor Program of Higher Education of China (grant 20120204110007).

## Author Contributions

L.S.B., Y.J., Z.H., L.B., and Z.B. designed the experiments and managed the project. C.L., Z.B., F.D.K., L.X.Y., and Y.J. performed the data analysis. A.N., C.H., Y.C.X., Z.X.Y., and H.Y.Z. performed the phenotyping and prepared DNA samples. C.L., F.D.K., G.F.L., and Z.H.B. performed the sequencing, genotyping, and validation. Z.B., C.L., and Y.J. wrote the manuscript.

## Supplementary Material

GIGA-D-17-00226_Original_Submission.pdf

GIGA-D-17-00226_Revision_1.pdf

Response_to_Reviewer_Comments(Original_Submission).pdf

Reviewer_1_Report_(Original_Submission) -- Judit Salces Ortiz 10/24/2017 Reviewed

Reviewer_1_Report_Revision_1 -- Judit Salces Ortiz 04/30/2018 Reviewed

Reviewer_2_Report_(Original_Submission) -- Alessandra Stella 11/16/2017 Reviewed

Reviewer_2_Report_Revision_1 -- Alessandra Stella 06/15/2018 Reviewed

Supplement File
