# Supplementary material for: Genome-wide definition of selective sweeps reveals molecular evidence of trait-driven domestication among elite goat (Capra species) breeds for the production of dairy, cashmere, and meat
Source: Gigascience. 2018 Aug 27;7(12):giy105. doi: 10.1093/gigascience/giy105 (PMC6287099; doi:10.1093/gigascience/giy105)
Supplement: GIGA-D-17-00226_Original_Submission.pdf [file giy105_giga-d-17-00226_original_submission.pdf]

GigaScience

Genome-wide definition of selective sweeps reveals molecular evidence of trait-driven domestication among elite goat (Capra species) breeds for the production of dairy, cashmere, and meat

--Manuscript Draft--

**Manuscript Number:**	GIGA-D-17-00226
**Full Title:**	Genome-wide definition of selective sweeps reveals molecular evidence of trait-driven domestication among elite goat (Capra species) breeds for the production of dairy, cashmere, and meat
**Article Type:**	Research
**Funding Information:**	National Natural Science Foundation of China (CN) (31301949) — Dr. bao zhang<br>National Natural Science Foundation of China (CN) (31272408) — Not applicable<br>National Natural Science Foundation of China (CN) (31172184) — Not applicable<br>National Science Foundation for Post-doctoral Scientists of China (2013M532056) — Dr. bao zhang<br>Research Fund for the Doctor Program of Higher Education of China (20120204110007) — Dr. bao zhang
**Abstract:**	Domestication of wild goats and subsequent intensive trait-driven crossing, inbreeding, and selection, all have led to dramatic phenotypic purification and intermediate breeds for high-quality production of dairy, cashmere, and meat. Genomic re-sequencing provides a powerful mean for direct identification of trait-associated sequence variations that underlie molecular mechanisms of domestication. Here, we report our effort to define such variations based on data from domestic goat breeds (Capra eagagrus hircus; 5 each) selected for dairy, cashmere, and meat production in referencing to their wild ancestors, Sindh ibex (Capra eagagrus blythi; 2) and Markhor (Capra falconeri; 2). Using ~24 million high-quality single nucleotide polymorphisms (SNPs), ~1.9 million insertions/deletions (indels), and 2,317 copy number variations (CNVs), we define SNP-desert-associated genes (SAGs), domestic-associated genes (DAGs), and trait-associated genes (TAGs), and attempt to associate them with quantitative trait loci (QTL), domestication, and agronomic traits. Surprisingly, a greater majority of SAGs shared by all domestic breeds classified into Gene Ontology categories of metabolism and cell cycle. DAGs, together with some SAGs, are most relevant to behavior, immunity, and trait-specificity, whereas TAGs appear directly involved in growth regulation, such as growth differentiation factor 5 (GDF5) and fibroblast growth factor 5 (FGF5) for bone and hair growth, respectively. When investigating divergence of Capra populations, the sequence variations and candidate genes we have identified provide valuable molecular markers for trait-driven genetic mapping and breeding.
**Corresponding Author:**	bin sheng li<br>Xi'an Jiaotong University<br>Xi'an, CHINA
**Corresponding Author Secondary Information:**	
**Corresponding Author's Institution:**	Xi'an Jiaotong University
**Corresponding Author's Secondary Institution:**	
**First Author:**	bao zhang
**First Author Secondary Information:**	

Powered by Editorial Manager® and ProduXion Manager® from Aries Systems Corporation

**Order of Authors:**	bao zhang
	Liao Chang
	Yong Xian Lan
	Nadeem Asif
	Ke Dong Fu
	Bo Li
	Xia Chun Yan
	Bo Hong Zhang
	Yan Xiao Zhang
	Zhen Yong Huang
	Hong Chen
	Jun Yu
	Bin Sheng Li
**Order of Authors Secondary Information:**	
**Opposed Reviewers:**	ping ya zhang<br>Kunming Institute of Zoology Chinese Academy of Sciences<br><br>for direct conflicts of interest
**Additional Information:**	
**Question**	**Response**
Are you submitting this manuscript to a special series or article collection?	No
**Experimental design and statistics**<br><br>Full details of the experimental design and statistical methods used should be given in the Methods section, as detailed in our Minimum Standards Reporting Checklist. Information essential to interpreting the data presented should be made available in the figure legends.<br><br>Have you included all the information requested in your manuscript?	Yes
**Resources**<br><br>A description of all resources used, including antibodies, cell lines, animals and software tools, with enough information to allow them to be uniquely identified, should be included in the Methods section. Authors are strongly encouraged to cite Research Resource Identifiers (RRIDs) for antibodies, model organisms and tools, where possible.	Yes

Powered by Editorial Manager® and ProduXion Manager® from Aries Systems Corporation

Have you included the information requested as detailed in our Minimum Standards Reporting Checklist?	
**Availability of data and materials**<br><br>All datasets and code on which the conclusions of the paper rely must be either included in your submission or deposited in publicly available repositories (where available and ethically appropriate), referencing such data using a unique identifier in the references and in the "Availability of Data and Materials" section of your manuscript.<br><br>Have you have met the above requirement as detailed in our Minimum Standards Reporting Checklist?	Yes

Manuscript Click here to download Manuscript Goat-article.docx

Genome-wide definition of selective sweeps reveals molecular evidence of trait-driven domestication among elite goat (*Capra* species) breeds for the production of dairy, cashmere, and meat

Zhang Bao[1+], Chang Liao[1+], Lan Xianyong[2+], Asif Nadeem[3+], Fu Dongke[1], Guan Fanglin[1], Li Bo[1], Yan Chunxia[1], Zhang Hongbo[1], Zhang Xiaoyan[2], Huang Yongzhen[2], Zhang Hang[1]*, Chen Hong[2]*, Yu Jun[1,4]*, Li Shengbin[1]*

1. Xi'an Jiaotong University, Xi'an 710061, People's Republic of China;
2. Northwest A&F University, Shaanxi Key Laboratory of Molecular Biology for Agriculture, Yangling, Shaanxi 712100, People's Republic of China;
3. Institute of Biochemistry and Biotechnology, University of Veterinary and Animal Sciences, Lahore, Pakistan;
4. CAS Key Laboratory of Beijing Institute of Genomics, Chinese Academy of Sciences, Beijing, China.

+These authors contributed equally to this work

*co-corresponding authors, correspondence and requests for materials should be addressed to: zhanghangmu@hotmail.com; chenhong1212@126.com; junyu@big.ac.cn or shbinlee@mail.xjtu.edu.cn

Abstract:

Domestication of wild goats and subsequent intensive trait-driven crossing, inbreeding, and selection, all have led to dramatic phenotypic purification and intermediate breeds for high-quality production of dairy, cashmere, and meat. Genomic re-sequencing provides a powerful mean for direct identification of trait-associated sequence variations that underlie molecular mechanisms of domestication. Here, we report our effort to define such variations based on data from domestic goat breeds (*Capra eagagrus hircus*; 5 each) selected for dairy, cashmere, and meat production in referencing to their wild ancestors, Sindh ibex (*Capra eagagrus blythi*; 2) and Markhor (*Capra falconeri*; 2). Using ~24 million high-quality single nucleotide polymorphisms (SNPs), ~1.9 million insertions/deletions (indels), and 2,317 copy number variations (CNVs), we define SNP-desert-associated genes

(SAGs), domestic-associated genes (DAGs), and trait-associated genes (TAGs), and attempt to associate them with quantitative trait loci (QTL), domestication, and agronomic traits. Surprisingly, a greater majority of SAGs shared by all domestic breeds classified into Gene Ontology categories of metabolism and cell cycle. DAGs, together with some SAGs, are most relevant to behavior, immunity, and trait-specificity, whereas TAGs appear directly involved in growth regulation, such as growth differentiation factor 5 (*GDF5*) and fibroblast growth factor 5 (*FGF5*) for bone and hair growth, respectively. When investigating divergence of *Capra* populations, the sequence variations and candidate genes we have identified provide valuable molecular markers for trait-driven genetic mapping and breeding.

Keywords: Goat; re-sequencing; trait-driven domestication

Background

As one of the most popular farm mammals, goats (*Capra hircus*) have been domesticated ~10,000 years ago [1]. In early domestication, crucial factors are selected including docility toward human and loss of wild-type behavioral characteristics [2]. Following the initial domestication events of crops in the Fertile Crescent, together with culture diffusion over Europe, Africa, and Asia [3], animal domestication had spread rapidly, as an integral part of the Neolithic Revolution [4]. Once farming developed in the Middle East and Asia ~7,000 B.C., human settlements became permanent and so do domesticated animals to assure better supply of food and clothing [4]. After a long period of so-called *soft* selection, the situation changed dramatically some 200 years ago with the emergence of *breed* concept [3]. Selection had increased intensively on local populations, followed by standardization of trait

performance, and reproductive breeding among breed had seriously reduced, leading to fragmentation of the initial gene pools. More recently, selection pressure has been increased again via the use of artificial insemination, resulting in a few industrial breeds with high trait performance, low effective population size, and profound phenotypic changes [5], such as the case of trait-driven breeding for dairy, cashmere, and meat [6, 7].

Goat, ~800 million in population and ~560 breeds (12 percent of the total recorded mammalian breeds), is the most adaptable livestock in all continents [8] and supplies milk, meat, and fibers for human consumption, and thrives on meager fodder and harsh environments [9]. Despite the importance of this species, the study of goat genomes are still in its infancy as compared to that of other farm animals [10]. Nevertheless, positional cloning demonstrated polled intersex syndrome (PIS) locus locates on 1q43 of the goat genome [11]; transcriptomic studies have paved the way for in-depth genomics, including various trait-relevant tissues, such as the mammary glands, skeletal muscle, and hair follicles; and some genetic studies have also been carried out on traits and disease resistance [12]. Although genome-wide studies of goat QTL and genome sequences have leaped the field forward [13-15], it still lagging behind those of other domestic animals, such as cattle , pig , dog , and chicken .

Our experimental design involves resequencing (~29× in sequencing depth and 99% in genome coverage) of 15 domestic goats representing three breeds as well as 4 wild goats of 2 distinct species. The high-quality sequence data allow us to use high-quality genetic markers (single nucleotide polymorphism, SNP; insertion/deletion, indel; and copy number variation, CNV) to define artificial selection related genes in the history of goat domestication. Particularly, studies of

trait-associated genes (TAGs) provide candidate loci for marker-assisted breeding of domestic goats.

RESULTS AND DISCUSSION

Sequence variations identified in five goat groups

We sequenced three elite domestic goat breeds (5 each), including dairy (Saanen), cashmere (Liaoning Cashmere), and meat (Leizhou), and two wild goat species (Sindh ibex, *Capra aegagrus blythi* and Markhor, *Capra falconeri*; 2 each) as controls (Supplementary Fig. 1). Both Sindh ibex and Markhor are Pakistan wild goats; the latter categorized as endangered on the IUCN Red List (Supplementary Fig. 2). We generated 1,346 Gb (28.8×) and 379 Gb (28.6×) raw data for the domestic and wild goats, respectively (Table 1; Supplementary Table 1 and Supplementary Fig. 3). Referenced to the *Capra hircus* genome (http://goat.kiz.ac.cn/), we identified 23,924,294 SNPs, 1,899,827 indels, and 2,317 CNVs.

Single nucleotide polymorphisms (SNPs)

We analyzed the high-quality SNPs with the criterion of a minimum depth ≥ 8 in every individual sample (Supplementary Fig. 4). In addition, validated our SNP calling accuracy rate is 97.43% (Supplementary Note), using a sequence-capture NGS-based genotyping method (Genesky Biotechnologies, Shanghai, China).

First, the SNPs were partitioned into intergenic (76.20%), intronic (23.06%), and protein-coding (0.74%), and subsequently the ratio of nonsynonymous to synonymous

substitutions (NS/S) was calculated to be 0.95 in average. However, the NS/S ratio shows variable distributions when correlated to minor allele frequency (MAF) in the low SNP rate region or *SNP desert* (Supplementary Table 2; Supplementary Figs. 5 and 6). Second, we identified millions of SNPs within and between the wild and domestic goat groups. Although the number of wild-goat-specific SNPs is smaller than that of the domestic group (5,598,396 vs. 12,434,312 and 6,061,698 shared), this result may reflect biased sampling (15 vs. 4) rather than true genetic heterogeneity of the groups. Third, among the SNPs unique to each domestic breed, the dairy breed appears to have slightly more unique SNPs, indicating recent introduction of genetic heterogeneity, as opposed to the cashmere breed that appears having more in total when breed-shared SNPs are taken into account (Supplementary Fig. 7). At low MAFs, there are higher proportion breed-specific SNPs than the total but a transition at MAF 20%, where the breed-specific SNPs proportion becomes less than that of the total, becomes obvious (Supplementary Fig. 8). In addition, the meat breed has more ancient SNP with higher MAFs than the other two breeds, whereas the cashmere breed is relatively young or less selected as it has more low frequency SNPs (Supplementary Fig. 8). Fourth, we compared heterozygous SNPs across all chromosomes and found that the meat breed has significantly lower heterozygosity (P= 0.0022) as compared to that of two other breeds, suggesting there may be a strong or long-term selection during its breeding (Supplementary Table 3).

Insertions/deletions (indels)

We categorized 1,899,827 indels with nearly equal numbers of insertions and deletions, of which ~0.13% (2,420) are found in protein-coding sequences and partitioned into 32.72% (792) in-frame (3-bp indels) and 1,628 out-of-frame indels that lead to average 499 pseudogenes per individual sample. Similar to the trend observed for SNPs, there are much more rare indels in the total (Supplementary Figs. 9 and 10); we have more indels in domestication-specific than wild-specific (924,352 vs. 363,589), and more indels in the dairy and cashmere breeds as compared to the meat breed (Supplementary Fig. 11 and Supplementary Table 4).

In addition, based on our indel data, *AADAC* (arylacetamide deacetylase) appears selected in the dairy breeds (Supplementary Table 5), encoding an enzyme responsible for hydrolysis of drugs [16]. And the marker of *MYT1L* (myelin transcription factor 1-like) associated with syndromal intellectual disability and early-onset obesity has shown meat-specific high frequency indel frame-shift polymorphism [17].

Copy number variations (CNVs)

We discovered in the 1-kb window, there are 2,028 (246 genes; spanning 32.0-Mb genomic regions) and 1,616 CNVs (144 genes; spanning 19.2-Mb genomic regions) in the domestic and wild goats, respectively. The wild goats and the meat breeds have relatively more numbers of CNVs than the domestic counterparts and two other domestic breeds, respectively (Supplementary Table 6). The meat goats have more breed-specific CNVs as compared to dairy and cashmere breeds (38 vs. 33 and 22) and 11 CNVs shared by all three breeds (Supplementary Fig. 12).

Meanwhile, consistent with previous study [18], we revealed high frequency domestication-specific CNVs in *ASIP* (agouti signaling protein) and *AHCY* (adenosylhomocysteinase), both are related to skin pigmentation and coat color [19]. Interestingly, goats with white hairs (Saanen and Liaoning Cashmere goats) have

much more copies of *ASIP* and *AHCY* than those with colored hairs (Leizhou and wild goats; Supplementary Table 7). We have confirmed this result in a larger population sampling with white and black coat population (n= 54; Supplementary Fig. 13).

Finally, in order to validate if CNV loci associated with dairy traits, we chose 12 candidate dairy specific CNV loci to detect in Guanzhong Dairy goat. Our CNV association study points to two CNV loci (including *APOL3* and *NEM6*; *P*<0.01) for dairy and growth traits (Supplementary Table 8). *APOL3* (apolipoprotein L3) is a lipid transport and metabolism associated gene also highly duplicated in the beef breeds [20], and *NME6* (NME/NM23 nucleoside diphosphate kinase 6) is suggested to play a role in cell growth and cell cycle [21].

Population structures of domestic and wild goats

We used similarity analysis, principal components analysis (PCA), and phylogeny reconstruction to evaluate the population structure of the domestic breeds. Based on genome-wide SNPs, we found that Sindh ibex are genetically closer to the domestic breeds as compared to Markhor, consistent with the previous report [22]. Our PCA result indicates that the domestic breeds and the wild breeds are three distinct groups, and the same is true for the three domestic breeds (Fig. 1a). Our neighbor-joining tree shows that the two Chinese domestic breeds are closer to each other and the Saanen breed is rather close to the wild goats, and all results suggest that the domestication traits of dairy production may occur ahead of cashmere and meat in the domestication history of goats (Fig. 1).

SNP-desert-associated genes (SAGs)

SNP deserts are often linked to beneficial mutations as selective sweeps that are

subjected to strong purifying selection [23]. The SNP deserts defined as genomic regions with lowest 10% SNP rates (10 kb windows). SNP-desert-associated genes (SAGs) are selected which are harbored by SNP deserts (>30%; Fig. 2). We noticed that there is a bimodal SNP rate only distribution in the dairy and meat breeds; the largely absence of SNP-poor regions suggests the effect of both stronger recent purifying selection and lack of recent introduction of genetic heterogeneity in the cashmere breed as compared to the two domestic breeds. In addition, the lower mean and median SNP rates of the meat breed (Fig. 2a and Supplementary Fig. 14) suggest overall poorer genetic heterogeneity or heavier inbreeding. In total, 277.39 Mb (3,950 SAGs), 278.33 Mb (4,395 SAGs), and 273 Mb regions (3,447 SAGs) SNP deserts were detected for the dairy, cashmere, and meat goat genomes, respectively (Fig. 2b and Supplementary Table 9). For 1,196 SAGs shared among the domestic breeds, Gene Ontology (GO) enrichment shows only two major categories: metabolism and cell cycle regulation (Supplementary Fig. 15a and Supplementary Table 10).

To look into more details, we also examined the large SNP deserts (>100 kb in length) as well as the top 10 larger deserts unique to each domestic breed. For the SNP deserts >100 kb in length, it is consistent that the dairy and cashmere breeds have more SAGs than the meat breed (1,044 vs. 1,112 and 1,503 in the dairy and cashmere breeds, respectively); the function of the 231 breed-shared SAGs appears all related to signal transduction (such as *RSRC1*, Fig. 2c and Supplementary Fig. 15b). To provide alternative insights, we scrutinized the top 10 SNP deserts in three breeds. When looking for breed-shared SAGs in the top 10, we only have one SNP desert including *AR* gene on chr X. *AR* (androgen receptor) is a hormone-inducible DNA-binding transcription factor, which plays an essential role in male reproduction and its knock-out male mice display severely impaired reproductive tract and sexual

behavior [24]. For the top 10 SNP deserts found in each breed, the meat and dairy breeds both have unique loci to their own, four for the meat and two for the dairy, but none for the cashmere breed (Supplementary Discussion, Supplementary Table 11, 12, 13).

Domestication-associated genes (DAGs)

To detect the sequence signature of selective sweeps over large genomic regions, we first calculate pooled heterozygosity (Hp), using autosomal SNPs from all individuals of the domestic breeds in a 100-kb sliding window. We also calculate fixation index (Fst), denoting population differentiation, between the domestic and the wild populations in a 100-kb sliding window based on autosomal SNPs. We at the end transform the Hp and Fst into Z (Hp) and Z (Fst), respectively, and the protocol defined 67 domestication-associated genes (DAGs) in a collective genomic length of 3.2 Mb (Fig.3).

Of the 67 DAGs, all of them overlap with SAGs in one, two, or three goat breeds (Supplementary Fig. 16 and Supplementary Table 14) and our GO enrichment analysis indicates that the significant categories (FDR q<0.001) are negative regulation of gene expression and protein import into nucleus, docking (Supplementary Fig. 17 and Supplementary Table 15). This set of genes may contribute to behavior, immunity, and morphological differences between domestic and wild goats. First, genes directly influencing nervous system and behavior include *ADRA2A* (alpha-2-adrenergic receptors, which regulate neurotransmitter release), and *FXR2* (fragile X mental retardation, autosomal homolog 2, which required for the presence of behavioral circadian rhythms) [25, 26]. Second, *TNFSF13* (tumor necrosis factor (ligand) superfamily, member 13) and *STIM1* (stromal interaction

molecule 1) regulate B-cell development and T cell-mediated immune regulation during chronic infection [27, 28], respectively. Third, morphological difference involves genes: *NR6A1* (Nuclear Receptor Subfamily 6 Group A Member 1), which affect the number of vertebrates, and as one of the most characteristic morphological changes in domestic pigs [29, 30]; *STAT6* (signal transducer and activator of transcription 6), is associated with body weight as well as carcass and growth efficiency traits [31]. All these genes are appealing candidates for further investigation (Supplementary Discussion).

TAGs (Trait-associated genes) of domestic breeds

To uncover genetic variants involved in local adaptation and selection in the three breeds, we performed Fst and the Hudson-Kreitman-Aguade (HKA in a 100-kb window) test on SNPs from one breed against the pooled from two other breeds. Using the criterion of Fst>4 and HKA *P*<0.05, we defined 66 trait-associated genes (TAGs) with and cross-referenced co-localization data with sheep QTL [32] and SAGs (Fig. 4, Supplementary Table 16 and 17). Striking correlation detected between putative selective sweeps and SNP-deserts; there are 660, 912, and 1,841 genes shared by SAGs and QTLs unique to the dairy, cashmere, and meat breeds, respectively (Supplementary Fig.18). Most of these genes enriched in GO categories of metabolic process, biological regulation, and response to stimulus, whereas the trait-specific categories include reproduction and growth (Supplementary Fig. 19). In addition, GO enrichment analysis of 66 TAGs indicates that the significant categories (FDR q<0.001) are mitochondrial respiratory chain complex assembly, nucleoside transmembrane transport, and DNA recombination (Supplementary Fig. 20 and Supplementary Table 18).

TAGs in the dairy breed

The TAGs and their functions found in the dairy breeds are represented by several important genes, only *BRIP1* (BRCA1-interacting protein 1) is shared by TAGs, SAGs, and QTL (Supplementary Fig. 18a). *BRIPI* encodes a helicase interacting with *BRCA1* germline, mutations of these two genes are both confer high risk of breast and ovarian cancer [33]. Many other TAGs are associated with milk trait and growing development. For instance, *RPL3* (ribosomal protein L3) is reported to be highly expressed in breast milk fat globule, suggesting that translational pressure is at work during lactation [34]. In addition, *RPL3* is indicated to play a role in regulation of energy balance [35]. One other gene, *VPS13C* (vacuolar protein sorting 13 homolog C) is suggested to act on glucose homeostasis for high milk production in dairy cows [36], and another member of the same gene family, *VPS13A*, has also been reported in pigs undergoing directional selection for heat adaptation [37]. We also noted two development related genes; *GDF5* (growth differentiation factor 5), a member of the TGF-beta superfamily, and *SHOX2* (short stature homeobox 2), are both involved in height and chondrogenesis [38, 39] (Fig. 4a).

To further explore artificial selection related genes in the dairy breed, we scanned genotype frequency at non-synonymous sites within these genes and found genotype TT at 217th nucleotide of the *GDF5* is close to fixation in the dairy breeds. At this position, variation T217C generates an amino acid variation R73G; R is conserving among all other known mammal sequences but not in goats. In 15 sequencing individuals, genotypes TT and CC dominate in the dairy and meat breeds, respectively. Moreover, the validation data from six breeds of 287 domestic goats showed different genotype frequencies among three breeds (Fig. 5). Genotypes at this locus may relate

to body size since the dairy breeds have the largest body weight and highest body length as opposed to the two other breeds (Supplementary Fig. 2). Therefore, this missense mutation may be functionally causative.

TAGs in the cashmere breed

Only three genes, *POLD2*, *AEBP1*, and goat_GLEAN_10005251, shared by TAGs, SAGs, and QTL (Supplementary Fig. 18b). *POLD2* (DNA polymerase delta 2, accessory subunit) required for the stimulation of DNA polymerase delta activity [40]; *AEBP1* (adipocyte enhancer binding protein 1) modulated adiposity and energy homeostasis, and functioned in wound healing and abdominal wall development [41](Fig. 4b). Among other trait-related genes, *FGF5* (fibroblast growth factor 5) stands out because it is an inhibitor of hair elongation and associated with hair growth and length in mammals [14, 42, 43]. To further annotate the goat *FGF5*, we performed an association analysis between exonic mutations and cashmere related traits in 224 Inner Monglia Cashmere goats. Cashmere production and body weight appear associated with one synonymous mutation in the exon 3 of *FGF5* (Supplementary Fig. 21). Moreover, a region on chr 10 with strong support contains *PRDM6* (PR domain containing 6), belonging to the PRDM family of transcriptional repressors, reported to be highly expressed in NOTCH1-deficient mice embryos. We expected *PRDM6* as a candidate gene for the cashmere trait because *NOTCH1* is considered controlling follicular proliferation rates and melanocyte population [44].

TAGs in the meat breed

The most important TAGs to the meat breed are *LRP4*, *FAM150B*, *RABL2B*, and *CKAP5* (Supplementary Fig. 18c). *LRP4* (low density lipoprotein receptor-related protein 4) is detected with a conserved amino acid changed in goat, and it also showed

opposite selection in the meat and other goat breeds when validate in more goat populations (Supplementary Fig. 22). This gene is reported to be a critical player in bone-mass homeostasis due to its function as a receptor of sclerostin to inhibit Wnt/β-catenin signaling and bone formation [45]. *CKAP5* (cytoskeleton associated protein 5) is one of the major spindle proteins with important roles in mitosis [46]. Several other TAGs including *MYOF* (myoferlin), *GRP* (gastrin-releasing peptide), and *ACP1* (adipocyte acid phosphatase) are associated with growth or energy balance [47, 48] (Fig. 4c).

CONCLUSION

In this study, we interrogated whole genome sequences from three trait-driven goat breeds, assessed three categories of sequence variations–SNPs, indels, and CNVs –to look for functional relevance of three categories of candidate genes– SAGs, DAGs, and TAGs. First, we used several methods, including SNP desert, fixation index, and pooled heterozygosity, to define these candidate genes based on allelic frequencies of the different sequence variations. Although the sampling itself is rather limited for each breed, a number of following-up studies with increased population sampling have showed consistent results. Second, we grouped the breeds and the data in various ways for detailed analyses, *casting larger net* sometimes (such as SNP desert and QTL data) and looking into discrete lists in other cases, trying to provide an overview of the genetic landscape of selection-centric genetic heterogeneity in the up-to-date molecular terminology. Third, the candidate genes we elaborated as DAGs and TAGs are complex in function but clearly biased toward certain functional categories. To validate them in a larger population of a specialized breed is of essence before any mechanistic studies. Finally, NGS technology provides an efficient tool for systematically deciphering genetic background of domestication and trait selection in

a thorough way for goats and other farmed animals, and we should heckle gene ontology and expression information at the same time while we are expecting thousands of gene sequences becoming available in the years to come.

METHODS

Sample collection and sequencing

We sequenced DNA samples from 19 goats: 4 from wild goats (2 Markhor and 2 Sindh ibex) and 15 from domestic goats (5 Saanen, 5 Liaoning Cashmere and 5 Leizhou goats). To validate the sequence variations at population level, we genotyped by sequencing PCR amplified fragments 7 domestic breeds, including 99 Saanen, 85 Liaoning Cashmere, 23 Leizhou, 16 Dera Din Panah, 30 Guanzhong, 26 Inner Mongolia Cashmere, and 24 Hainan goats. The samples of Markhor and Sind ibex were collected from skin biopsies, Quetta, Pakistan. Blood samples of the domestic goats were collected in China. DNA sequences were acquired by using paired-end sequencing technology on the Illumina HiSeq x10 platform. The institutional review board of the Xi'an Jiaotong University Health Science Center with project identification code (2011-054) approved the study protocol.

Processing raw reads

The procedure to remove low-quality reads meets one or more of the following criteria: 1) N-content more than 10%; 2) >60% read length below Q7; 3) reads overlapping >10 bp with the adapter sequence and a maximum of 2 bp mismatches to the adaptor sequence; 4) paired-end reads overlapped by >10 bp with others; and 5) duplicated reads. We also trimmed up to 10 bp at the 5'-end or 30 bp at the 3'-end of a read if the local N-content is >20%.

Read mapping and quality control

We used BWA 0.5.9 to map the clean reads onto the reference genome of Capra hircus genome V1 (http://goat.kiz.ac.cn/). The command ‘aln -t 4 -e 10’ was used to find the suffix array coordinates of good hits of each read. Then we used the command ‘sample -a 500’ to convert suffix array coordinates into chromosomal coordinates and paired reads. Other parameters were set to the defaults. We filtered the alignments as follows: 1) mapping quality score lower than 20; 2) non-unique alignments; and 3) duplicated alignments.

Calling and validation of SNPs, indels, and CNVs

First, SNP was called in population scale using ANGSD with the parameters referring a previous publication [49]. And we filtered out the locus with minimum depth <8 in all individuals, and call a heterozygous SNP in one individual only when both allele are supported by at least 4 reads. We validated SNP calling rate (97.43%), using a sequence-capture NGS-based genotyping method by Genesky Biotechnologies (Shanghai, China). Second, Dindel v1.01 was used to call short indels (1-5bp) in each individual [50]. We call an indel only when the non-ref allele is covered by at least 2 read on each strand. Then we filtered out the results which met one or more of the following three criteria: quality reported by Dindel below 20, reference homopolymer length longer than 10 bp, and length of insertion or deletion longer than 5 bp. Third, software Control-FREEC was used to detect copy number variation (CNV) based on pair wise comparison [51]. With 1kb window, we compared the coverage depth between the window and the average depth and identified CNV regions different from the reference. We merged the overlapped CNV regions among different samples.

Population structure analysis

We performed principle component analysis (PCA) with all population-scale autosomal SNPs using the package EIGENSOFT. The phylogenetic tree was constructed based on all autosomal SNPs, with the evolutionary distances measured by p-distance with PHYLIP. In addition, FRAPPE was used to investigate the population structure. We ran 10,000 iterations and the number of clusters from 2 to 5.

Definition of SNP deserts

Based on the SNP data, we computed SNP rate in 10 kb sliding windows. We normalized the SNP rates over the length of ≥8 depth aligned sequence in each bin rather than the bin size, and bins with less than 1 kb of aligned sequence were rejected. We then pick out the windows with lowest 10% SNP rate of the genome data and join up these windows as longer region if the gap between them ≤10kb. We defined these low SNP-rate window or region as “SNP desert”.

Selection analysis

To find selective sweep in domestic lines, Hp and Fst were used to extract outliers. For each 100-kb window, we determined the number of reads corresponding to the most and least abundant SNP alleles (nMAJ and nMIN), $Hp=2\sum nMAJ\sum nMIN/(\sum nMAJ+\sum nMIN)^2$. With the same 100kb window, the fixation index (Fst) was calculated between 15 domestic and 4 wild goats. We then transformed Hp into ZHp: $ZHp= (Hp-\mu Hp)/ \sigma Hp$, Fst into ZFst: $ZFst= (Fst-\mu Fst)/ \sigma Fst$. For DAG analysis, we applied a threshold of ZHp = -3 OR ZFst = 3 for detecting putative selective sweeps. For TAG analysis, we measured the pairwise Fst and test the between one domestic breed and pool of other two breeds. The windows pass the threshold of ZFst = 4 and *P*-value < 0.05 by HKA test were extracted as candidate selective sweep regions. Genes residing in these extracted regions were

indicated as candidate-selected genes.

QTL mapping

We downloaded known sheep QTL data from Animal QTLdb and qualified the data by filtering out the terms with “trait association” or with P>0.05. We aligned the genome sequences of sheep and goat with lastz (version 1.02.00) and mapped the sheep QTL to goat chromosomes based on the axt file produced by lastz.

Competing financial interests

The authors declare no competing financial interests.

ACKNOWLEDGMENTS

This work supported by the National Natural Science Foundation of China (Grant No. 31301949, No. 31272408, No. 31172184); National Science Foundation for Post-doctoral Scientists of China (Grant No.2013M532056); and Research Fund for the Doctor Program of Higher Education of China (No.20120204110007).

AUTHORS CONTRIBUTIONS

Li. S.B., Yu.J., Zhang. H., Li. B., and Zhang. B. designed the experiments and managed the project.

Chang. L., Zhang. B., Fu. D.K., Lan. X.Y, and Yu. J. performed the data analysis. Asif. N, Chen. H., Lai. J.H., Chen. T., Yan. C.X., Zhang. X.Y., Huang. Y.Z. performed the phenotyping and prepared DNA samples. Chang. L., Fu. D.K., Guan. F.L., and Wei. S.G., Zhang. H.B., Yu. B. performed the sequencing, genotyping and validation. Zhang. B., Chang. L., Yu. J., and Li. B., wrote the manuscript.

Data Accessibility:

Our data are submitted to NCBI database, BioProject ID: PRJNA399234.

REFERENCES

1. M A Zeder, and Hesse, B. The Initial Domestication of Goats (Capra hircus) in the Zagros Mountains 10,000 Years Ago. *Science* 2000; *287*: 2254-7.
2. L Trut, Oskina, I, and Kharlamova, A. Animal evolution during domestication: the domesticated fox as a model. *Bioessays* 2009; *31*: 349-60.
3. P Taberlet, Valentini, A, Rezaei, H R, et al. Are cattle, sheep, and goats endangered species? *Mol Ecol* 2008; *17*: 275-84.
4. Y Hatziminaoglou, and Boyazoglu, J. The goat in ancient civilisations: from the Fertile Crescent to the Aegean Sea. *Small Ruminant Research* 2004; *51*: 123-9.
5. P Taberlet, Coissac, E, Pansu, J, et al. Conservation genetics of cattle, sheep, and goats. *C R Biol* 2011; *334*: 247-54.
6. C J Rubin, Zody, M C, Eriksson, J, et al. Whole-genome resequencing reveals loci under selection during chicken domestication. *Nature* 2010; *464*: 587-91.
7. J E Decker, McKay, S D, Rolf, M M, et al. Worldwide patterns of ancestry, divergence, and admixture in domesticated cattle. *PLoS Genet* 2014; *10*: e1004254.
8. FAO. The State of the World's Animal Genetic Resources for Food and Agriculture. 2007.
9. D E MacHugh, and Bradley, D G. Livestock genetic origins: goats buck the trend. *Proc Natl Acad Sci U S A* 2001; *98*: 5382-4.
10. L Fontanesi, Martelli, P L, Beretti, F, et al. An initial comparative map of copy number variations in the goat (Capra hircus) genome. *BMC Genomics* 2010; *11*: 639.
11. L Schibler, Cribiu, E P, Oustry-Vaiman, A, et al. Fine mapping suggests that the goat Polled Intersex Syndrome and the human Blepharophimosis Ptosis Epicanthus Syndrome map to a 100-kb homologous region. *Genome research* 2000; *10*: 311-8.
12. P Aguilar-Calvo, Fast, C, Tauscher, K, et al. Effect of Q211 and K222 PRNP Polymorphic Variants in the Susceptibility of Goats to Oral Infection With Goat Bovine Spongiform Encephalopathy. *J Infect Dis* 2015.
13. Y Dong, Xie, M, Jiang, Y, et al. Sequencing and automated whole-genome optical mapping of the genome of a domestic goat (Capra hircus). *Nat Biotechnol* 2013; *31*: 135-41.
14. X Wang, Liu, J, Zhou, G, et al. Whole-genome sequencing of eight goat populations for the detection of selection signatures underlying production and adaptive traits. *Scientific reports* 2016; *6*: 38932.
15. D M Bickhart, Rosen, B D, Koren, S, et al. Single-molecule sequencing and chromatin conformation capture enable de novo reference assembly of the domestic goat genome. *Nature genetics* 2017; *49*: 643-50.
16. M Shimizu, Fukami, T, Kobayashi, Y, et al. A novel polymorphic allele of human arylacetamide deacetylase leads to decreased enzyme activity. *Drug Metab Dispos* 2012; *40*: 1183-90.
17. N De Rocker, Vergult, S, Koolen, D, et al. Refinement of the critical 2p25.3 deletion region: the role of MYT1L in intellectual disability and obesity. *Genet Med* 2015; *17*: 460-6.
18. Y Dong, Zhang, X, Xie, M, et al. Reference genome of wild goat (capra aegagrus) and sequencing of goat breeds provide insight into genic basis of goat domestication. *BMC Genomics* 2015; *16*: 431.
19. B J Norris, and Whan, V A. A gene duplication affecting expression of the ovine ASIP gene is responsible for white and black sheep. *Genome research* 2008; *18*: 1282-93.
20. D M Bickhart, Hou, Y, Schroeder, S G, et al. Copy number variation of individual cattle genomes using next-generation sequencing. *Genome research* 2012; *22*: 778-90.
21. H Tsuiki, Nitta, M, Furuya, A, et al. A novel human nucleoside diphosphate (NDP) kinase, Nm23-H6, localizes in mitochondria and affects cytokinesis. *J Cell Biochem* 1999; *76*: 254-69.
22. G Luikart, Gielly, L, Excoffier, L, et al. Multiple maternal origins and weak phylogeographic structure in domestic goats. *Proc Natl Acad Sci U S A* 2001; *98*: 5927-32.
23. L Wang, Hao, L, Li, X, et al. SNP deserts of Asian cultivated rice: genomic regions under domestication. *J Evol Biol* 2009; *22*: 751-61.

24. T Matsumoto, Sakari, M, Okada, M, et al. The androgen receptor in health and disease. *Annu Rev Physiol* 2013; *75*: 201-24.

25. J Zhang, Fang, Z, Jud, C, et al. Fragile X-related proteins regulate mammalian circadian behavioral rhythms. *Am J Hum Genet* 2008; *83*: 43-52.

26. J Maestu, Allik, J, Merenakk, L, et al. Associations between an alpha 2A adrenergic receptor gene polymorphism and adolescent personality. *Am J Med Genet B Neuropsychiatr Genet* 2008; *147B*: 418-23.

27. L Desvignes, Weidinger, C, Shaw, P, et al. STIM1 controls T cell-mediated immune regulation and inflammation in chronic infection. *J Clin Invest* 2015; *125*: 2347-62.

28. S R Dillon, Gross, J A, Ansell, S M, et al. An APRIL to remember: novel TNF ligands as therapeutic targets. *Nat Rev Drug Discov* 2006; *5*: 235-46.

29. C J Rubin, Megens, H J, Martinez Barrio, A, et al. Strong signatures of selection in the domestic pig genome. *Proc Natl Acad Sci U S A* 2012; *109*: 19529-36.

30. S Mikawa, Morozumi, T, Shimanuki, S, et al. Fine mapping of a swine quantitative trait locus for number of vertebrae and analysis of an orphan nuclear receptor, germ cell nuclear factor (NR6A1). *Genome research* 2007; *17*: 586-93.

31. G Rincon, Farber, E A, Farber, C R, et al. Polymorphisms in the STAT6 gene and their association with carcass traits in feedlot cattle. *Anim Genet* 2009; *40*: 878-82.

32. Z L Hu, Park, C A, Wu, X L, et al. Animal QTLdb: an improved database tool for livestock animal QTL/association data dissemination in the post-genome era. *Nucleic Acids Res* 2013; *41*: D871-9.

33. K Daino, Imaoka, T, Morioka, T, et al. Loss of the BRCA1-interacting helicase BRIP1 results in abnormal mammary acinar morphogenesis. *PLoS One* 2013; *8*: e74013.

34. S R Wesolowski, Allan, M F, Nielsen, M K, et al. Evaluation of hypothalamic gene expression in mice divergently selected for heat loss. *Physiol Genomics* 2003; *13*: 129-37.

35. M F Allan, Nielsen, M K, and Pomp, D. Gene expression in hypothalamus and brown adipose tissue of mice divergently selected for heat loss. *Physiol Genomics* 2000; *3*: 149-56.

36. C O Lemley, Butler, S T, Butler, W R, et al. Short communication: insulin alters hepatic progesterone catabolic enzymes cytochrome P450 2C and 3A in dairy cows. *J Dairy Sci* 2008; *91*: 641-5.

37. H Ai, Fang, X, Yang, B, et al. Adaptation and possible ancient interspecies introgression in pigs identified by whole-genome sequencing. *Nature genetics* 2015; *47*: 217-25.

38. J Cobb, Dierich, A, Huss-Garcia, Y, et al. A mouse model for human short-stature syndromes identifies Shox2 as an upstream regulator of Runx2 during long-bone development. *Proc Natl Acad Sci U S A* 2006; *103*: 4511-5.

39. S Sanna, Jackson, A U, Nagaraja, R, et al. Common variants in the GDF5-UQCC region are associated with variation in human height. *Nature genetics* 2008; *40*: 198-203.

40. R Hindges, and Hubscher, U. Cloning, chromosomal localization, and interspecies interaction of mouse DNA polymerase delta small subunit (PolD2). *Genomics* 1997; *44*: 45-51.

41. M D Layne, Yet, S F, Maemura, K, et al. Impaired abdominal wall development and deficient wound healing in mice lacking aortic carboxypeptidase-like protein. *Mol Cell Biol* 2001; *21*: 5256-61.

42. C A Higgins, Petukhova, L, Harel, S, et al. FGF5 is a crucial regulator of hair length in humans. *Proc Natl Acad Sci U S A* 2014; *111*: 10648-53.

43. J M Hebert, Rosenquist, T, Gotz, J, et al. FGF5 as a regulator of the hair growth cycle: evidence from targeted and spontaneous mutations. *Cell* 1994; *78*: 1017-25.

44. J Lee, Basak, J M, Demehri, S, et al. Bi-compartmental communication contributes to the opposite proliferative behavior of Notch1-deficient hair follicle and epidermal keratinocytes. *Development* 2007; *134*: 2795-806.

45. L Xiong, Jung, J U, Wu, H, et al. Lrp4 in osteoblasts suppresses bone formation and promotes osteoclastogenesis and bone resorption. *Proc Natl Acad Sci U S A* 2015; *112*: 3487-92.

46. J Al-Bassam, and Chang, F. Regulation of microtubule dynamics by TOG-domain proteins XMAP215/Dis1 and CLASP. *Trends Cell Biol* 2011; *21*: 604-14.

47. A R Demonbreun, Posey, A D, Heretis, K, et al. Myoferlin is required for insulin-like growth

factor response and muscle growth. *FASEB J* 2010; *24*: 1284-95.

48. A I Sayegh. The role of bombesin and bombesin-related peptides in the short-term control of food intake. *Prog Mol Biol Transl Sci* 2013; *114*: 343-70.

49. S Liu, Lorenzen, E D, Fumagalli, M, et al. Population genomics reveal recent speciation and rapid evolutionary adaptation in polar bears. *Cell* 2014; *157*: 785-94.

50. C A Albers, Lunter, G, MacArthur, D G, et al. Dindel: accurate indel calls from short-read data. *Genome research* 2011; *21*: 961-73.

51. V Boeva, Popova, T, Bleakley, K, et al. Control-FREEC: a tool for assessing copy number and allelic content using next-generation sequencing data. *Bioinformatics* 2012; *28*: 423-5.

Figures and Tables Legends

Figure 1 Phylogeny and population structure of goat. (**a**) PCA based on all identified autosomal SNPs. (**b**) Neighbor-joining tree based on autosomal SNPs. SN: Saanen dairy goats, LN: Liaoning Cashmere goats, and LZ: Leizhou goats. Markhor and Sindh ibex are wild goat ancestors.

Figure 2 SNP deserts of three domesticate breeds. (**a**) SNP rate distribution. Mean and median SNP rates are labeled by the peaks of distributions. (**b**) SNP desert length distribution. (**c**) *RSRC1* is in a SNP desert region shared by all three breeds. SN: Saanen dairy goats, LN: Liaoning Cashmere goats, and LZ: Leizhou goats.

Figure 3 Candidate regions for domestication-associated genes (DAGs). (**a**) Distribution of Z-transformed pooled heterozygosity (ZHp) in the 15 domestic goats; and Z-transformed fixation index (ZFst) between wild and domestic goats for autosomes 1 to 29. The red vertical lines indicate the thresholds. (**b**) The positive end of the ZFst distribution (ZFst>3) and the negative end of the ZHp distribution (ZHp<-3) used for extracting outliers. The dashed lines indicate cutoff values. DAGs labeled are discussed in the text.

Figure 4 Candidate selective sweep analysis of each economic breed. Selective sweeps and their associated genes are showed in the three breeds: (**a**) Saanen breed, (**b**) Liaoning Cashmere breed, and (**c**) Leizhou breed. The windows passed the threshold ZFst >4 and *P*<0.05 in HKA test are extracted as selective sweeps. TAGs

labeled in color are discussed in the text.

Figure 5 Opposite selection for sites in *GDF5* of the dairy and meat breeds. (**a**) A nonsysnonmous SNP T217C (R73G) is located in the first exon of *GDF5*. The amino acids at this position are highly conserved in other mammals. (**b**) Frequency divergence happened in different economically relevant traits. Allele T is dominant in the dairy breed (Saanen and Guanzhong), while C is dominant in the meat breed (Leizhou and Hainan). SN: Saanen goat, LN: Liaoning Cashmere goat, LZ: Leizhou goat, GZ: Guanzhong goat, IM: Inner Mongolia Cashmere goat, HN: Hainan goat.

Table 1 Summary of sequencing and variations for domestic and wild goats.

Group	N	Raw data (Gb)	Average Uniquely mapped bases (Gb)	Map ping rate	Mean depth	Total SNP ($x10^6$)	Total SNP NS/S	Indel # ($x10^6$)	CNV #	CNV length (Mb)
**Domestic**	15	89.75	63.43	70.67	28.84	19.04	0.86	1.54	2,028	32.0
Dairy_SN	5	89.23	67.79	75.97	29.11	11.38	0.82	1.02	1,161	20.5
Cashmere_LN	5	88.46	67.33	76.11	28.46	12.33	0.83	1.02	1,096	18.5
Meat_LZ	5	91.56	68.36	74.66	28.96	9.19	0.85	0.83	1,725	21.8
**Wild**	4	94.72	67.29	71.04	28.64	11.66	0.97	0.99	1,616	19.2
Markhor	2	86.27	62.71	72.69	26.89	4.42	0.87	0.64	1,220	15.4
Sindh ibex	2	103.16	71.87	69.66	30.39	7.63	1.06	0.73	1,352	15.3
**Total**	19	90.80	64.24	70.75	28.80	23.92	0.95	1.90	2,317	35.5

Note: The locations where the goat breeds are farmed are also labeled, Saanen or SN, Liaoning or LN, and Leizhou or LZ. The ration of synonymous and non-synonymous SNPs are listed under NS/S.

Click here to download Figure Figure 1.docx

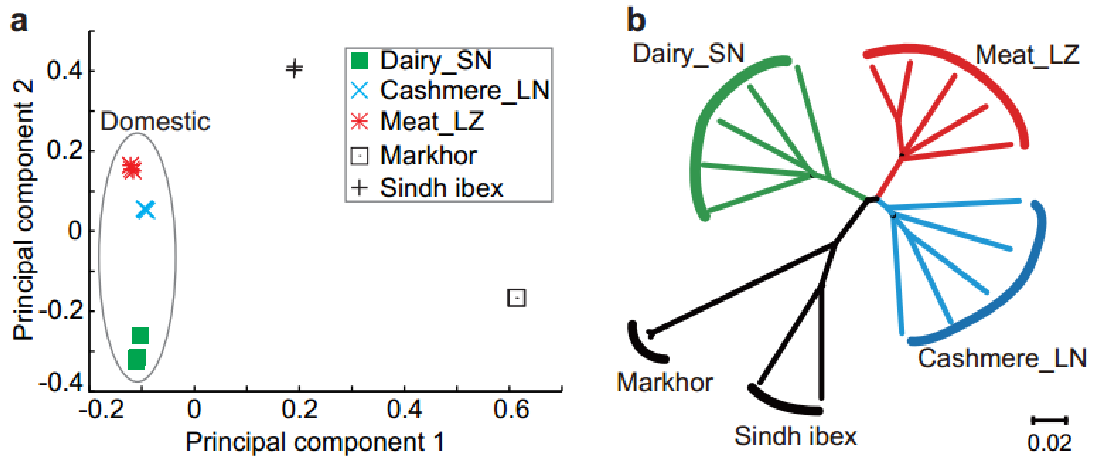

Figure 1 Phylogeny and population structure of goat. (**a**) PCA based on all identified autosomal SNPs. (**b**) Neighbor-joining tree based on autosomal SNPs. SN: Saanen dairy goats, LN: Liaoning Cashmere goats, and LZ: Leizhou goats. Markhor and Sindh ibex are wild goat ancestors.

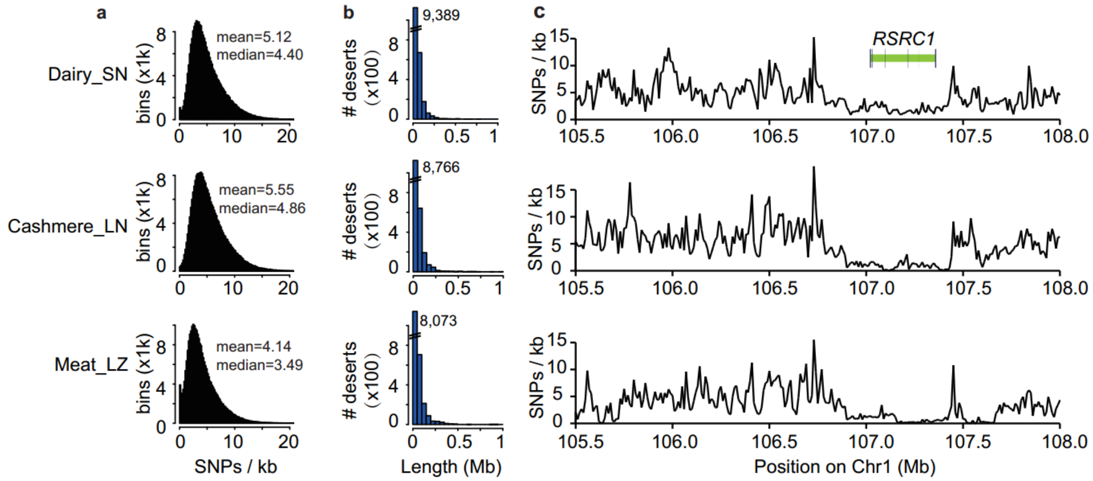

Figure 2 SNP deserts of three domesticate breeds. (a) SNP rate distribution. The peaks of distributions label mean and median SNP rates. **(b)** SNP desert length distribution. **(c)** *RSRC1* is in a SNP desert region shared by all three breeds. SN: Saanen dairy goats, LN: Liaoning Cashmere goats, and LZ: Leizhou goats.

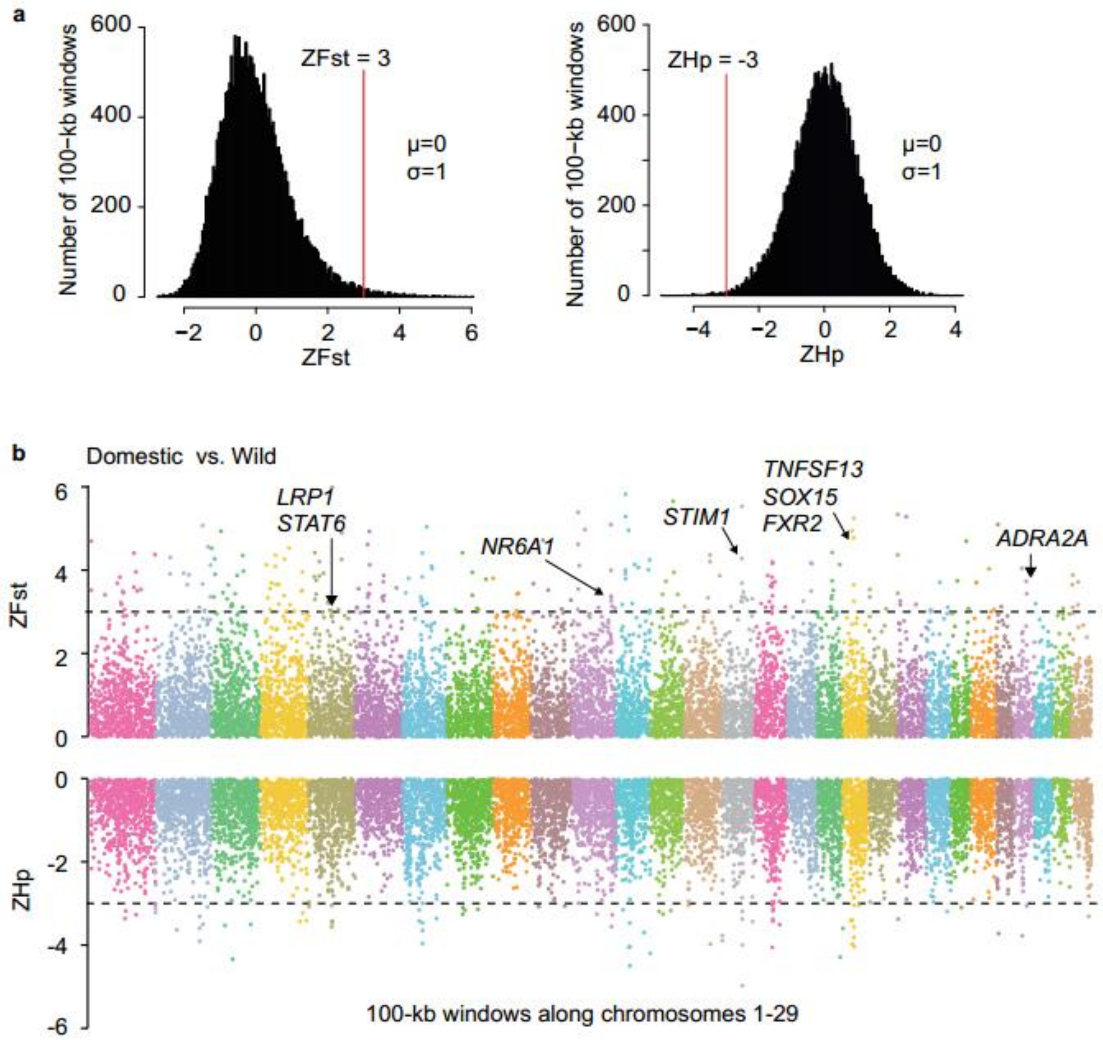

Figure 3 Candidate regions for domestication-associated genes (DAGs). (a) Distribution of Z-transformed pooled heterozygosity (ZHp) in the 15 domestic goats; and Z-transformed fixation index (ZFst) between wild and domestic goats for autosomes 1 to 29. The red vertical lines indicate the thresholds. **(b)** The positive end of the ZFst distribution (ZFst>3) and the negative end of the ZHp distribution (ZHp<-3) used for extracting outliers. The dashed lines indicate cutoff values. DAGs labeled are discussed in the text.

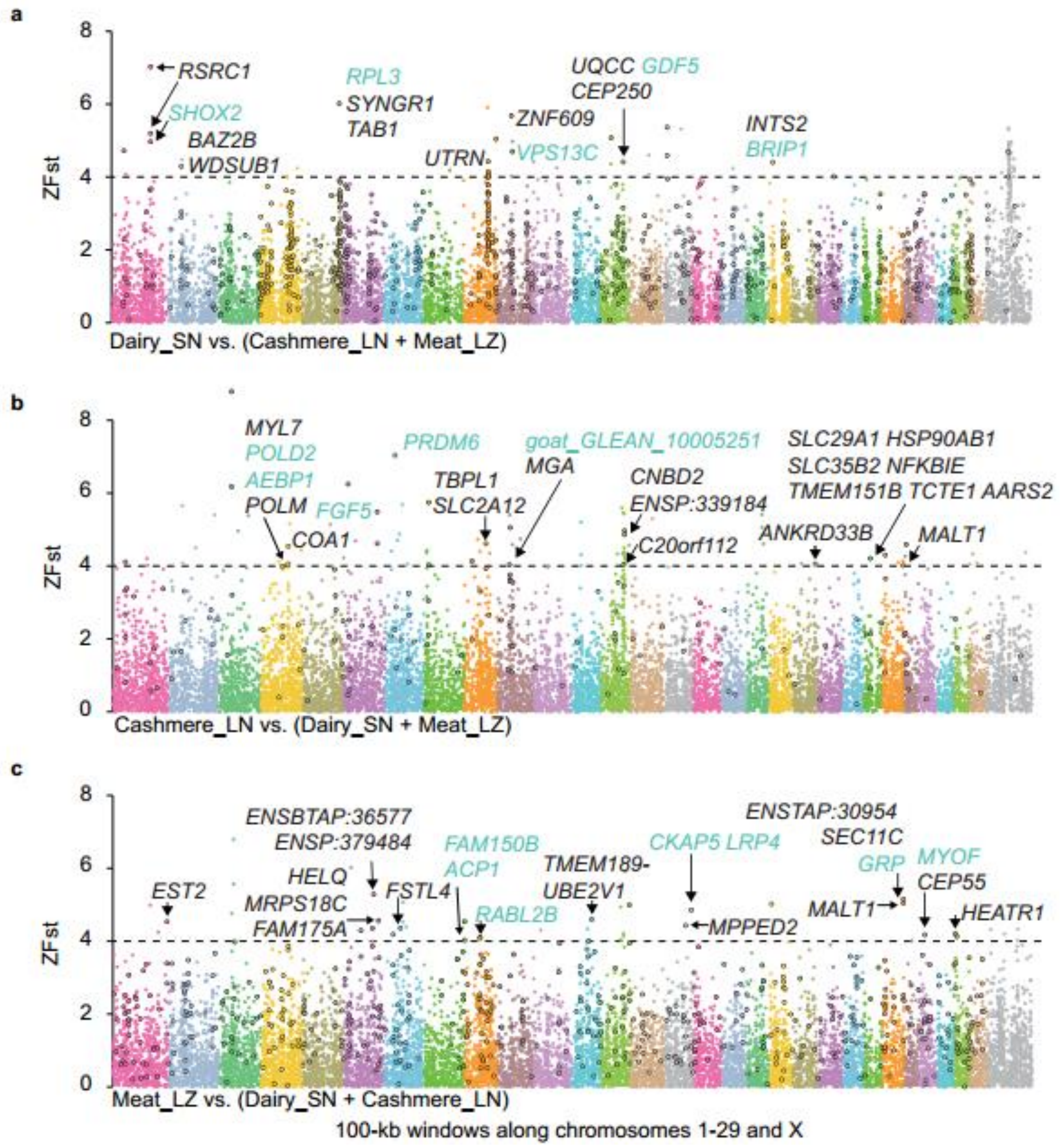

Figure 4 Candidate selective sweep analysis of each economic breed. Selective sweeps and their associated genes are showed in the three breeds: **(a)** Saanen breed, **(b)** Liaoning Cashmere breed, and **(c)** Leizhou breed. The windows passed the threshold ZFst >4 and $P<0.05$ in HKA test are extracted as selective sweeps. TAGs labeled in color are discussed in the text.

Figure Click here to download Figure Figure 5.docx

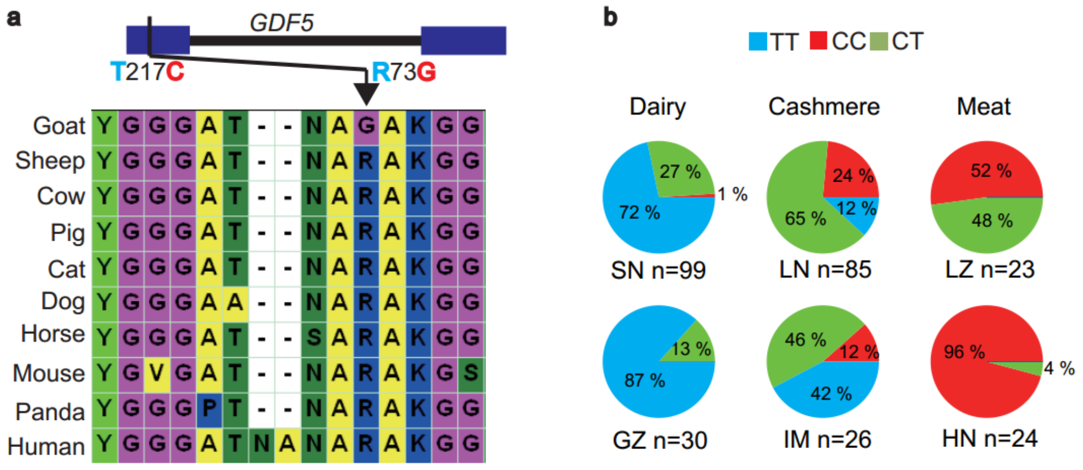

Figure 5 Opposite selection for sites in *GDF5* of the dairy and meat breeds. (a) A nonsysnonmous SNP T217C (R73G) is located in the first exon of *GDF5*. The amino acids at this position are highly conserved in other mammals. **(b)** Frequency divergence happened in different economically relevant traits. Allele T is dominant in the dairy breed (Saanen and Guanzhong), while C is dominant in the meat breed (Leizhou and Hainan). SN: Saanen goat, LN: Liaoning Cashmere goat, LZ: Leizhou goat, GZ: Guanzhong goat, IM: Inner Mongolia Cashmere goat, HN: Hainan goat.

Click here to access/download
Supplementary Material
Goat_Supplnote.doc
