# Supplementary material for: Genome-wide definition of selective sweeps reveals molecular evidence of trait-driven domestication among elite goat (Capra species) breeds for the production of dairy, cashmere, and meat
Source: Gigascience. 2018 Aug 27;7(12):giy105. doi: 10.1093/gigascience/giy105 (PMC6287099; doi:10.1093/gigascience/giy105)
Supplement: GIGA-D-17-00226_Revision_1.pdf [file giy105_giga-d-17-00226_revision_1.pdf]

GigaScience

Genome-wide definition of selective sweeps reveals molecular evidence of trait-driven domestication among elite goat (Capra species) breeds for the production of dairy, cashmere, and meat

--Manuscript Draft--

Manuscript Number:	GIGA-D-17-00226R1
Full Title:	Genome-wide definition of selective sweeps reveals molecular evidence of trait-driven domestication among elite goat (Capra species) breeds for the production of dairy, cashmere, and meat
Article Type:	Research
Funding Information:	National Natural Science Foundation of China (CN) (31301949) — Dr. bao zhang<br>National Natural Science Foundation of China (CN) (31272408) — Not applicable<br>National Natural Science Foundation of China (CN) (31172184) — Not applicable<br>National Science Foundation for Post-doctoral Scientists of China (2013M532056) — Dr. bao zhang<br>Research Fund for the Doctor Program of Higher Education of China (20120204110007) — Dr. bao zhang
Abstract:	Domestication of wild goats and subsequent intensive trait-driven crossing, inbreeding, and selection, all have led to dramatic phenotypic purification and intermediate breeds for high-quality production of dairy, cashmere, and meat. Genomic re-sequencing provides a powerful mean for direct identification of trait-associated sequence variations that underlie molecular mechanisms of domestication. Here, we report our effort to define such variations based on data from domestic goat breeds (Capra eagagrus hircus; 5 each) selected for dairy, cashmere, and meat production in referencing to their wild ancestors, Sindh ibex (Capra eagagrus blythi; 2) and Markhor (Capra falconeri; 2). Using ~24 million high-quality single nucleotide polymorphisms (SNPs), ~1.9 million insertions/deletions (indels), and 2,317 copy number variations (CNVs), we define SNP-desert-associated genes (SAGs), domestic-associated genes (DAGs), and trait-associated genes (TAGs), and attempt to associate them with quantitative trait loci (QTL), domestication, and agronomic traits. Surprisingly, a greater majority of SAGs shared by all domestic breeds classified into Gene Ontology categories of metabolism and cell cycle. DAGs, together with some SAGs, are most relevant to behavior, immunity, and trait-specificity, whereas TAGs appear directly involved in growth regulation, such as growth differentiation factor 5 (GDF5) and fibroblast growth factor 5 (FGF5) for bone and hair growth, respectively. When investigating divergence of Capra populations, the sequence variations and candidate genes we have identified provide valuable molecular markers for trait-driven genetic mapping and breeding.
Corresponding Author:	bin sheng li<br>Xi'an Jiaotong University<br>Xi'an, CHINA
Corresponding Author Secondary Information:	
Corresponding Author's Institution:	Xi'an Jiaotong University
Corresponding Author's Secondary Institution:	
First Author:	bao zhang
First Author Secondary Information:	

Powered by Editorial Manager® and ProduXion Manager® from Aries Systems Corporation

**Order of Authors:**	bao zhang
	Liao Chang
	Yong Xian Lan
	Nadeem Asif
	Fang Lin Guan
	Ke Dong Fu
	Bo Li
	Xia Chun Yan
	Bo Hong Zhang
	Yan Xiao Zhang
	Zhen Yong Huang
	Hong Chen
	Jun Yu
	Bin Sheng Li
**Order of Authors Secondary Information:**	
**Response to Reviewers:**	Dear Dr. Hans Zauner<br>Thank you very much for handling our manuscript "Genome-wide definition of selective sweeps reveals molecular evidence of trait-driven domestication among elite goat (Capra species) breeds for the production of dairy, cashmere, and meat" (GIGA-D-17-00226). We appreciate all comments from the reviewers, which are extremely helpful for us to improve our manuscript. We have now revised our manuscript accordingly as highlighted in colored and quotation-marked. Because of additional figures, we attach a list of current figures and tables, which are referenced to the last version so that the revision can be readily tracked. We list all responses as follows:<br>From editor:<br>1.The section has been extensively rewritten.<br>2.The language has been professionally edited by an English editing service agency, Springer Nature Language Editing.<br><br>************************<br>From REVIEWERS<br>************************<br>Reviewer #1:<br><br><br>In this manuscript the authors have identified several polymorphisms in domestic goat breeds that could be linked to their domestication process.<br>The authors have also shown that these polymorphisms could be good molecular markers for genetic mapping and breeding.<br>These findings are interesting and the authors have presented some novel data. However, there are some major issues that need to be addressed. In addition, in its present state the overall report is difficult to follow for the English reader and requires editing.<br><br>Response: Thanks for your positive comments and encouragement. We have substantially revised the manuscript, and the manuscript has been professionally edited by an English editing service agency, Springer Nature Language Editing.<br><br>* Are the conclusions adequately supported by the data shown? No<br>- Poor discussion. It does not allow to link the results found with the conclusions about the biological meaning. I suggest the authors to develop more the discussion part, to make clearer to the reader the impact of the findings.<br>- Poor conclusions<br><br>Response: We have substantially revised the manuscript to emphasize major points.

* Does the manuscript adhere to the journal's guidelines on minimum standards of reporting? No
- Organisms: report source, species, strain, sex, age, husbandry, inbred and strain characteristics of transgenic and mutant animals --> Lack of information in the materials and methods section

Response: We add the information (source, species, sex) in the Materials and Methods section (Line 329-331). And some more details are added as supplementary methods 4.1.

- Availability of Data and Materials --> Have the SNPs found (T217C for instance) being submitted to the genebank dbSNP and publicly available? If so, please add the reference

Response: We did not find goat SNP collection at Genebank. We submitted our goat SNP data to EBI. Due to the journal policy, we will release the accession numbers as the genome sequence data release at NCBI.

Comments:

- Line 37 "Surprisingly": I would just expect that, isn't it?. A breed selected for production traits, would have less SNP content in the genes of interest for which have been selected.

Response: We also think there should be less SNP content in the gene of interest for which have been selected in the trait-selected breeds. We deleted the word “Surprisingly” in line37.

- Lines 112-113: "...indicating recent introduction of genetic heterogeneity...": Do you have data sustaining this assumption? References?

Response: The Saanen breed is a famous dairy breed from Switzerland. It has been introduced to many parts of the world, giving rise to numerous local hybrids. For instance, it was brought to China in 1920s.

[1] Jean-Paul Dubeuf. An international panorama of goat selection and breeds. Livestock Science, 2009, 120(3): 225-231.
[2] Breeds of domestic animal and poultry in China, sheep and goat breeds in China writing group, Sheep and goat breeds in China.1988. (In Chinese).

- Lines 111-114 "the dairy breed appears to have slightly more unique SNPs," and Supplementary figure 7: In this figure in fact, dairy breed and cashmere breed seem to have similar number of SNP, however, meat breed shows a really lower number of SNPs compared to the other two. What do you think that could be due to?

Response: We are thinking of two obvious factors. First, as we show in Figure 1, the 5 representative individuals of the meat breed exhibit closer relationship that leaves less within-breed polymorphisms as compared to the other two breeds. Second, the Leizhou breed itself is also known to be heavily inbreeded in its recent history, and such practice may lead to an effect of purifying selections.

- A high number of polymorphisms shared between dairy and cashmere in general. Cashmere breeds in fact, usually have a dual-purpose: dairy and Is it the case of this breed? Does the LN cashmere breed come from dairy breeds? Does by chance could come from any of the dairy breeds included in this work? That will explain these results and similarities.

Response: Liaoning Cashmere is a famous cashmere breed in China. Its within-breed trait selection began about 1980s. Before this selection for cashmere, it might be actually raised for two purpose, cashmere and milk.

[1] Suggestion for breed selection of Liaoning cashmere goat. Zhao QT, Modern Journal of Animal Husbandry and Veterinary Medicine 1981, 21-23. (In Chinese).

Powered by Editorial Manager® and ProduXion Manager® from Aries Systems Corporation

- Paragraph from lines 161-170: It should be rewritten, I don't think what it is explained is clear.

Response: (lines 164-172), We used principal component analysis (PCA) and phylogeny reconstruction to evaluate the population structure of the domestic breeds. First, based on our genome-wide SNP data, we found that Sindh ibex is genetically closer to the domestic breeds as compared to Markhor, consistent with previous reports. Second, our PCA result suggests that the domestic breeds and the wild breeds are both distant and distinct (Fig. 1a), where, as the neighbor-joining tree shows, the two Chinese domestic breeds are closer to each other and the Saanen breed is closer to the wild goats than the other two domestic breeds. Third, all results collectively suggest that the domestication traits for dairy production may occur ahead of cashmere and meat in goat domestication (Fig. 1).

- Figure 1B "the Saanen breed is rather close to the wild goats" Not clear in the figure, maybe trying to make a zoom of this part of the branch?

Response: Both Figure1a (component 2) and 1b suggest this point although in 1b the distance is very subtle.

- Lines 168-170 "all results suggest that the domestication traits of dairy production may occur ahead of cashmere and meat in the domestication history of goats": There are already references suggesting the fact that goats could primarily have be domesticated for dairy purposes, so you should include them.

Response: Yes. We added the following reference to the manuscript (Line 172).

[1] Jean-Paul Dubeuf. An international panorama of goat selection and breeds. Livestock Science, 2009, 120(3): 225-231.

- Section TAGs in the dairy breed: Various TAGs, however, the authors does not explain how and why this could be due to. It needs more concretion.

Response: It is true that the dairy breeds have more TAGs due to more SNP desert but we do not have idea why but suggest that it is historic on one hand and function-related on the other. We did not speculate much as the dataset is not big enough for further scrutiny.

- T217C SNP: Lines 268-272: The SNP seems to only exist in domestic goats, what about in wild goats? Why do you think the explanation of this SNP is due to the largest body of dairy goats when it is in this breed where the most frequent allele is T as in concordance with the rest of the species analyzed? Shouldn't you look for an explanation in the meat breeds where the presence of C is dominant?

Response: We revised the manuscript and attempt to explain the points.
In Figure 5, we compared the difference of the GDF5 T217C locus in the two meat breeds (Leizhou and Hainan) and the 4 wild goats, where the C allele is dominant in the domestic meat breeds and the T allele is dominant in the wild goats. Since the T allele is also seen in the dairy goats, we suggest that T is ancestral where as C is selected. In addition, we have looked into the breeding history of Leizhou and learnt that the body size has been smaller than Sannen and Liaoning cashmere goats. GDF5 is a member of the TGF-beta superfamily, which is involved in height [1] and multiple skeletal structures [2].

[1] S Sanna, Jackson, A U, Nagaraja, R, et al. Common variants in the GDF5-UQCC region are associated with variation in human height. Nature genetics 2008; 40: 198-203.
[2] Settle SH Jr, Rountree RB, Sinha A, Thacker A, Higgins K, Kingsley DM. Multiple joint and skeletal patterning defects caused by single and double mutations in the mouse Gdf6 and Gdf5 genes. Dev Biol. 2003 Feb 1; 254(1):116-30.

- QTL mapping: What about taking QTL data also from cows? Wouldn't it be more

informative?

Response: We used the cattle information that includes cow data. And there has not been independent QTL dataset for cow.

- I find the presence of SNP deserts really interesting. But I find a lack of further explanation. Why do you think there is a SNP desert along the RSRC1 sequence (Fig.2)? What could this gene have as special? Could you take a look to the wild species or to other species to compare this phenomenon?

Response: For defining SNP desert, we randomly choose one gene (RSRC1) as an example. There are a lot of SNP desert genes in our dataset (Supplementary tables 11, 12, 13). We added data from wild goat, human, and cheetah to illustrate this phenomenon. it is clear that the domestic goat breeds have lower SNP rates in the RSRC1 gene locus when compared to the wild goat and other species (Supplementary Figure 16).

Figures:
- Figure 1a: Please add the % of the variability explained in the PCA graph.
- Figure 1b: For me was not that clear to see in the neighbor tree the branch were dairy and wild breeds are together, maybe a zoom of the figure?

Response: We added the percentage of SNPs that contribute to the distribution in PCA graph.

English correction is needed. Some examples:
- Line 38: "breeds are classified into Gene Ontology categories"
- Line 40: " trait-specificity. Whereas..."
- Line 50-51: "crucial factors were selected including docility.."
- Line 58: "dramatically around 200 years ago..."
- Line 58-59: "Selection increased intensively.."
- Line 60: " among breed was seriously reduced"
- Line 76: " it is still lagging..."
- Sentence in lines 99-100 "In addition, validated our SNP..". It needs to be re-written
- Sentence in lines 211-215. It needs to be re-written
- Sentence in lines 315-317 "To validate them in a larger population..." It needs to be re-written, I don't understand the sentence

Response: We have revised the manuscript extensively, and the manuscript has been professionally edited by an English editing service agency (Springer Nature Language Editing).

Others:
- Line 77: " cattle , pig , dog , and chicken ." Remove space between words and punctuations/grammatical marks.

Response: We extensively proofread the entire manuscript accordingly.

Reviewer #2: Main: line 388: HKA is a method to detect signatures of selection for macroevolution, therefore related to selection between species (Vitti et al., 2013). Therefore, it is not suited to compare populations within a species, but you can apply it to the previous comparison (wild vs domestic). In its place, I would suggest the application of methods such as LKT (Lewontin-Krakauer Test), FLK, XP-EHH or XP-CLR, which have results comparable with Fst.

Response: We have re-analyzed this part with XP-EHH and revised the manuscript accordingly (lines 236-239 and lines 394-396).

lines 336-341: What is the reads length produced from the sequencer?

Response: The average raw reads length is 150 bp. We have added this information in Methods (line 340).

line 343: Why use such an old version of BWA (v 0.5.9, released in 2011) when the

	more "modern" 0.7.12 is available since 2014?<br><br>Response: We acquired the raw data in 2012, so BWA 0.5.9 was used. We tested the alignment of 5 samples using BWA 0.7.12, and the genotype calls between the two versions are 99% identical. We have also randomly selected 160 SNPs and validated them with capture-NGS-based genotyping method (Genesky Biotechnologies, Shanghai, China), and the accuracy is 97.43%.<br><br>line 344: Why use the aln alignment algorithm instead of mem?<br><br>Response: BWA-MEM is faster and more accurate in long-read alignment. For Illumina sequence reads, there are high error rate at the ends of sequence reads. BWA-backtrack algorithm (aln/sampe) trims low-quality bases from the 3'-end before alignment. Our data is generated with the Illumina platform, so we choose BWA-backtrack algorithm (aln/sampe) for sequence quality control.<br><br>[1] http://bio-bwa.sourceforge.net/<br>[2] Li H. and Durbin R. (2009) Fast and accurate short read alignment with Burrows-Wheeler Transform. Bioinformatics, 25:1754-60.<br><br>lines 357-358: You call a SNP if you have at least 4 reads per allele, but only 2 reads for indels: why this difference?<br><br>Response: SNP calls correlate to the sequence error rate in the Illumina platform, where the quality of base calling decreases when the accuracy of SNP calling is lower. The correlation between indel quality and base calling errors is relatively poorer. In other words, miscalled indels are rarer than SNPs.<br><br>lines 366-370: please add references for PCA (EIGENSOFT), FRAPPE and PHYLIP software.<br><br>Response: We added two references in Methods (line375 and line 377). In this version, we eliminated FRAPPE.<br><br>[1] PCA (EIGENSOFT) N Patterson, Price, A L, and Reich, D. Population structure and eigenanalysis. PLoS Genet 2006; 2: e190.<br>[2] PHYLIP J D Retief. Phylogenetic analysis using PHYLIP. Methods Mol Biol 2000; 132: 243-58.<br><br>lines 371-377: does the SNP deserts regions considers the indels? If not, why did you excluded them? in addition, it could be interesting to compare the SNP deserts regions with some well-established method to detect low diversity regions (e.g. ROH)<br><br>Response: We did not include indels when screening SNP deserts. The number of SNPs is 10 times more than indels so they have little effect on SNP deserts. ROH method is based on allele frequencies. In our small population, the distribution of allele frequency may differ when a large population is used. Therefore, we use the number of SNPs in a window rather than frequency itself.<br><br>line 379: Is Hp the pooled heterozygosity cited by Bahbahani et al. (2017; DOI: 10.3389/fgene.2017.00068)? If so, it should be cited properly, referring to some works that compare different species, and defining what kind of selection it identifies.<br><br>Response: Hp, pooled heterozygosity. We added a reference (Result line 387). Hp is used to detect putative selective sweeps during domestication(DAG).<br><br>[1] CJ Rubin, Zody MC, Eriksson J, Meadows JR, Sherwood E, Webster MT, Jiang L, Ingman M, Sharpe T, Ka S, et al. "Whole-genome resequencing reveals loci under selection during chicken domestication," Nature, vol. 464, no.7, pp. 587-591, 2010.
**Additional Information:**	
**Question**	**Response**

Are you submitting this manuscript to a special series or article collection?	No
**Experimental design and statistics**<br><br>Full details of the experimental design and statistical methods used should be given in the Methods section, as detailed in our Minimum Standards Reporting Checklist. Information essential to interpreting the data presented should be made available in the figure legends.<br><br>Have you included all the information requested in your manuscript?	Yes
**Resources**<br><br>A description of all resources used, including antibodies, cell lines, animals and software tools, with enough information to allow them to be uniquely identified, should be included in the Methods section. Authors are strongly encouraged to cite Research Resource Identifiers (RRIDs) for antibodies, model organisms and tools, where possible.<br><br>Have you included the information requested as detailed in our Minimum Standards Reporting Checklist?	Yes
**Availability of data and materials**<br><br>All datasets and code on which the conclusions of the paper rely must be either included in your submission or deposited in publicly available repositories (where available and ethically appropriate), referencing such data using a unique identifier in the references and in the “Availability of Data and Materials” section of your manuscript.<br><br>Have you have met the above requirement as detailed in our Minimum	Yes

Standards Reporting Checklist?	

Powered by Editorial Manager® and ProduXion Manager® from Aries Systems Corporation

Manuscript
Click here to access/download;Manuscript;Goat-article-16_8_18.docx
Click here to view linked References

Genome-wide definition of selective sweeps reveals molecular evidence of trait-driven domestication among elite goat (*Capra* species) breeds for the production of dairy, cashmere, and meat

Zhang Bao[1+], Chang Liao[1+], Lan Xianyong[2+], Asif Nadeem[3], Guan Fanglin[1], Fu Dongke[1], Li Bo[1], Yan Chunxia[1], Zhang Hongbo[1], Zhang Xiaoyan[2], Huang Yongzhen[2], Chen Hong[2], Yu Jun[4*], Li Shengbin[1*]

1. College of Medicine & Forensic, Health Science Center, Xi'an Jiaotong University, Xi'an, Shaanxi, People's Republic of China;
2. Northwest A&F University, Shaanxi Key Laboratory of Molecular Biology for Agriculture, Yangling, Shaanxi, People's Republic of China;
3. Institute of Biochemistry and Biotechnology, University of Veterinary and Animal Sciences, Lahore, Pakistan;
4. CAS Key Laboratory of Beijing Institute of Genomics, Chinese Academy of Sciences, Beijing, People's Republic of China.

+These authors contributed equally to this work

*co-corresponding authors, correspondence and requests for materials should be addressed to: shbinlee@mail.xjtu.edu.cn; junyu@big.ac.cn

Abstract

Background: The domestication of wild goats and subsequent intensive trait-driven crossing, inbreeding, and selection have led to dramatic phenotypic purification and intermediate breeds for the high-quality production of dairy, cashmere, and meat. Genomic re-sequencing provides a powerful means for the direct identification of trait-associated sequence variations that underlie molecular mechanisms of domestication.

Results: Here, we report our effort to define such variations based on data from domestic goat breeds (*Capra aegagrus hircus*; 5 each) selected for dairy, cashmere, and meat production in referencing to their wild ancestors, the Sindh ibex (*Capra aegagrus blythi*; 2) and the Markhor (*Capra falconeri*; 2). Using ~24 million high-quality single nucleotide polymorphisms (SNPs), ~1.9 million insertions/deletions (indels), and 2,317 copy number variations (CNVs), we define SNP-desert-associated genes (SAGs), domestic-associated genes (DAGs), and trait-associated genes (TAGs) and attempt to associate them with quantitative trait loci (QTL), domestication, and agronomic traits. A greater majority of SAGs shared by all domestic breeds are classified into Gene Ontology categories of metabolism and cell cycle. DAGs, together with some SAGs, are most relevant to behavior, immunity, and trait-specificity. Whereas, TAGs, such as growth differentiation factor 5 (*GDF5*) and fibroblast growth factor 5 (*FGF5*) for bone and hair growth, respectively, appear to be directly involved in growth regulation.

Conclusions: When investigating the divergence of *Capra* populations, the sequence variations and candidate function-associated genes we have identified provide valuable molecular markers for trait-driven genetic mapping and breeding.

Keywords: Goat; re-sequencing; trait-driven domestication

Background

As one of the most popular farm mammals, goats (*Capra hircus*) were domesticated ~10,000 years ago [1]. In early domestication, crucial factors were selected including docility toward humans and loss of wild-type behavioral characteristics [2]. Following the initial domestication events for crops in the Fertile Crescent, together with culture diffusion over Europe, Africa, and Asia, animal domestication had spread rapidly as an integral part of the Neolithic Revolution [3] . Once farming developed in the Middle East and Asia in ~7,000 B.C., human settlements became permanent and domesticated animals assured a better supply of food and clothing [4]. After a long period of so-called *soft* selection, the situation changed dramatically around 200 years ago with the emergence of the *breed* concept [3]. Selection increased intensively in local populations, followed by standardization of trait performance, and reproductive breeding among breeds was seriously reduced, leading to fragmentation of the initial gene pools. More recently, selection pressure has increased again via the use of artificial insemination, resulting in a few industrial breeds with high trait performance, low effective population size, and profound phenotypic changes [5], such as the case of trait-driven breeding for dairy, cashmere, and meat [6].

Goats number ~800 million in population and in ~560 breeds (12 percent of the total recorded mammalian breeds). They are the most adaptable livestock on all continents [7] and supply milk, meat, and fiber for human consumption, and while thriving on meager fodder and in harsh environments [8]. Despite the importance of this species, the study of goat genomes is still in its infancy compared to that of other farm animals [9]. Nevertheless, positional cloning has demonstrated that the polled intersex syndrome (PIS) is located on 1q43 of the goat genome [10]; transcriptomic studies have paved the way for in-depth genomics, including various trait-relevant tissues, such as mammary glands, skeletal muscle, and hair follicles; some genetic

studies have also been performed on traits and disease resistance [11]. Although genome-wide studies of goat QTL and genome sequences have advanced the field [12, 13], it is still lagging behind those of other domestic animals, such as cattle, pig, dog, and chicken.

Our experimental design involves the resequencing (~29× in sequencing depth and 99% in genome coverage) of 15 domestic goats representing three breeds and 4 wild goats from 2 distinct species. The high-quality sequence data allow us to use high-quality genetic markers (single nucleotide polymorphism, SNP; insertion/deletion, indel; and copy number variation, CNV) to define artificial selection related genes in the history of goat domestication. In particular, studies of trait-associated genes (TAGs) provide candidate loci for marker-assisted breeding of domestic goats.

Results and Discussion

Sequence variation identified in five goat groups

We sequenced three elite domestic goat breeds (5 each), including dairy (Saanen), cashmere (Liaoning cashmere), and meat (Leizhou), and two wild goat species (Sindh ibex, *Capra aegagrus blythi* and Markhor, *Capra falconeri*; 2 each) as controls (Supplementary Fig. 1). Both the Sindh ibex and Markhor are Pakistan wild goats; the latter categorized as endangered on the IUCN Red List (Supplementary Fig. 2). We generated 1,346 Gb (28.8×) and 379 Gb (28.6×) raw data for the domestic and wild goats, respectively (Table 1; Supplementary Table 1 and Supplementary Fig. 3). Referenced to the *Capra hircus* genome (GenBank Accession: GCA_00317765.1), we identified 23,924,294 SNPs, 1,899,827 indels, and 2,317 CNVs.

Single nucleotide polymorphisms (SNPs)

We analyzed the high-quality SNPs with a criterion of a minimum depth ≥ 8 in every individual sample (Supplementary Fig. 4). In addition, we validated the SNP calling accuracy rate (97.43%) using a sequence-capture NGS-based genotyping method (Genesky Biotechnologies, Shanghai, China; Supplementary Note).

First, the SNPs were partitioned into intergenic (76.20%), intronic (23.06%), and protein-coding (0.74%) SNPs, and subsequently, the ratio of nonsynonymous to synonymous substitutions (NS/S) was calculated as 0.95 on average. However, the NS/S ratio shows variable distributions when correlated to minor allele frequency (MAF) in the low SNP rate region or the SNP desert (Supplementary Table 2; Supplementary Figs. 5 and 6). Second, we identified millions of SNPs within and between the wild and domestic goat groups. Although the number of wild-goat-specific SNPs is smaller than that of the domestic group (5,598,396 vs. 12,434,312 and 6,061,698 shared), this result may reflect biased sampling (15 vs. 4) rather than true genetic heterogeneity in the groups. Third, among the SNPs unique to each domestic breed, the dairy breed appears to have slightly more unique SNPs, indicating the recent introduction of genetic heterogeneity [14, 15], as opposed to the cashmere breed, which appears to have more in total when breed-shared SNPs are considered (Supplementary Fig. 7). At low MAFs, there are a higher proportion of breed-specific SNPs than the total, but there is a transition at MAF 20%, where the breed-specific SNP proportion becomes obviously less than the total (Supplementary Fig. 8). In addition, the meat

breed has more ancient SNPs with higher MAFs than the other two breeds, whereas the cashmere breed is relatively young or less selected as it has more low frequency SNPs (Supplementary Fig. 8). Fourth, we compared heterozygous SNPs across all chromosomes and found that the meat breed has significantly lower heterozygosity (P= 0.0022) than the two other breeds, suggesting that there may be strong or long-term selection during its breeding (Supplementary Table 3).

Insertions/deletions (indels)

We categorized 1,899,827 indels with nearly equal numbers of insertions and deletions, of which ~0.13% (2,420) were found in protein-coding sequences and partitioned into 32.72% (792) in-frame (3-bp indels) and 1,628 out-of-frame indels that lead to an average of 499 pseudogenes per individual sample. Similar to the trend observed for SNPs, there are much rarer indels in the total (Supplementary Figs. 9 and 10); we observed more indels that were domestication-specific than wild-specific (924,352 vs. 363,589), and more indels in the dairy and cashmere breeds compared to the meat breed (Supplementary Fig. 11 and Supplementary Table 4).

In addition, based on our indel data, *AADAC* (arylacetamide deacetylase) appears to selected in the dairy breeds, encoding an enzyme responsible for the hydrolysis of drugs [16]. The *MYT1L* (myelin transcription factor 1-like) marker associated with syndromic intellectual disability and early-onset obesity has shown a meat-specific high frequency indel frame-shift polymorphism [17] (Supplementary Table 5).

Copy number variation (CNV)

In the 1-kb window, there are 2,028 (246 genes; spanning a 32.0-Mb genomic region) and 1,616 CNVs (144 genes; spanning a 19.2-Mb genomic region) in the domestic and wild goats, respectively. The wild goats and the meat breeds have relatively higher numbers of CNVs than their domestic counterparts and two other domestic breeds (Supplementary Table 6). The meat goats have more breed-specific CNVs compared to dairy and cashmere breeds (38 vs. 33 and 22) and 11 CNVs were shared by all three breeds (Supplementary Fig. 12).

Interestingly, consistent with a previous study [18], we observed high frequency domestication-specific CNVs in the region including *ASIP* and *AHCY* genes which are related to skin pigmentation and coat color in sheep [19]. Goats with white hairs (Saanen and Liaoning cashmere goats) have many more copies of *ASIP* and *AHCY* than those goats with colored hairs (Leizhou and wild goats; Supplementary Table 7). This result also confirmed in our larger population sampling with a white and black coat population (n= 54; Supplementary Fig. 13). Thus, *ASIP*-*AHCY* region is a domestication locus in goat and may be select for coat color.

Finally, to validate whether CNV loci were associated with dairy traits, 12 candidates dairy specific CNV loci were detected in 130 Guanzhong dairy goat using AccuCopy assay (Genesky Biotechnologies, Shanghai, China). Our CNV association study points to two CNV loci (including *APOL3* and *NEM6*; $P<0.01$) for dairy and growth traits

(Supplementary Table 8). *APOL3* (apolipoprotein L3) is a lipid transport and metabolism associated gene that is also highly duplicated in beef breeds [20], and *NME6* (NME/NM23 nucleoside diphosphate kinase 6) is suggested to play a role in cell growth and the cell cycle [21]. This study indicates that duplication of the *APOL3* locus conferred a selective advantage to dairy, while there is a negative correlation between the duplication of *NME6* and growth, and need further investigation in dairy goat.

Population structures of domestic and wild goats

We used principal component analysis (PCA) and phylogeny reconstruction to evaluate the population structure of the domestic breeds. First, based on our genome-wide SNP data, we found that Sindh ibex is genetically closer to the domestic breeds as compared to Markhor, consistent with previous reports [22]. Second, our PCA result suggests that the domestic breeds and the wild breeds are both distant and distinct (Fig. 1a), where, as the neighbor-joining tree shows, the two Chinese domestic breeds are closer to each other and the Saanen breed is closer to the wild goats than the other two domestic breeds. Third, all results collectively suggest that the domestication traits for dairy production may occur ahead of cashmere and meat in goat domestication [15] (Fig. 1).

SNP-desert-associated genes (SAGs)

SNP deserts are often linked to beneficial mutations as selective sweeps that are subjected to strong purifying selection [23]. The SNP deserts are defined as genomic regions with lowest 10% SNP rates (10-kb windows). SNP-desert-associated genes (SAGs) are selected if they are harbored by SNP deserts (>30%; Fig. 2). We noticed that there is a bimodal SNP rate only distribution in the dairy and meat breeds; the large

absence of SNP-poor regions suggests the effect of both stronger recent purifying selection and a lack of recent introduction of genetic heterogeneity in the cashmere breed compared to the two domestic breeds. In addition, the lower mean and median SNP rates of the meat breed (Fig. 2a and Supplementary Fig. 14) suggest overall poorer genetic heterogeneity or heavier inbreeding. In total, 277.39 Mb (3,950 SAGs), 278.33 Mb (4,395 SAGs), and 273 Mb (3,447 SAGs) SNP deserts were detected for the dairy, cashmere, and meat goat genomes, respectively (Fig. 2b and Supplementary Table 9). For the 1,196 SAGs shared among the domestic breeds, Gene Ontology (GO) enrichment shows only two major categories: metabolism and cell cycle regulation (Supplementary Fig. 15a and Supplementary Table 10).

To further investigate, we also examined the large SNP deserts (>100 kb in length) as well as the top 10 larger deserts unique to each domestic breed. For the SNP deserts >100 kb in length, it is consistent that the dairy (1,112) and cashmere (1,503) breeds had more SAGs than the meat breed (1,044); the function of the 231 breed-shared SAGs appears to be related to signal transduction (such as *RSRC1*, Fig. 2c and Supplementary Figs. 15b and 16). To provide alternative insights, we scrutinized the top 10 SNP deserts in three breeds. When looking for breed-shared SAGs in the top 10, we observed only one SNP desert, including *AR* gene on chr X. *AR* (androgen receptor) is a hormone-inducible DNA-binding transcription factor, that plays an essential role in male reproduction; its knock-out male mice display severely impaired reproductive tracts and sexual behavior [24], which indicate male reproduction may have been an important evolutionary force during goat domestication. For the top 10 SNP deserts found in each breed, the meat breed has two unique loci, and the dairy breeds have four, but none for the cashmere breed (Supplementary Discussion, Supplementary Table 11, 12, 13).

Domestication-associated genes (DAGs)

To detect the sequence signature of selective sweeps over large genomic regions, we first calculated the pooled heterozygosity (Hp), using autosomal SNPs from all individuals of the domestic breeds in a 100-kb sliding window. We also calculated the fixation index (Fst), which indicates population differentiation between domestic and wild populations in a 100-kb sliding window based on autosomal SNPs. We then transformed the Hp and Fst into Z (Hp) and Z (Fst), respectively, and the protocol defined 67 domestication-associated genes (DAGs) in a collective genomic length of 3.2 Mb (Fig. 3). The 67 DAGs are all overlapped with SAGs in one, two, or three goat breeds (Supplementary Fig. 17 and Supplementary Table 14). Our GO enrichment analysis indicates that the significant categories (FDR $q<0.001$) are negative regulation of gene expression, protein import into the nucleus, and docking (Supplementary Fig. 18 and Supplementary Table 15).

This set of DAGs genes may contribute to behavioral, immune, and morphological differences between domestic and wild goats. First, genes that directly influence the nervous system and behavior include *ADRA2A* (alpha-2-adrenergic receptors, which regulate neurotransmitter release), and *FXR2* (fragile X mental retardation, autosomal homolog 2, which required for the presence of behavioral circadian rhythms) [25, 26]. Second, *TNFSF13* (tumor necrosis factor (ligand) superfamily, member 13) and *STIM1* (stromal interaction molecule 1) are located in the region associated with cattle body weight gain [27, 28] and regulation of B-cell development and T cell-mediated immune regulation during chronic infection [29, 30], respectively. Third, the morphological difference involves genes: *NR6A1* (Nuclear Receptor Subfamily 6 Group A Member 1), which affects the number of vertebra, one of the most characteristic morphological changes in domestic pigs [31], and *STAT6* (signal transducer and activator of

transcription 6), which is associated with body weight as well as carcass and growth efficiency traits [32] (Supplementary Discussion). These findings support the hypothesis that selection for altered nervous system and behavior for docility, amended immune and morphological for production efficiency were important during domestication and these changes were reflected by mutation of genes.

Trait-associated genes (TAGs) of domestic breeds

To uncover genetic variants involved in local adaptation and selection in the three breeds, we performed Fst and cross-population extended haplotype homozygosity (XP-EHH) in a 100-kb window on SNPs from one breed against a pool of the two other breeds. Using the criterion of Fst>4 and a top 1% outlier of XP-EHH, we defined 54 trait-associated genes (TAGs) (Supplementary Table 16).

To further explore artificial selection-related genes in the TAGs, two hundred SNP genotype frequencies at non-synonymous sites within TAGs were detected using 287 individuals representing 7 populations in China. First, consistent with 19 sequencing individuals, the *GDF5* (growth differentiation factor 5) T217C (amino acid changed: R73G) locus of meat breeds (Leizhou and Hainan) is dominant with C allele and the T allele is dominant in the dairy, cashmere and wild goats. As R is conserved among all other known mammal sequences except goat, we suggest that T is ancestral whereas C is selected. In addition, we have looked into the breeding history of Leizhou and learnt that the body size is smaller than Sannen and Liaoning cashmere goats. *GDF5* is a member of the TGF-beta superfamily, which is involved in height [33] and multiple skeletal structures [34] (Fig. 5 and Supplementary Fig. 2). Second, *LRP4* (low density lipoprotein receptor-related protein 4) is detected with a conserved amino acid change (TT at 266th nucleotide) in goats, and it also showed opposite selection in the meat and other goat breeds (Supplementary Fig. 19). A functionally related candidate gene

affecting bone-mass homeostasis and with a central role for high bone mass, syndactyly, sclerostin receptor [35].

The TAGs are cross-referenced with data on co-localization with cow and sheep QTL and SAGs (Fig. 4) [36]. A striking correlation was detected between putative selective sweeps and SNP-deserts; there are 660, 912, and 1,841 genes shared by SAGs and QTLs unique to the dairy, cashmere, and meat breeds, respectively (Supplementary Fig. 20). Most of these genes were enriched in GO categories of metabolic process, biological regulation, and response to stimulus, whereas the trait-specific categories include reproduction and growth (Supplementary Fig. 21). Therefore, our results illustrate the important role of biological pathways influencing growth in the goat.

TAGs in the dairy breed

There is no gene shared by TAGs, SAGs, XP-EHH, and QTL (Supplementary Fig. 20a). Many TAGs are associated with milk traits and growth development. Among them, the region including *RSRC1* and its neighbor gene *SHOX2* (short stature homeobox 2) located in chr 1 was under high selection. Polymorphism of *RSRC1* is associated with altered brain function in schizophrenia [37] and height detected in the tails [38]. *SHOX2* is involved in height and chondrogenesis [33, 39] (Fig. 4a). These finding imply the RSRC1-SHOX2 region may be related to selection for the height. Additionally, *RPL3* (ribosomal protein L3) is reported to be highly expressed in the breast milk fat globule, involved in regulation of energy balance, suggesting that translational pressure is at work during lactation [40]. Specifically, *VPS13C* (vacuolar protein sorting 13 homolog C) is suggested to act on glucose homeostasis for high milk production in dairy cows [41], and another member of the same gene family, *VPS13A*, has also been reported in pigs undergoing directional selection for heat adaptation [42]. *VPS13B* was detected

within a QTL associated with leg morphology, related with fertility and milk production in cattle and buffalo [43]. We propose that genetic variants within VPS13 family genes may have been selected during farm animal domestication and this family may play a important role for farm animal production and adaptation.

TAGs in the cashmere breed

Among the trait-related regions, there are two neighbor loci located in chr 3 with high powerful selection including no gene. The region on chr 10 with strong support contains *PRDM6* (PR domain containing 6), belonging to the PRDM family of transcriptional repressors, and is reported to be highly expressed in NOTCH1-deficient mice embryos. We expected *PRDM6* to be a candidate gene for the cashmere trait because *NOTCH1* is considered to control follicular proliferation rates and melanocyte populations [44]. Moreover, *FGF5* (fibroblast growth factor 5) stands out because it is an inhibitor of hair elongation and is associated with hair growth and length in mammals [45, 46]. To further annotate the goat *FGF5*, we performed an association analysis between exonic mutations and cashmere related traits in 224 Inner Mongolian cashmere goats by Sanger sequence. Cashmere production and body weight appear to be associated with one synonymous mutation in exon 3 of *FGF5* (Supplementary Fig. 22). The genes we described could be used as markers for improve cashmere goat production, breeding better cashmere goats, or they may be potential targets for genetic manipulation.

TAGs in the meat breed

The most important TAGs to the meat breed are four genes: *HMGXB3*, *SLC26A2*, *goat_GLEAN_10018710* and *GOAT_ENSBTAP00000044216* by TAGs, SAGs, XP-EHH, and QTL (Supplementary Fig. 18c). Mutations in the solute carrier family 26 sulphate transporter, member 2 gene (*SLC26A2*) altered residual sulphate transporter

activity, associated with short stature and skeletal dysplasias [47]. Among other trait-related genes, the region on chr 3 with the two next highest intensity signals include *SASS6*, *HIAT1*, and *SLC35A3* genes; the *HIAT1* gene may encode a novel sugar transporter and disruption causes globozoospermia and infertility in mice [48]. The mutations in the *SLC35A3* gene were associated with vertebral and multiple organ malformations in cattle and human [49, 50]. Functional characterization of these genes is likely to provide insights into improve the economic benefit of meat goat, which are appealing candidates for further investigation.

Conclusion

In this study, we interrogated whole genome sequences from three trait-driven goat breeds and assessed three categories of sequence variation SNPs, indels, and CNVs to search for the functional relevance of three categories of candidate genes SAGs, DAGs, and TAGs. First, we used several methods, including SNP desert, fixation index, pooled heterozygosity and XP-EHH, to define these candidate genes based on the allelic frequencies of the different sequence variations. Although the sampling itself is rather limited for each breed, a number of follow up studies with increased population sampling showed consistent results. Second, we grouped the breeds and the data in various ways for detailed analyses, sometimes *casting a larger net* (such as SNP desert and QTL data) and investigating discrete lists in other cases, trying to provide an overview of the genetic landscape of selection-centric genetic heterogeneity in the up-to-date molecular terminology. Third, the candidate genes we described as DAGs and TAGs are complex in function but were clearly biased toward certain functional categories. It is essential to validate them in a specialized breed with a larger population before any mechanistic studies. Finally, NGS technology provides an efficient tool for systematically deciphering the genetic background of domestication and trait selection

in a thorough way for goats and other farmed animals, and we should heckle gene ontology and expression information at the same time while we are expecting thousands of gene sequences becoming available in the years to come.

Methods

Sample collection and sequencing

We sequenced DNA samples from 19 goats: 4 from wild female goats (2 Markhor and 2 Sindh ibex) and 15 from domestic male goats (5 Saanen collected in 2008), 5 Liaoning cashmere goats (collected in 2006) and 5 Leizhou goats collected in 2007). To validate the sequence variation at the population level, we genotyped using sequencing PCR amplicons in 7 domestic breeds, including 99 Saanen, 85 Liaoning cashmere, 23 Leizhou, 16 Dera Din Panah, 30 Guanzhong, 26 Inner Mongolian cashmere, and 24 Hainan goats. The samples from Markhor and Sind ibex were collected from skin biopsies, Quetta, Pakistan. Blood samples from the domestic goats were collected in China. DNA sequences were acquired using paired-end sequencing (2×150 bp) technology on the Illumina HiSeq X10 platform. The institutional review board of the Xi'an Jiaotong University Health Science Center approved the study protocol with project identification code (2011-054).

Processing raw reads

The procedure to remove low-quality reads included meetings one or more of the following criteria: 1) N-content more than 10%; 2) >60% read length below Q7; 3) reads overlapping >10 bp with the adapter sequence and a maximum of 2 bp mismatches to the adaptor sequence; 4) paired-end reads overlapped by >10 bp with others; and 5) duplicated reads. We also trimmed up to 10 bp at the 5' -end or 30 bp at the 3' -end of a read if the local N-content was >20%.

Read mapping and quality control

We used BWA 0.5.9 (BWA, RRID:SCR_010910) to map the clean reads onto the reference genome of Capra hircus genome V1. The command 'aln -t 4 -e 10' was used to find the suffix array coordinates of the good hits of each read. Then, we used the command 'sample -a 500' to convert suffix array coordinates into chromosomal coordinates and paired reads. Other parameters were set to the defaults. We filtered the alignments as follows: 1) a mapping quality score lower than 20; 2) non-unique alignments; and 3) duplicated alignments.

Calling and validation of SNPs, indels, and CNVs

First, SNPs were called at the population scale using ANGSD, with parameters referring to a previous publication [51]. We filtered out the locus with a minimum depth <8 in all individuals and called a heterozygous SNP in one individual only when both alleles were supported by at least 4 reads. We validated the SNP calling rate (97.43%) using an NGS-based target region genotyping method by Genesky Biotechnologies (Shanghai, China). Second, Dindel v1.01 was used to call short indels (1-5 bp) in each individual [52]. We called an indel only when the non-ref allele was covered by at least 2 reads on each strand. Then, we filtered out the results that met one or more of the following three criteria: quality reported by Dindel below 20, reference homopolymer length longer than 10 bp, and length of insertion or deletion longer than 5 bp. Third, the Control-FREEC software was used to detect copy number variation (CNV) based on pair wise comparisons [53]. With a 1-kb window, we compared the coverage depth between the window and the average depth and identified CNV regions that were different from the reference. We merged the overlapped CNV regions among different samples.

Population structure analysis

We performed principal component analysis (PCA) with all population-scale autosomal SNPs using the Eigensoft package (Eigensoft, RRID:SCR_004965) [54]. The phylogenetic tree was constructed based on all autosomal SNPs, with the evolutionary distances measured by p-distance with PHYLIP (PHYLIP, RRID:SCR_006244) [55].

Definition of SNP deserts

Based on the SNP data, we computed the SNP rate in 10-kb sliding windows. We normalized the SNP rates over the length of the ≥ 8 fold aligned sequence in each bin rather than the bin size, and bins with less than 1 kb of the aligned sequence were rejected. We then selected the windows with the lowest 10% SNP rate of the genome data and joined these windows as a longer region if the gap between them was ≤10 kb. We defined these low SNP-rate windows or regions as “SNP desert”.

Selection analysis

To find a selective sweep in the domestic lines, pooled heterozygosity (Hp) and fixation index (Fst) were used to extract outliers [56]. For each 100-kb window, we determined the number of reads corresponding to the most and least abundant SNP alleles (nMAJ and nMIN), $Hp=2\sum nMAJ\sum nMIN/(\sum nMAJ+\sum nMIN)^2$. With the same 100-kb window, the Fst was calculated between 15 domestic and 4 wild goats. We then transformed Hp into ZHp: $ZHp= (Hp-\mu Hp)/ \sigma Hp$ and Fst into ZFst: $ZFst= (Fst-\mu Fst)/ \sigma Fst$. For DAG analysis, we applied a threshold of ZHp = -3 OR ZFst = 3 for detecting putative selective sweeps. For TAG analysis, we measured the pairwise Fst and tested one domestic breed and a pool of the other two breeds. The windows pass the threshold of ZFst = 4, and the top 1% XP-EHH [57]scores were extracted as candidate selective sweep regions. Genes residing in these extracted regions were indicated as candidate-

selected genes.

QTL mapping

We downloaded known sheep and cow QTL data from Animal QTLdb [58] and qualified the data by filtering out the terms with "trait association" or with *P*>0.05. We aligned the genome sequences of sheep and goat with lastz (version 1.02.00) and mapped the QTL to goat chromosomes based on the axt file produced by lastz.

Competing financial interests

The authors declare no competing financial interests.

Acknowledgments

This work was supported by the National Natural Science Foundation of China (Grant No. 31301949, No. 31272408, No. 31172184); the National Science Foundation for Post-doctoral Scientists of China (Grant No.2013M532056); and the Research Fund for the Doctor Program of Higher Education of China (No.20120204110007).

Authors Contributions

Li SB, Yu J, Zhang H, Li B, and Zhang B designed the experiments and managed the project.

Chang L, Zhang B, Fu DK, Lan XY, and Yu J performed the data analysis. Asif N, Chen H, Yan CX, Zhang XY, Huang YZ performed the phenotyping and prepared DNA samples. Chang L, Fu DK, and Guan FL, Zhang HB performed the sequencing, genotyping and validation. Zhang B, Chang L, and Yu J wrote the manuscript.

Data Accessibility

Data are available via the NCBI database, BioProject ID: PRJNA399234. SRA accession number: SRP124668. Supporting data, including also the reference assembly

and annotations, SNPs, InDels and phylogenetic tree data, are also available via the *GigaScience* repository GigaDB. [59].

Reference

1. Zeder MA and Hesse B. The Initial Domestication of Goats (Capra hircus) in the Zagros Mountains 10,000 Years Ago. Science. 2000;287 5461:2254-7. doi:10.1126/science.287.5461.2254.
2. Trut L, Oskina I and Kharlamova A. Animal evolution during domestication: the domesticated fox as a model. BioEssays : news and reviews in molecular, cellular and developmental biology. 2009;31 3:349-60. doi:10.1002/bies.200800070.
3. Taberlet P, Valentini A, Rezaei HR, Naderi S, Pompanon F, Negrini R, et al. Are cattle, sheep, and goats endangered species? Molecular ecology. 2008;17 1:275-84. doi:10.1111/j.1365-294X.2007.03475.x.
4. Hatziminaoglou Y and Boyazoglu J. The goat in ancient civilisations: from the Fertile Crescent to the Aegean Sea. Small Ruminant Research. 2004;51 2:123-9. doi:http://dx.doi.org/10.1016/j.smallrumres.2003.08.006.
5. Taberlet P, Coissac E, Pansu J and Pompanon F. Conservation genetics of cattle, sheep, and goats. Comptes rendus biologies. 2011;334 3:247-54. doi:10.1016/j.crvi.2010.12.007.
6. Decker JE, McKay SD, Rolf MM, Kim J, Molina Alcala A, Sonstegard TS, et al. Worldwide patterns of ancestry, divergence, and admixture in domesticated cattle. PLoS genetics. 2014;10 3:e1004254. doi:10.1371/journal.pgen.1004254.
7. FAO. The State of the World's Animal Genetic Resources for Food and Agriculture. 2007.
8. MacHugh DE and Bradley DG. Livestock genetic origins: goats buck the trend. Proceedings of the National Academy of Sciences of the United States of America. 2001;98 10:5382-4. doi:10.1073/pnas.111163198.
9. Fontanesi L, Martelli PL, Beretti F, Riggio V, Dall'Olio S, Colombo M, et al. An initial comparative map of copy number variations in the goat (Capra hircus) genome. BMC genomics. 2010;11:639. doi:10.1186/1471-2164-11-639.
10. Schibler L, Cribiu EP, Oustry-Vaiman A, Furet JP and Vaiman D. Fine mapping suggests that the goat Polled Intersex Syndrome and the human Blepharophimosis Ptosis Epicanthus Syndrome map to a 100-kb homologous region. Genome research. 2000;10 3:311-8.
11. Aguilar-Calvo P, Fast C, Tauscher K, Espinosa JC, Groschup MH, Nadeem M, et al. Effect of Q211 and K222 PRNP Polymorphic Variants in the Susceptibility of Goats to Oral Infection With Goat Bovine Spongiform Encephalopathy. The Journal of infectious diseases. 2015; doi:10.1093/infdis/jiv112.
12. Dong Y, Xie M, Jiang Y, Xiao N, Du X, Zhang W, et al. Sequencing and automated whole-genome optical mapping of the genome of a domestic goat (Capra hircus). Nature biotechnology. 2013;31 2:135-41. doi:10.1038/nbt.2478.
13. Bickhart DM, Rosen BD, Koren S, Sayre BL, Hastie AR, Chan S, et al. Single-molecule sequencing and chromatin conformation capture enable de novo reference assembly of the domestic goat genome. Nature genetics. 2017;49 4:643-50. doi:10.1038/ng.3802.
14. Breeds of domestic animal and poultry in China sagbiCwg. Sheep and goat breeds in China. 1988.
15. Dubeuf J-P and Boyazoglu J. An international panorama of goat selection and breeds. Livestock Science. 2009;120 3:225-31. doi:https://doi.org/10.1016/j.livsci.2008.07.005.
16. Shimizu M, Fukami T, Kobayashi Y, Takamiya M, Aoki Y, Nakajima M, et al. A novel polymorphic allele of human arylacetamide deacetylase leads to decreased enzyme activity. Drug metabolism and disposition: the biological fate of chemicals. 2012;40 6:1183-90. doi:10.1124/dmd.112.044883.
17. De Rocker N, Vergult S, Koolen D, Jacobs E, Hoischen A, Zeesman S, et al. Refinement of the critical 2p25.3 deletion region: the role of MYT1L in intellectual disability and obesity. Genetics in medicine : official journal of the American College of Medical Genetics. 2015;17 6:460-6. doi:10.1038/gim.2014.124.

18. Dong Y, Zhang X, Xie M, Arefnezhad B, Wang Z, Wang W, et al. Reference genome of wild goat (capra aegagrus) and sequencing of goat breeds provide insight into genic basis of goat domestication. BMC Genomics. 2015;16 1:431. doi:10.1186/s12864-015-1606-1.
19. Norris BJ and Whan VA. A gene duplication affecting expression of the ovine ASIP gene is responsible for white and black sheep. Genome research. 2008;18 8:1282-93. doi:10.1101/gr.072090.107.
20. Bickhart DM, Hou Y, Schroeder SG, Alkan C, Cardone MF, Matukumalli LK, et al. Copy number variation of individual cattle genomes using next-generation sequencing. Genome research. 2012;22 4:778-90. doi:10.1101/gr.133967.111.
21. Tsuiki H, Nitta M, Furuya A, Hanai N, Fujiwara T, Inagaki M, et al. A novel human nucleoside diphosphate (NDP) kinase, Nm23-H6, localizes in mitochondria and affects cytokinesis. Journal of cellular biochemistry. 1999;76 2:254-69.
22. Luikart G, Gielly L, Excoffier L, Vigne JD, Bouvet J and Taberlet P. Multiple maternal origins and weak phylogeographic structure in domestic goats. Proceedings of the National Academy of Sciences of the United States of America. 2001;98 10:5927-32. doi:10.1073/pnas.091591198.
23. Wang L, Hao L, Li X, Hu S, Ge S and Yu J. SNP deserts of Asian cultivated rice: genomic regions under domestication. Journal of evolutionary biology. 2009;22 4:751-61. doi:10.1111/j.1420-9101.2009.01698.x.
24. Matsumoto T, Sakari M, Okada M, Yokoyama A, Takahashi S, Kouzmenko A, et al. The androgen receptor in health and disease. Annual review of physiology. 2013;75:201-24. doi:10.1146/annurev-physiol-030212-183656.
25. Zhang J, Fang Z, Jud C, Vansteensel MJ, Kaasik K, Lee CC, et al. Fragile X-related proteins regulate mammalian circadian behavioral rhythms. American journal of human genetics. 2008;83 1:43-52. doi:10.1016/j.ajhg.2008.06.003.
26. Maestu J, Allik J, Merenakk L, Eensoo D, Parik J, Veidebaum T, et al. Associations between an alpha 2A adrenergic receptor gene polymorphism and adolescent personality. American journal of medical genetics Part B, Neuropsychiatric genetics : the official publication of the International Society of Psychiatric Genetics. 2008;147B 4:418-23. doi:10.1002/ajmg.b.30621.
27. Snelling WM, Allan MF, Keele JW, Kuehn LA, Thallman RM, Bennett GL, et al. Partial-genome evaluation of postweaning feed intake and efficiency of crossbred beef cattle. Journal of animal science. 2011;89 6:1731-41. doi:10.2527/jas.2010-3526.
28. Lindholm-Perry AK, Kern RJ, Kuehn LA, Snelling WM, Miles JR, Oliver WT, et al. Differences in transcript abundance of genes on BTA15 located within a region associated with gain in beef steers. Gene. 2015;572 1:42-8. doi:10.1016/j.gene.2015.06.076.
29. Desvignes L, Weidinger C, Shaw P, Vaeth M, Ribierre T, Liu M, et al. STIM1 controls T cell-mediated immune regulation and inflammation in chronic infection. The Journal of clinical investigation. 2015;125 6:2347-62. doi:10.1172/jci80273.
30. Dillon SR, Gross JA, Ansell SM and Novak AJ. An APRIL to remember: novel TNF ligands as therapeutic targets. Nature reviews Drug discovery. 2006;5 3:235-46. doi:10.1038/nrd1982.
31. Mikawa S, Morozumi T, Shimanuki S, Hayashi T, Uenishi H, Domukai M, et al. Fine mapping of a swine quantitative trait locus for number of vertebrae and analysis of an orphan nuclear receptor, germ cell nuclear factor (NR6A1). Genome research. 2007;17 5:586-93. doi:10.1101/gr.6085507.
32. Rincon G, Farber EA, Farber CR, Nkrumah JD and Medrano JF. Polymorphisms in the STAT6 gene and their association with carcass traits in feedlot cattle. Animal genetics. 2009;40 6:878-82. doi:10.1111/j.1365-2052.2009.01934.x.
33. Sanna S, Jackson AU, Nagaraja R, Willer CJ, Chen WM, Bonnycastle LL, et al. Common variants in the GDF5-UQCC region are associated with variation in human height. Nature genetics. 2008;40 2:198-203. doi:10.1038/ng.74.
34. Settle SH, Jr., Rountree RB, Sinha A, Thacker A, Higgins K and Kingsley DM. Multiple joint and skeletal patterning defects caused by single and double mutations in the mouse Gdf6 and Gdf5 genes. Developmental biology. 2003;254 1:116-30.
35. Xiong L, Jung JU, Wu H, Xia WF, Pan JX, Shen C, et al. Lrp4 in osteoblasts suppresses bone formation and promotes osteoclastogenesis and bone resorption. Proceedings of the

National Academy of Sciences of the United States of America. 2015;112 11:3487-92. doi:10.1073/pnas.1419714112.
36. Hu ZL, Park CA, Wu XL and Reecy JM. Animal QTLdb: an improved database tool for livestock animal QTL/association data dissemination in the post-genome era. Nucleic acids research. 2013;41 Database issue:D871-9. doi:10.1093/nar/gks1150.
37. Potkin SG, Turner JA, Fallon JA, Lakatos A, Keator DB, Guffanti G, et al. Gene discovery through imaging genetics: identification of two novel genes associated with schizophrenia. Molecular psychiatry. 2009;14 4:416-28. doi:10.1038/mp.2008.127.
38. Berndt SI, Gustafsson S, Magi R, Ganna A, Wheeler E, Feitosa MF, et al. Genome-wide meta-analysis identifies 11 new loci for anthropometric traits and provides insights into genetic architecture. Nature genetics. 2013;45 5:501-12. doi:10.1038/ng.2606.
39. Cobb J, Dierich A, Huss-Garcia Y and Duboule D. A mouse model for human short-stature syndromes identifies Shox2 as an upstream regulator of Runx2 during long-bone development. Proceedings of the National Academy of Sciences of the United States of America. 2006;103 12:4511-5. doi:10.1073/pnas.0510544103.
40. Wesolowski SR, Allan MF, Nielsen MK and Pomp D. Evaluation of hypothalamic gene expression in mice divergently selected for heat loss. Physiological genomics. 2003;13 2:129-37. doi:10.1152/physiolgenomics.00184.2002.
41. Lemley CO, Butler ST, Butler WR and Wilson ME. Short communication: insulin alters hepatic progesterone catabolic enzymes cytochrome P450 2C and 3A in dairy cows. Journal of dairy science. 2008;91 2:641-5. doi:10.3168/jds.2007-0636.
42. Ai H, Fang X, Yang B, Huang Z, Chen H, Mao L, et al. Adaptation and possible ancient interspecies introgression in pigs identified by whole-genome sequencing. Nature genetics. 2015;47 3:217-25. doi:10.1038/ng.3199.
43. Capitan A, Michot P, Baur A, Saintilan R, Hoze C, Valour D, et al. Genetic tools to improve reproduction traits in dairy cattle. Reproduction, fertility, and development. 2014;27 1:14-21. doi:10.1071/rd14379.
44. Lee J, Basak JM, Demehri S and Kopan R. Bi-compartmental communication contributes to the opposite proliferative behavior of Notch1-deficient hair follicle and epidermal keratinocytes. Development (Cambridge, England). 2007;134 15:2795-806. doi:10.1242/dev.02868.
45. Higgins CA, Petukhova L, Harel S, Ho YY, Drill E, Shapiro L, et al. FGF5 is a crucial regulator of hair length in humans. Proceedings of the National Academy of Sciences of the United States of America. 2014;111 29:10648-53. doi:10.1073/pnas.1402862111.
46. Hebert JM, Rosenquist T, Gotz J and Martin GR. FGF5 as a regulator of the hair growth cycle: evidence from targeted and spontaneous mutations. Cell. 1994;78 6:1017-25.
47. Barreda-Bonis AC, Barraza-García J, Parrón M, Pastor I, Heath KE and González-Casado I. Multiple SLC26A2 mutations occurring in a three-generational family. European Journal of Medical Genetics. 2018;61 1:24-8. doi:https://doi.org/10.1016/j.ejmg.2017.10.007.
48. Doran J, Walters C, Kyle V, Wooding P, Hammett-Burke R and Colledge WH. Mfsd14a (Hiat1) gene disruption causes globozoospermia and infertility in male mice. Reproduction (Cambridge, England). 2016;152 1:91-9. doi:10.1530/rep-15-0557.
49. Buck BC, Ulrich R, Wohlke A, Kuiper H, Baumgartner W and Distl O. [Vertebral and multiple organ malformations in a black and white German Holstein calf]. Berliner und Munchener tierarztliche Wochenschrift. 2010;123 5-6:251-5.
50. Edmondson AC, Bedoukian EC, Deardorff MA, McDonald-McGinn DM, Li X, He M, et al. A human case of SLC35A3-related skeletal dysplasia. American journal of medical genetics Part A. 2017;173 10:2758-62. doi:10.1002/ajmg.a.38374.
51. Liu S, Lorenzen ED, Fumagalli M, Li B, Harris K, Xiong Z, et al. Population genomics reveal recent speciation and rapid evolutionary adaptation in polar bears. Cell. 2014;157 4:785-94. doi:10.1016/j.cell.2014.03.054.
52. Albers CA, Lunter G, MacArthur DG, McVean G, Ouwehand WH and Durbin R. Dindel: accurate indel calls from short-read data. Genome research. 2011;21 6:961-73. doi:10.1101/gr.112326.110.
53. Boeva V, Popova T, Bleakley K, Chiche P, Cappo J, Schleiermacher G, et al. Control-FREEC: a tool for assessing copy number and allelic content using next-generation sequencing data.

Bioinformatics (Oxford, England). 2012;28 3:423-5. doi:10.1093/bioinformatics/btr670.
54. Patterson N, Price AL and Reich D. Population structure and eigenanalysis. PLoS genetics. 2006;2 12:e190. doi:10.1371/journal.pgen.0020190.
55. Retief JD. Phylogenetic analysis using PHYLIP. Methods in molecular biology (Clifton, NJ). 2000;132:243-58.
56. Rubin CJ, Zody MC, Eriksson J, Meadows JR, Sherwood E, Webster MT, et al. Whole-genome resequencing reveals loci under selection during chicken domestication. Nature. 2010;464 7288:587-91. doi:10.1038/nature08832.
57. Sabeti PC, Varilly P, Fry B, Lohmueller J, Hostetter E, Cotsapas C, et al. Genome-wide detection and characterization of positive selection in human populations. Nature. 2007;449 7164:913-8. doi:10.1038/nature06250.
58. Hu ZL, Park CA and Reecy JM. Developmental progress and current status of the Animal QTLdb. Nucleic acids research. 2016;44 D1:D827-33. doi:10.1093/nar/gkv1233.
59. Bao Z, Liao C, Xianyong L, Nadeem A, Fanglin G, Dongke F et al. Supporting data for "Genome-wide definition of selective sweeps reveals molecular evidence of trait-driven domestication among elite goat (Capra species) breeds for the production of dairy, cashmere, and meat" *GigaScience Database* 2018. http://dx.doi.org/10.5524/100479

Figures and Tables Legends

Figure 1 Phylogeny and population structure of goats.

(**a**) PCA based on all identified autosomal SNPs. (**b**) Neighbor-joining tree based on autosomal SNPs. SN: Saanen dairy goats, LN: Liaoning cashmere goats, and LZ: Leizhou goats. Markhor and Sindh ibex are wild goat ancestors.

Figure 2 SNP deserts of three domesticated breeds.

(**a**) SNP rate distribution. Mean and median SNP rates are labeled by peaks of distributions. (**b**) SNP desert length distribution. (**c**) *RSRC1* in a SNP desert region shared by all three breeds. SN: Saanen dairy goats, LN: Liaoning cashmere goats, and LZ: Leizhou goats.

Figure 3 Candidate regions for domestication-associated genes (DAGs).

(**a**) Distribution of Z-transformed pooled heterozygosity (ZHp) in 15 domestic goats; and Z-transformed fixation index (ZFst) between wild and domestic goats for autosomes 1 to 29. Red vertical lines indicate thresholds. (**b**) Positive end of ZFst distribution (ZFst>3) and negative end of ZHp distribution (ZHp<-3) used for extracting outliers. Dashed lines indicate cutoff values. DAGs labeled are discussed in the text.

Figure 4 Candidate selective sweep analysis for each economic breed.

Selective sweeps and their associated genes are shown in three breeds: (**a**) Saanen breed, (**b**) Liaoning cashmere breed, and (**c**) Leizhou breed. Windows passed the threshold ZFst >4 and top 1% XP-EHH scores are extracted as selective sweeps. TAGs labeled in color are discussed in the text.

Figure 5 Opposite selection for sites in *GDF5* of dairy and meat breeds.

(**a**) Nonsynonymous SNP T217C (R73G) located in first exon of *GDF5*. Amino acids at this position are highly conserved in other mammals. (**b**) Frequency diverged in different economically relevant traits. Allele T is dominant in the dairy breeds (Saanen and Guanzhong), whereas C is dominant in the meat breeds (Leizhou and Hainan). SN: Saanen goat, LN: Liaoning cashmere goat, LZ: Leizhou goat, GZ: Guanzhong goat, IM: Inner Mongolian cashmere goat, HN: Hainan goat, MA: Markhor, SI: Sindh ibex. Note: Sequencing data are as follows: LZ: CC CC CC CC CC, LN: TC CC CC TC TC, SN: TT TT TT TT TT, MA: TT TT, SI: TT TT.

Table 1 Summary of sequencing and variation for domestic and wild goats.

Note: Locations where goat breeds are farmed are labeled, Saanen or SN, Liaoning or LN, and Leizhou or LZ. Ratios of synonymous and non-synonymous SNPs are listed under NS/S.

 Click here to access/download;Table;Table.docx

Table 1 Summary of sequencing and variation for domestic and wild goats.

Group	N	Raw data (Gb)	Average Uniquely mapped bases (Gb)	Map ping rate	Mean depth	Total SNP (x10^6)	Total SNP NS/S	Indel # (x10^6)	CNV #	CNV length (Mb)
**Domestic**	15	89.75	63.43	70.67	28.84	19.04	0.86	1.54	2,028	32.0
Dairy_SN	5	89.23	67.79	75.97	29.11	11.38	0.82	1.02	1,161	20.5
Cashmere_LN	5	88.46	67.33	76.11	28.46	12.33	0.83	1.02	1,096	18.5
Meat_LZ	5	91.56	68.36	74.66	28.96	9.19	0.85	0.83	1,725	21.8
**Wild**	4	94.72	67.29	71.04	28.64	11.66	0.97	0.99	1,616	19.2
Markhor	2	86.27	62.71	72.69	26.89	4.42	0.87	0.64	1,220	15.4
Sindh ibex	2	103.16	71.87	69.66	30.39	7.63	1.06	0.73	1,352	15.3
**Total**	19	90.80	64.24	70.75	28.80	23.92	0.95	1.90	2,317	35.5

Note: Locations where goat breeds are farmed are labeled, Saanen or SN, Liaoning or LN, and Leizhou or LZ. Ratios of synonymous and non-synonymous SNPs are listed under NS/S.

Figure Click here to access/download;Figure;Figure 1.docx

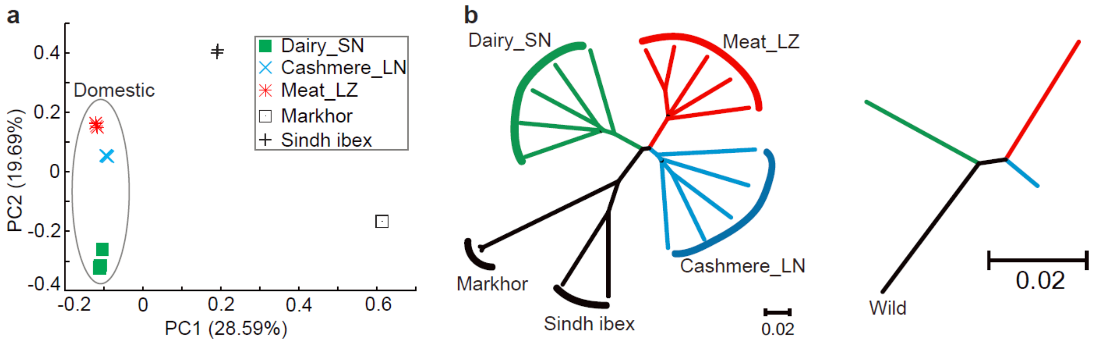

Figure 1 Phylogeny and population structure of goats. (**a**) PCA based on all identified autosomal SNPs. (**b**) Neighbor-joining tree based on autosomal SNPs. SN: Saanen dairy goats, LN: Liaoning cashmere goats, and LZ: Leizhou goats. Markhor and Sindh ibex are wild goat ancestors.

 Click here to access/download;Figure;Figure 2.docx

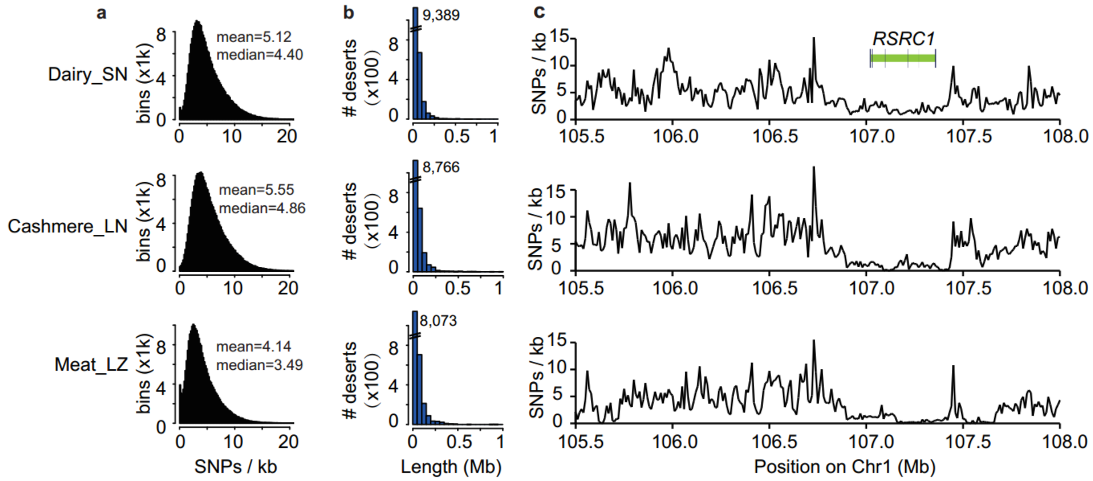

Figure 2 SNP deserts of three domesticated breeds. (a) SNP rate distribution. Mean and median SNP rates are labeled by peaks of distributions. (b) SNP desert length distribution. (c) *RSRC1* in a SNP desert region shared by all three breeds. SN: Saanen dairy goats, LN: Liaoning cashmere goats, and LZ: Leizhou goats.

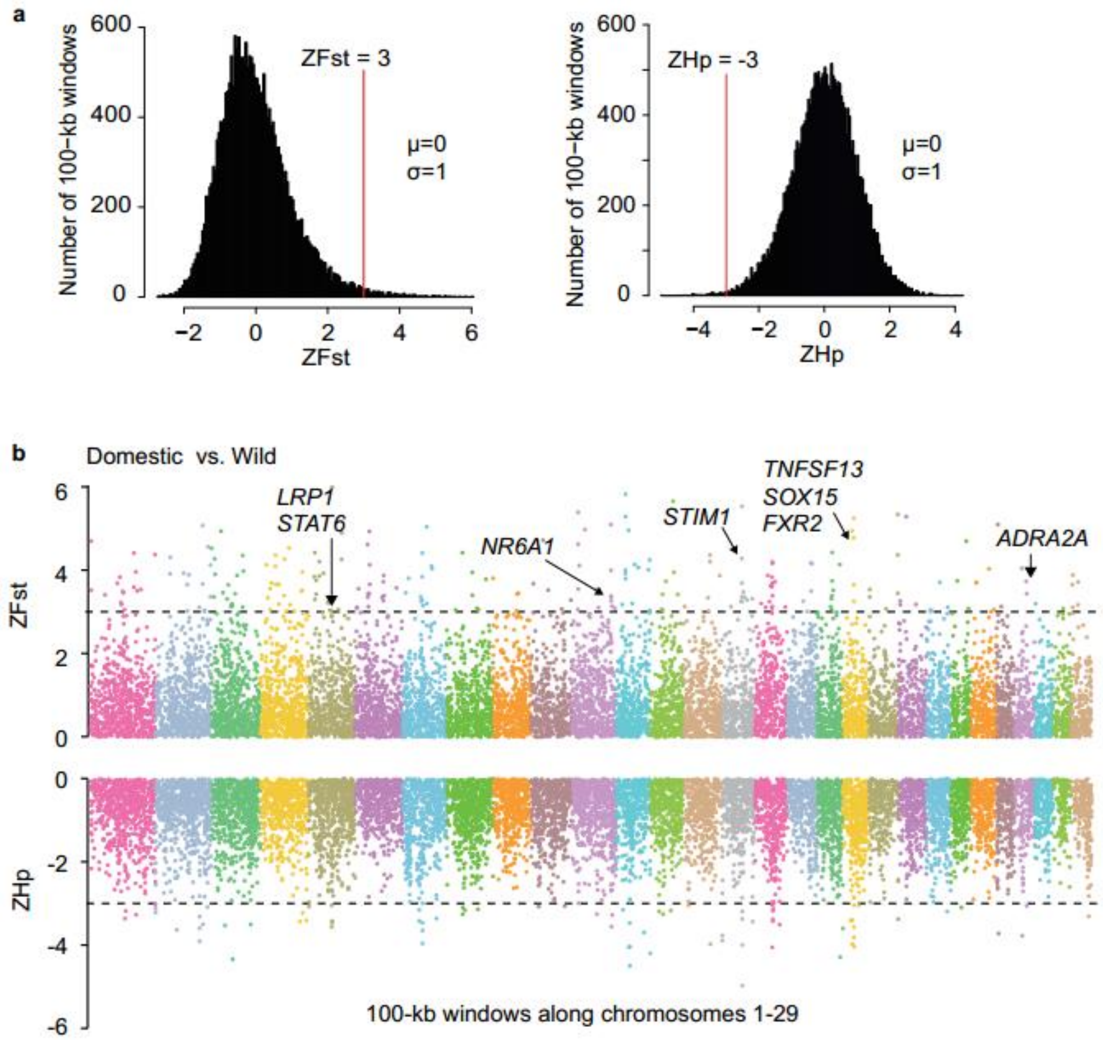

Figure 3 Candidate regions for domestication-associated genes (DAGs). (a) Distribution of Z-transformed pooled heterozygosity (ZHp) in 15 domestic goats; and Z-transformed fixation index (ZFst) between wild and domestic goats for autosomes 1 to 29. Red vertical lines indicate thresholds. (b) Positive end of ZFst distribution (ZFst>3) and negative end of ZHp distribution (ZHp<-3) used for extracting outliers. Dashed lines indicate cutoff values. DAGs labeled are discussed in the text.

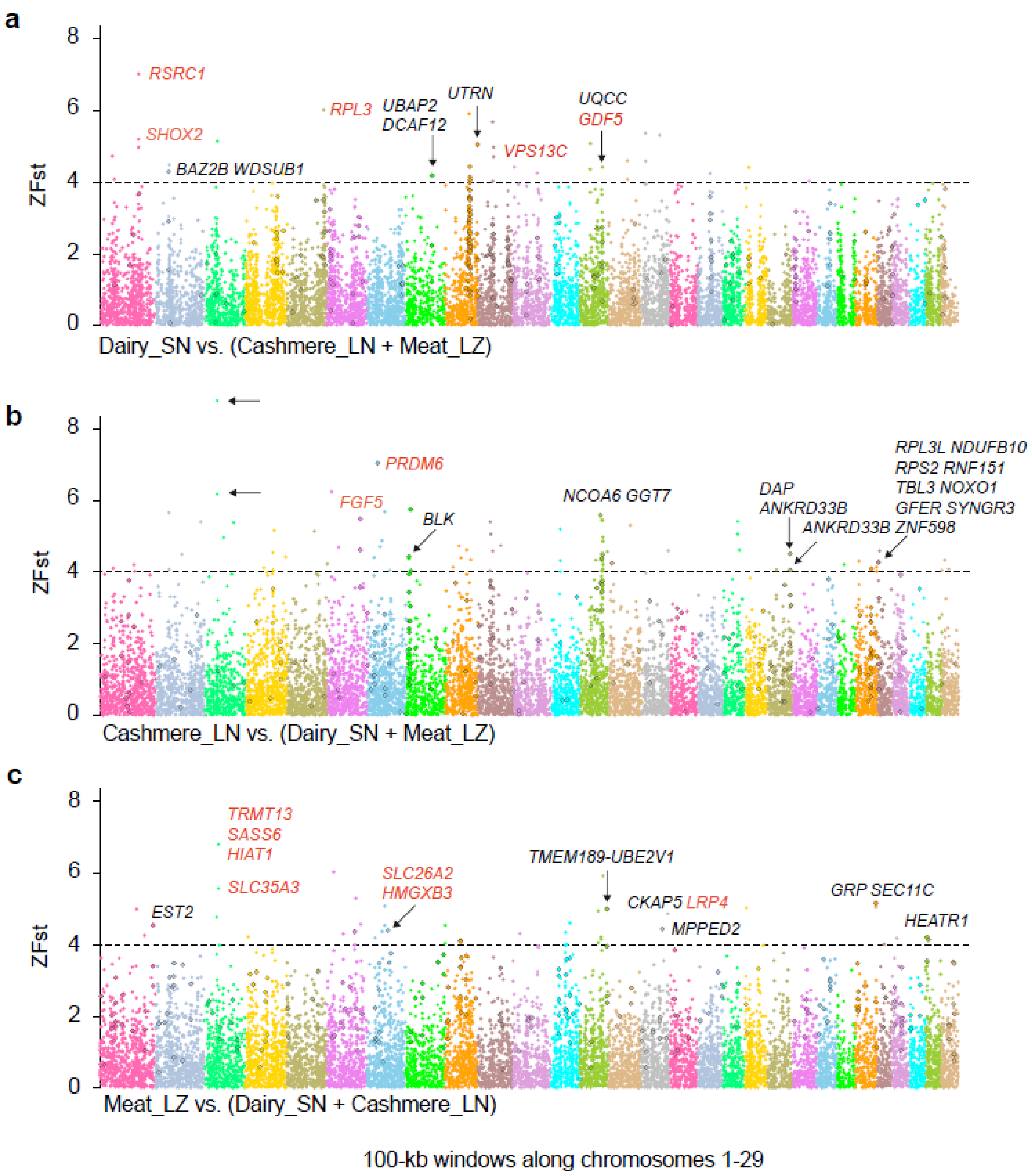

Figure 4 Candidate selective sweep analysis for each economic breed. Selective sweeps and their associated genes are shown in three breeds: (a) Saanen breed, (b) Liaoning cashmere breed, and (c) Leizhou breed. Windows passed the threshold ZFst >4 and top 1% XP-EHH scores are extracted as selective sweeps. TAGs labeled in color are discussed in the text.

Figure Click here to access/download;Figure;Figure 5.docx

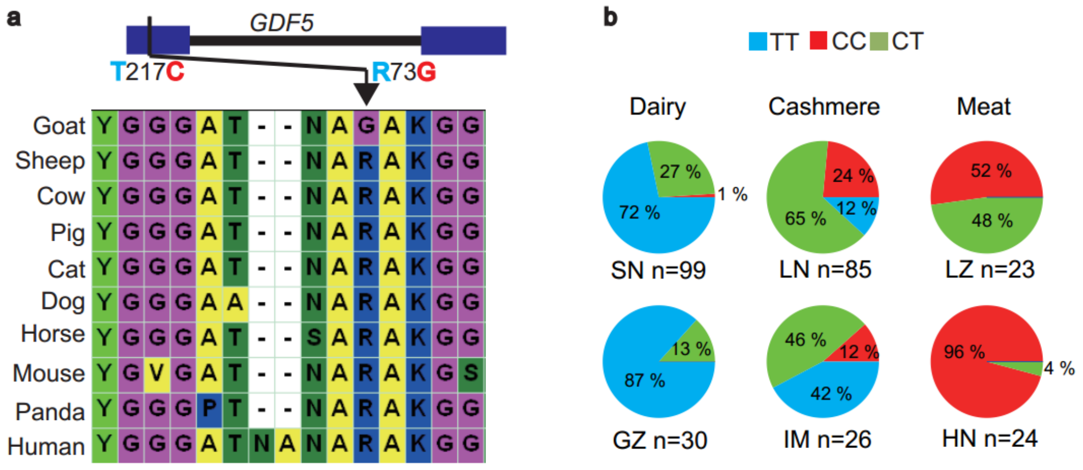

Figure 5 Opposite selection for sites in GDF5 of dairy and meat breeds. (a) Nonsynonymous SNP T217C (R73G) located in first exon of *GDF5*. Amino acids at this position are highly conserved in other mammals. (b) Frequency diverged in different economically relevant traits. Allele T is dominant in the dairy breeds (Saanen and Guanzhong), whereas C is dominant in the meat breeds (Leizhou and Hainan). SN: Saanen goat, LN: Liaoning cashmere goat, LZ: Leizhou goat, GZ: Guanzhong goat, IM: Inner Mongolian cashmere goat, HN: Hainan goat, MA: Markhor, SI: Sindh ibex.

Note: Sequencing data are as follows: LZ: CC CC CC CC CC, LN: TC CC CC TC TC, SN: TT TT TT TT TT, MA: TT TT, SI: TT TT.

Click here to access/download
Supplementary Material
Goat_Supplnote-4.10.doc
