# Supplementary material for: Genome-wide definition of selective sweeps reveals molecular evidence of trait-driven domestication among elite goat (Capra species) breeds for the production of dairy, cashmere, and meat
Source: Gigascience. 2018 Aug 27;7(12):giy105. doi: 10.1093/gigascience/giy105 (PMC6287099; doi:10.1093/gigascience/giy105)
Supplement: Supplement File [file giy105_supplement_file.doc]

**Genome-wide definition of selective sweeps reveals molecular evidence of trait-driven domestication among elite goat (*Capra* species) breeds for the production of dairy, cashmere, and meat**

**Zhang Bao^1^**^+^**, Chang Liao^1^**^+^**, Lan Xianyong^2^**^+^**, Asif Nadeem^3^, Guan Fanglin^1^, Fu Dongke^1^, Li Bo^1^, Yan Chunxia^1^, Zhang Hongbo^1^, Zhang Xiaoyan^2^, Huang Yongzhen^2^, Chen Hong^2^, Yu Jun^4^*, Li Shengbin^1^***

**1. College of Medicine & Forensic, Health Science Center, Xi’an Jiaotong University, Xi’an, Shaanxi, People's Republic of China;**

**2. Northwest A&F University, Shaanxi Key Laboratory of Molecular Biology for Agriculture, Yangling, Shaanxi, People's Republic of China;**

**3. Institute of Biochemistry and Biotechnology, University of Veterinary and Animal Sciences, Lahore, Pakistan;**

**4.** **CAS Key Laboratory of Beijing Institute of Genomics,** **Chinese Academy of Sciences, Beijing, People's Republic of China.**

**+These authors contributed equally to this work**

***co-corresponding authors, correspondence and requests for materials should be addressed to: shbinlee@mail.xjtu.edu.cn;** [**junyu@big.ac.cn**](mailto:junyu@big.ac.cn)

[1. SUPPLEMENTARY FIGURES 5](#__RefHeading___Toc511137550)

[Supplementary Figure 1. The distribution of the wild and domestic goats used in this study. 5](#__RefHeading___Toc511137551)

[Supplementary Figure 2. Introduction of wild and domestic goats sequenced in this study. 6](#__RefHeading___Toc511137552)

[Supplementary Figure 3. Sequencing depth distribution of domestic and wild goats. 10](#__RefHeading___Toc511137553)

[Supplementary Figure 4. Minor allele frequencies (MAFs) of SNPs among the domestic breeds. 10](#__RefHeading___Toc511137554)

[Supplementary Figure 5. Ratio of nonsynonymous and synonymous SNPs at different MAFs. 11](#__RefHeading___Toc511137555)

[Supplementary Figure 6. Ratio of SNP-desert associated nonsynonymous and synonymous SNPs at different MAFs shared among the domestic breeds. 11](#__RefHeading___Toc511137556)

[Supplementary Figure 7. A Venn diagram of SNPs among the domestic breeds. 12](#__RefHeading___Toc511137557)

[Supplementary Figure 8. Distribution of total and breed-specific SNPs. 12](#__RefHeading___Toc511137558)

[Supplementary Figure 9. Distribution of indels found among the domestic breeds. 13](#__RefHeading___Toc511137559)

[Supplementary Figure 10. Length distribution of indels in domestic population. 13](#__RefHeading___Toc511137560)

[Supplementary Figure 11. A Venn diagram of domestication-specific short indels. 14](#__RefHeading___Toc511137561)

[Supplementary Figure 12. A Venn diagram of genes related to domestication-specific CNVs. 14](#__RefHeading___Toc511137562)

[Supplementary Figure 13. Validation of ASIP copy number in a larger population size from 4 domestic breeds. 15](#__RefHeading___Toc511137563)

[Supplementary Figure 14. SNP rate distribution with variable sliding windows. 16](#__RefHeading___Toc511137564)

[Supplementary Figure 15. A Venn diagram of SNP-desert-associated genes (SAGs) in the domestic breeds. 17](#__RefHeading___Toc511137565)

[Supplementary Figure 16. SNP rate distribution of *RSRC1* gene. 17](#__RefHeading___Toc511137566)

[Supplementary Figure 17. A Venn diagram of SAGs and domestication-associated genes (DAGs). 18](#__RefHeading___Toc511137567)

[Supplementary Figure 18. GO enrichment analysis of DAGs. 18](#__RefHeading___Toc511137568)

[Supplementary Figure 19. Opposite selection for LRP4 in the cashmere and meat breeds. 20](#__RefHeading___Toc511137569)

[Supplementary Figure 20. A Venn diagram of SAGs, QTL-associated, and trait-associated genes (TAGs). 20](#__RefHeading___Toc511137570)

[Supplementary Figure 21. GO categories analysis of genes shared by SAGs and QTL 21](#__RefHeading___Toc511137571)

[Supplementary Figure 22. Association between phenotype and genotype of the 699th nucleotide of FGF5. 21](#__RefHeading___Toc511137572)

[2. SUPPLEMENTARY TABLES 22](#__RefHeading___Toc511137573)

[Supplementary Table 1. Summary of samples and sequencing. 22](#__RefHeading___Toc511137574)

[Supplementary Table 2.Non-synonymous/synonymous SNP ratios across groups. 22](#__RefHeading___Toc511137575)

[Supplementary Table 3. Heterozygosity in each individual. 23](#__RefHeading___Toc511137576)

[Supplementary Table 4. Distribution of breed-specific indels. 23](#__RefHeading___Toc511137577)

[Supplementary Table 5. Genes with frame-shift of the domestic breeds (specific≥60% mutation rate). 23](#__RefHeading___Toc511137578)

[Supplementary Table 6. CNV distribution in each individual. 24](#__RefHeading___Toc511137579)

[Supplementary Table 7. CNV associated genes of domestic breeds (≥60% frequency). 25](#__RefHeading___Toc511137580)

[Supplementary Table 8. CNV association with economic trait in the dairy breeds. 26](#__RefHeading___Toc511137581)

[Supplementary Table 9. The distribution of genes in SNP deserts. 27](#__RefHeading___Toc511137582)

[Supplementary Table 10. GO enrichment of the breed-shared SAGs. 27](#__RefHeading___Toc511137583)

[Supplementary Table 11. SAGs in the top 10 larger SNP deserts of the cashmere breed. 28](#__RefHeading___Toc511137584)

[Supplementary Table 12. SAGs in the top 10 larger SNP deserts of the dairy breed. 29](#__RefHeading___Toc511137585)

[Supplementary Table 13. SAGs in the top 10 larger SNP deserts of the meat breed. 30](#__RefHeading___Toc511137586)

[Supplementary Table 14. A list of genes overlapping between DAGs and SAGs. 31](#__RefHeading___Toc511137587)

[Supplementary Table 15. GO enrichment of DAGs. 33](#__RefHeading___Toc511137588)

[Supplementary Table 16. TAGs in chromosomal regions. 34](#__RefHeading___Toc511137589)

[3. SUPPLEMENTARY DISCUSSION 36](#__RefHeading___Toc511137590)

[3.1 SNP-desert-associated genes (SAGs) 36](#__RefHeading___Toc511137591)

[3.2 Domestication-associated genes (DAGs) 36](#__RefHeading___Toc511137592)

[4. SUPPLEMENTARY METHODS 38](#__RefHeading___Toc511137593)

[4.1 Samples 38](#__RefHeading___Toc511137594)

[4.2 Sequencing and analysis 39](#__RefHeading___Toc511137595)

[4.2.1 Sequencing 39](#__RefHeading___Toc511137596)

[4.2.2 Read mapping 39](#__RefHeading___Toc511137597)

[4.2.3 Sequence variation 39](#__RefHeading___Toc511137598)

[4.3 Experimental validation of SNP calling 40](#__RefHeading___Toc511137599)

[4.4 Phylogenetic analysis 40](#__RefHeading___Toc511137600)

[4.5 SNP desert and SNP-desert-associated genes (SAGs) 40](#__RefHeading___Toc511137601)

[4.6 Domestication-associated genes or DAGs 41](#__RefHeading___Toc511137602)

[4.7 Trait-associated genes (TAGs) 41](#__RefHeading___Toc511137603)

[4.7.1 Cross-population extended haplotype homozygosity (XP-EHH) scan 42](#__RefHeading___Toc511137604)

[4.7.2 QTL mapping 42](#__RefHeading___Toc511137605)

[4.8 Signatures of artificial selection Validation Samples 42](#__RefHeading___Toc511137606)

[4.9 Copy number variations and association analysis 42](#__RefHeading___Toc511137607)

[5. REFERENCES 43](#__RefHeading___Toc511137608)

# SUPPLEMENTARY FIGURES

## Supplementary Figure 1. The distribution of the wild and domestic goats used in this study.


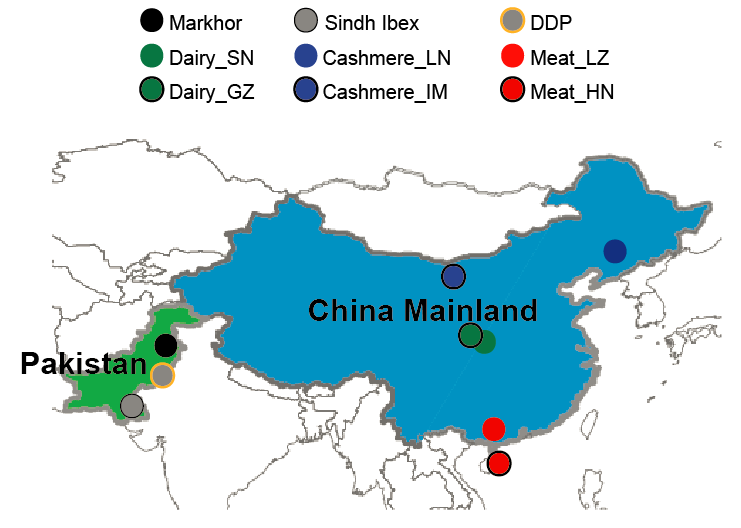


Note: 1. Dairy_SN, Cashmere_LN, Meat_LZ, Markhor, and Sindh Ibex are sequenced, other goat breeds used for validation in this study;

2. Two wild (Markhor, Sindh Ibex) and one domestic breed (DDP) were sampled from Pakistan. The other six domestic breeds were collected from China.

3. DDP: Dera Din Panah goat; Dairy_SN: Saanen goat; Dairy_GZ: Guanzhong goat; Cashmere_LN: Liaoning cashmere goat; Cashmere_IM: Inner Mongolian cashmere goat; Meat_LZ: Leizhou goat; Meat_HN: Hainan goat.

## Supplementary Figure 2. Introduction of wild and domestic goats sequenced in this study.

|  | Saanen goat | Liaoning cashmere goat | Leizhou goat | Sindh Ibex goat | Markhor goat |
| --- | --- | --- | --- | --- | --- |
| **Appearance** | 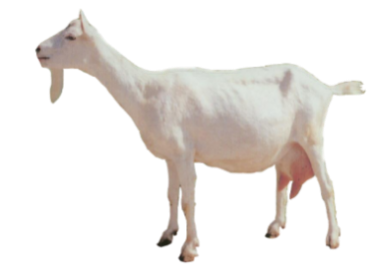 | 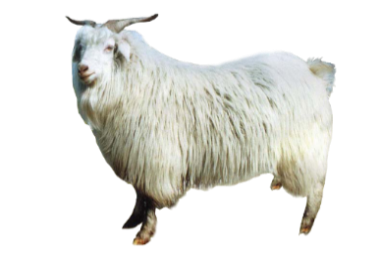 | 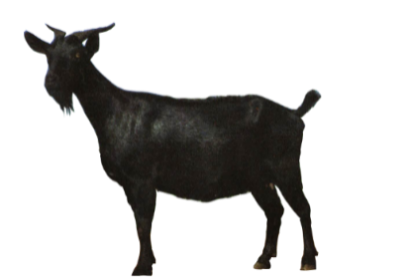 | 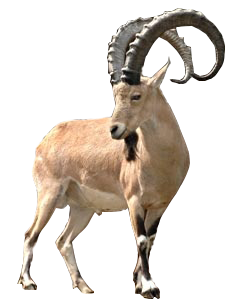 | 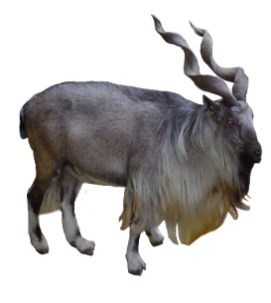 |
| **Breed history** | - Originally, grow in Switzerland. - Introduce to China from Canada in 1929. - Saanen goat is with large physique and milk production. It has strong adaptability to environment and genetic stability. | - Indigenous to the southeast mountain area of Liaoning province in China. - Liaoning cashmere goat has undergone artificial selection. | - Indigenous to the Zhanjiang area of Guangdong province in China. - Leizhou goat has undergone artificial selection for many years and is suitable to the tropical ecological environment. | - This wild goat is gregarious. | - A large species of wild goat. |
| **Characteristics** | - Most hornless. - The average adult body weight of female is 62.86±6.83 kg, and male is 98.56±11.74 kg. - The average body length is 107.17 cm (n=70). - One of the most famous breeds for goat milk. The average lactation period of the female is 300 days, the average milk production is above 800 kg each delivery and 3 to 5 kg per day. | - White color. - The average adult body weight is about 50 kg (female is 44.86±0.81 kg, male is 51.66±1.13 kg). - The body length of the female is 72.78±0.53 cm and the male is 75.74±0.57 cm. The body height of the female is 60.80±0.42 cm and the male is 63.62±0.59 cm. - The large quantity and high quality of cashmere production in China. The average cashmere production of the adult female is 0.49±0.01 kg and the male is 0.57±0.02 kg. The average wool production of the adult female is 0.40±0.01 kg and the male is 0.47±0.02 kg. | - Black color. - The average adult body weight of female is 47.70 kg, and male is 54.00 kg. - The body length of the female is 58.1 cm and the male is 62.10 cm. - The body height of the female is 55.90 cm and the male is 60.50 cm. - The mutton is excellent. The fat is well distributed. The cutis plate is closed and full of elasticity. The area of the cutis plate is large and with high utilization. | - Stocky animals with thickset bodies and strong limbs. - Yellowish-brown varying to reddish-grey with a darker brown mid-dorsal line extending from the shoulders to the base of the tail. - With long sweep scimitar shaped horns over 102cm in length. - Males have short beards, but females lack any beard. - There is also a conspicuous black stripe in adult males, running from the withers down the front of the shoulders and merging with the black chest. | - Grizzled, light brown to black color. - They all have tightly curled corkscrew-like horns. - Large body size. - 65 to 115 cm at the shoulder, 132 to 186 cm in length and weigh from 32 to 110 kg. - The national animal of Pakistan. - The species was classed by the IUCN as Endangered. |
| **Reproduction** | - The female comes to sexual maturity in 3 to 4 months. The male has sexual behavior at 3 months. The birth rate of lamb is 200%. - They often crossbreed with the local goat and become the paternal breed of Guanzhong dairy goat and Lao Shan dairy goat. | - First sexual maturity at the age of 5 months. - The breeding periodic about 7 to 8 years old. - One foetus per year, the lambing rate was 118.3%; the survival rate is more than 95%. | - Calve at the first year. - Most is [didymous](javascript:void(0);), and a few are triplet every two years. - The average litter size is 1 to 2; the maximum is up to 5. - The birth rate of lamb is 150% to 200%. - The limitation year of breeding of female is 7 to 8 years, and male is 4 to 6 years. |  | - Females gestate for 135-170 days and give birth typically to 1-2 kids. - Animals are sexually mature at 18-30 months, and live 12-13 years. - Markhor is also the ancestor of some breeds of domestic goat. |
| **Current distribution** | - The most widespread milk goat. - Distributed in almost all countries around the world except the very hot or very cold districts. | - Widely distributed in Liaodong peninsula, including Gai County, Xiuyan County, Fu County, Zhuanghe County, Fengcheng County, Liaoyang County. | - Distributed in Leizhou Peninsula and Hainan Island. | - West of the Indus River in southern Pakistan and the adjoining Chagai Hills of southern Afghanistan. | - Northeastern Afghanistan, northern India, northern and central Pakistan, southern Tajikistan and southern Uzbekistan. |

References：

1. Sheep and goat breeds in China (1988).

2. <http://www.iucnredlist.org/details/3787/0> (The IUCN Red List of Threated Species-(Capra faconeri).

3. <http://en.wikipedia.org/wiki/Markhor>.

4. Pesmen, G. and M. Yardimci, Estimating the live weight using somebody measurements in Saanen goats. Archiva Zootechnica, 2008. 11(4): p. 30-4

## Supplementary Figure 3. Sequencing depth distribution of domestic and wild goats.


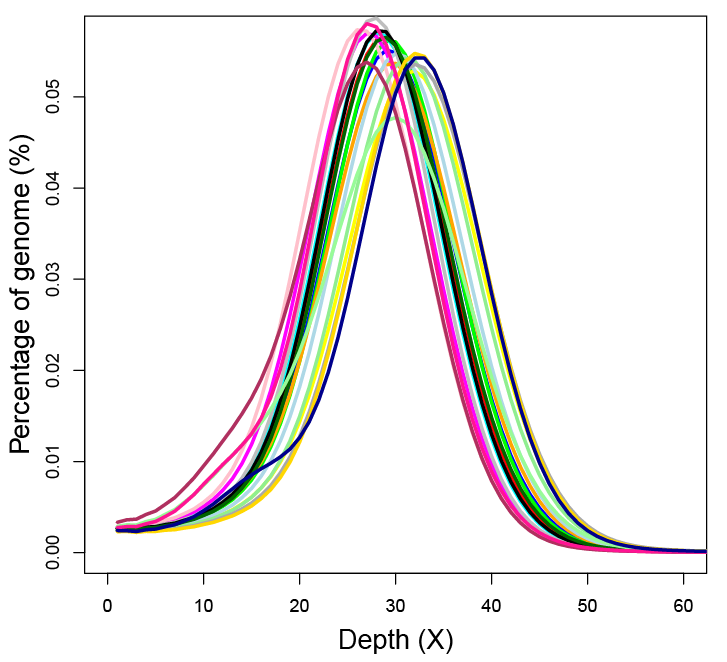


Note: We include 15 domestic and 4 wild goats in this study. Each color-coded curve represents an individual sample. Note that most of the peaks are within 20x to 35x genome coverage.

## Supplementary Figure 4. Minor allele frequencies (MAFs) of SNPs among the domestic breeds.


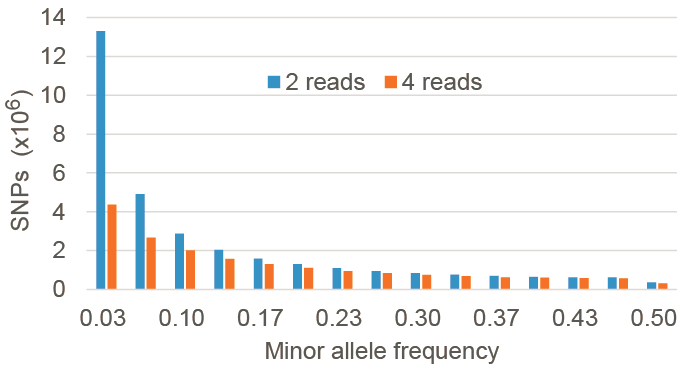


Note: We show the distribution where SNPs are supported by 2 (blue) or 4 (orange) reads. Only SNPs with a minimum depth> or = 8 in every individual sample were taken account of. The 2-read covered SNPs may have significant amount of false positives.

## Supplementary Figure 5. Ratio of nonsynonymous and synonymous SNPs at different MAFs.


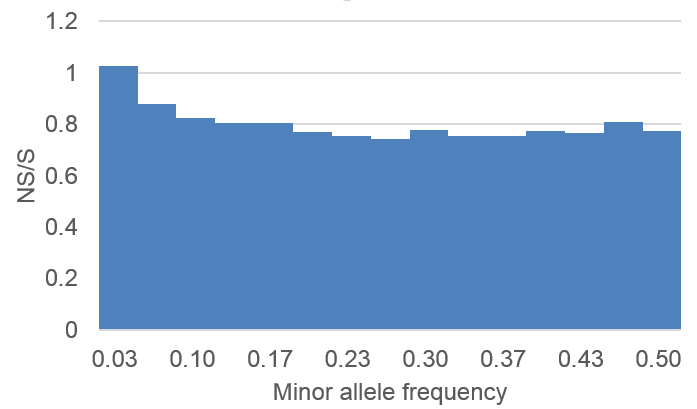


Note: We removed high-MAF SNPs caused by gene family, pseudogenes and mis-assembly.

## Supplementary Figure 6. Ratio of SNP-desert associated nonsynonymous and synonymous SNPs at different MAFs shared among the domestic breeds.


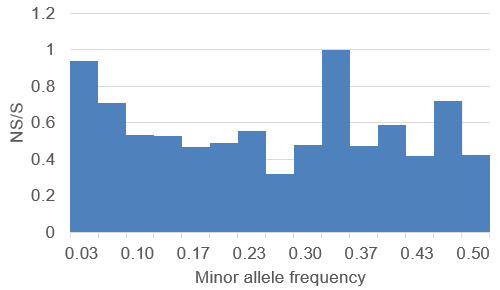


Note: There are strong negative selections in the SNP desert except a couple of clusters associated with specific MAFs.

## Supplementary Figure 7. A Venn diagram of SNPs among the domestic breeds.


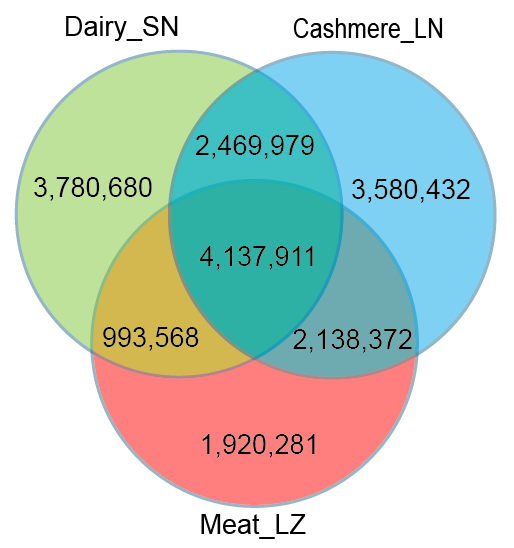


Note: The cashmere breed has the highest number of SNPs than those of the dairy and meat breeds.

## Supplementary Figure 8. Distribution of total and breed-specific SNPs.


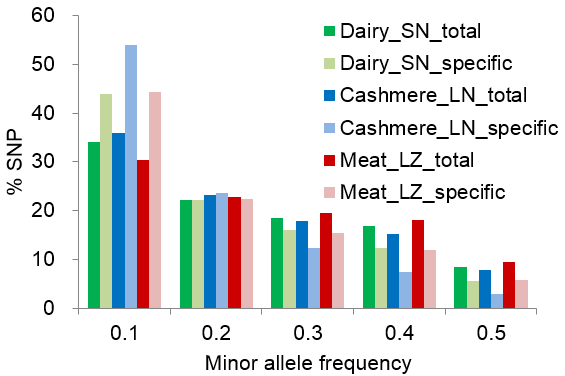


Note that at the low MAF, there are more breed-specific SNPs than the total but a transition is found when SNP frequency becomes greater than 0.2, where the breed-specific SNPs becomes less than that of the total. In addition, the meat-breed has more ancient SNPs with higher frequency than the other two breeds.

## Supplementary Figure 9. Distribution of indels found among the domestic breeds.


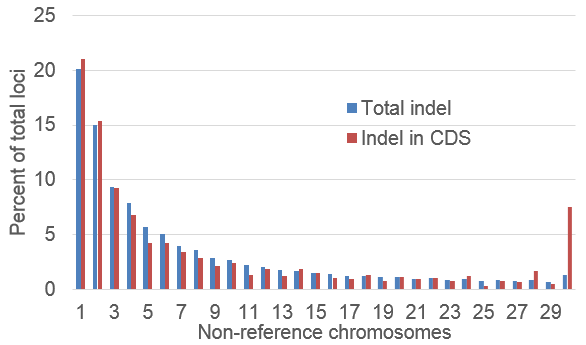


Note: Non-referenced indels among chromosomes are partitioned into the total (blue) and what found in CDS (red). Most indels are rare in the population.

## Supplementary Figure 10. Length distribution of indels in domestic population.

Total Indel

Indel in CDS

**
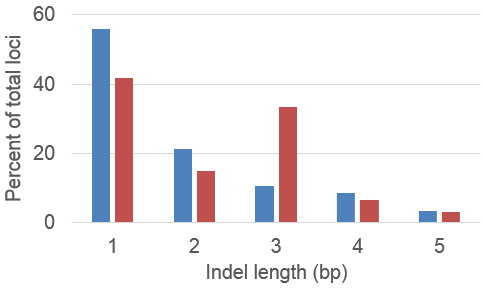
**

Note: Of all indels, the discovery rate is inversely correlated with length. Of the total found in CDS, 3-bp indels are more common than 2-bp, 4-bp, and 5-bp but not 1-bp indels.

## Supplementary Figure 11. A Venn diagram of domestication-specific short indels.


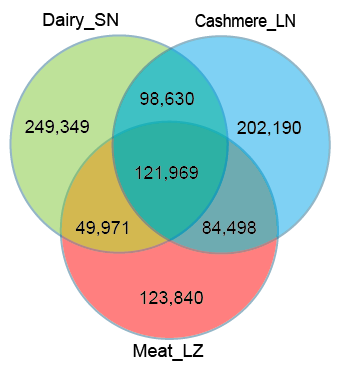


Note that the dairy breed appears to have slightly more indels than the other two breeds as opposed to in the case of SNPs, where the cashmere breed takes a lead.

## Supplementary Figure 12. A Venn diagram of genes related to domestication-specific CNVs.


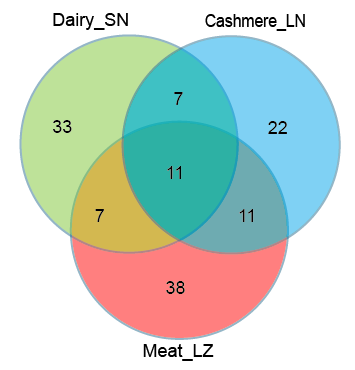


Note that the meat breed appears to have more CNVs than the other two breeds.

## Supplementary Figure 13. Validation of ASIP copy number in a larger population size from 4 domestic breeds.


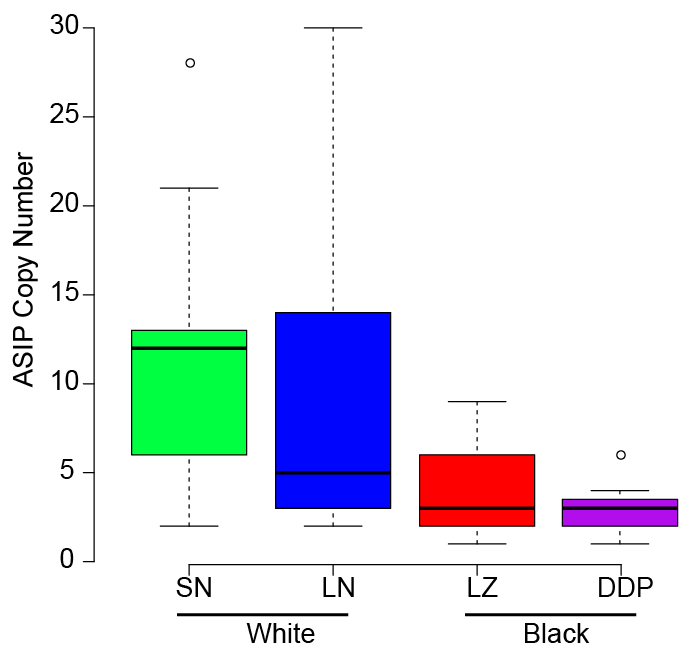


Note: The samples are Saanen goats (n=13) and Liaoning cashmere goats (n=12) with white coat, Leizhou goats (n=13) and DDP goats (n=16) with black coat.

## Supplementary Figure 14. SNP rate distribution with variable sliding windows.


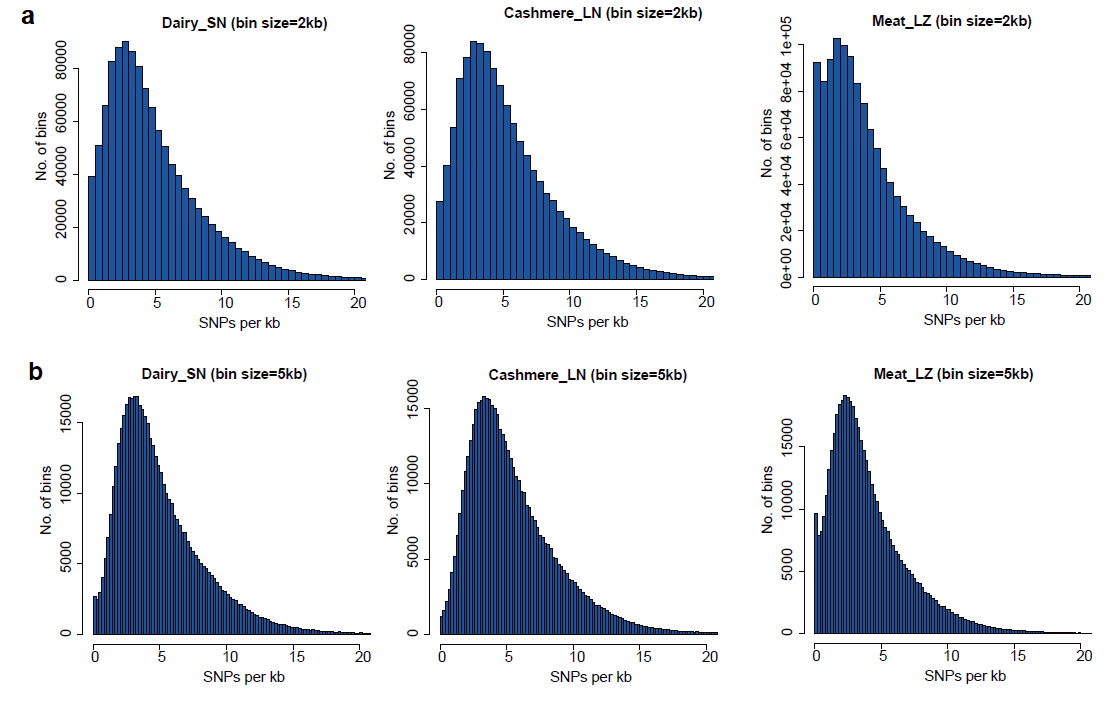


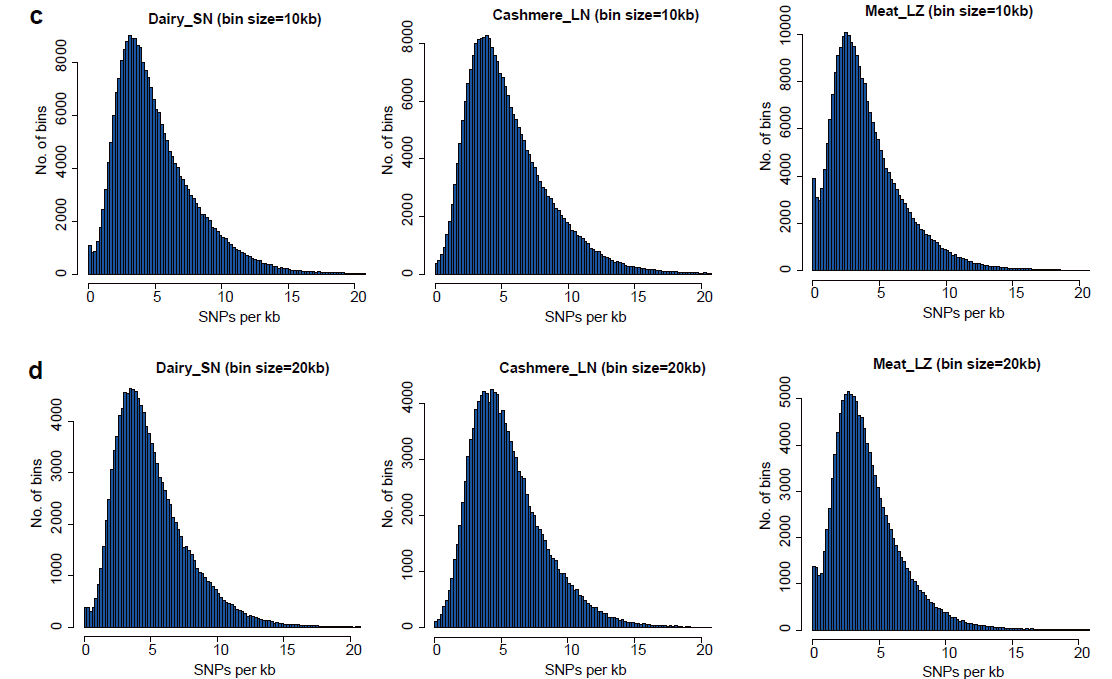


Note that the distribution of the cashmere breed is different from the other two breed where it does not have the minor peak or the low frequency SNPs. The meat breed appears possessing more diverse genetic background than the other two breeds.

## Supplementary Figure 15. A Venn diagram of SNP-desert-associated genes (SAGs) in the domestic breeds.


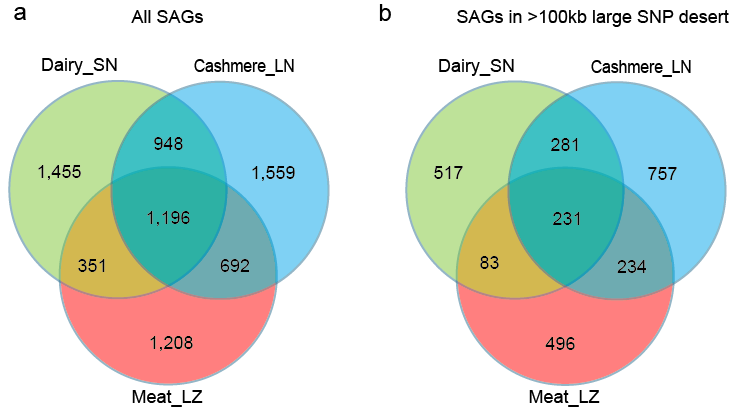


Note that the cashmere breed has more recent SAGs than the other two breeds and that the older breed, the meat, has the least number of SAGs.

## Supplementary Figure 16. SNP rate distribution of *RSRC1* gene.


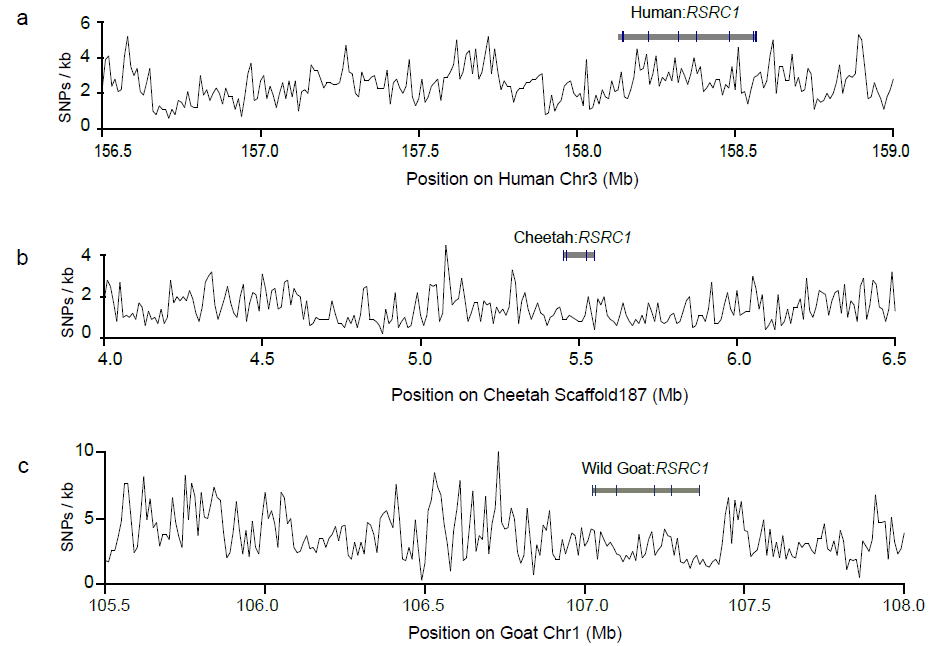


Note: (a) human; (b) cheetah and (c) wild goat.

## Supplementary Figure 17. A Venn diagram of SAGs and domestication-associated genes (DAGs).


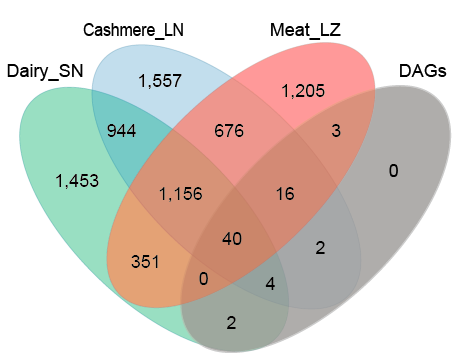


Note that all three domestic breeds share most of the DAGs.

## Supplementary Figure 18. GO enrichment analysis of DAGs.


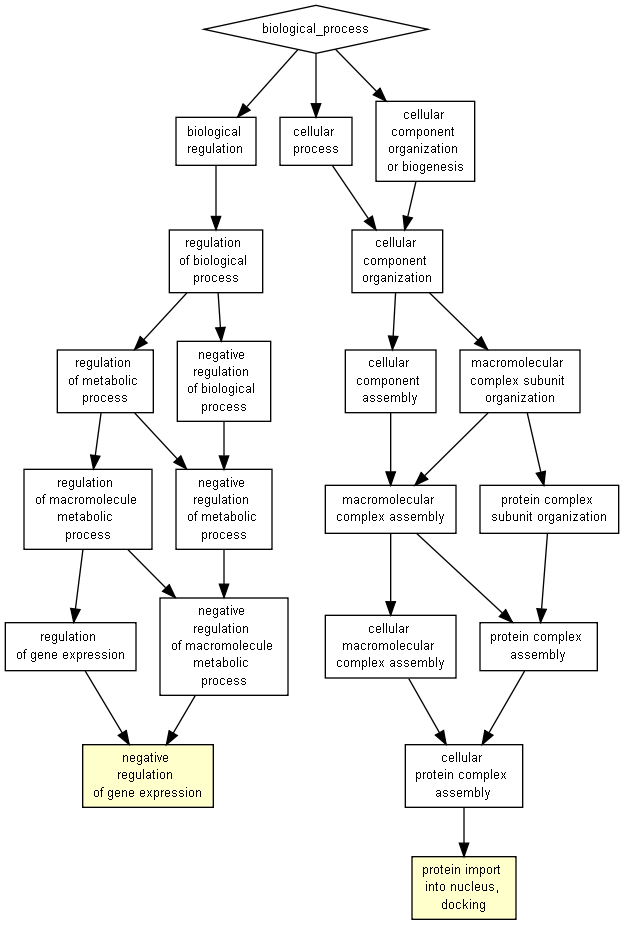


Note: The GO terms with yellow shadow indicate that FDR q-value < 10^-3^.

## Supplementary Figure 19. Opposite selection for LRP4 in the cashmere and meat breeds.

**
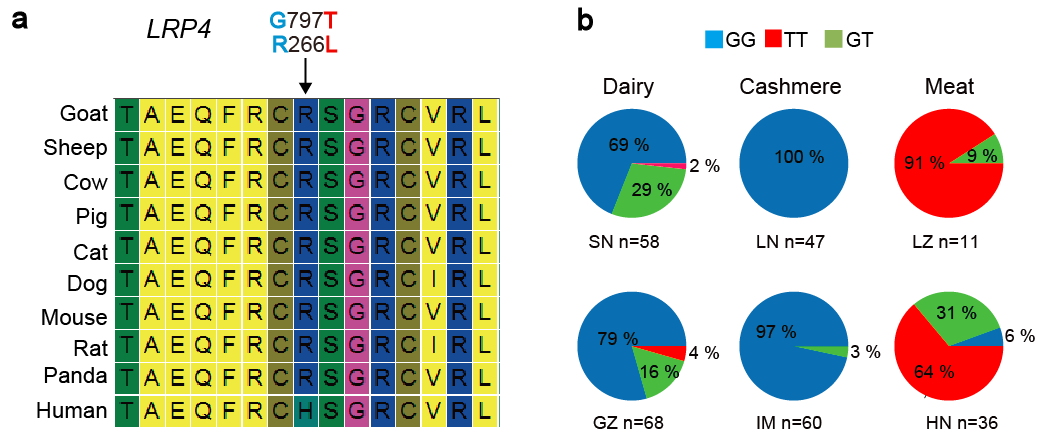
**

Note: (a) A nonsynonymous SNP R266L (G797T) located in LRP4. The amino acid at this position is highly conserved among mammals. (b) Frequency divergence happened in different economically relevant traits. Allele G is dominant. SN: Saanen goat, LN: Liaoning cashmere goat, LZ: Leizhou goat, GZ: Guanzhong goat, IM: Inner Mongolian cashmere goat, HN: Hainan goat, MA: Markhor, SI: Sindh ibex.

Note: the sequencing data is as following LZ: AA AA AA AA AA, LN: CC CC CC CC CC, SN: CC CC CC CC CC, MA: CC CC, SI: CC CC.

## Supplementary Figure 20. A Venn diagram of SAGs, QTL-associated, and trait-associated genes (TAGs).


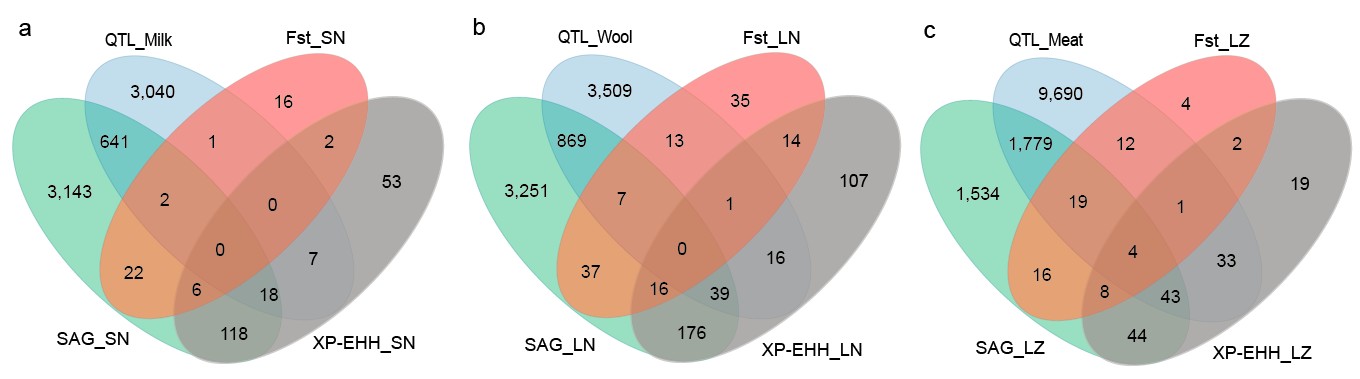


Note that significant amounts of genes are found overlapping between QTL and SAGs.

## Supplementary Figure 21. GO categories analysis of genes shared by SAGs and QTL


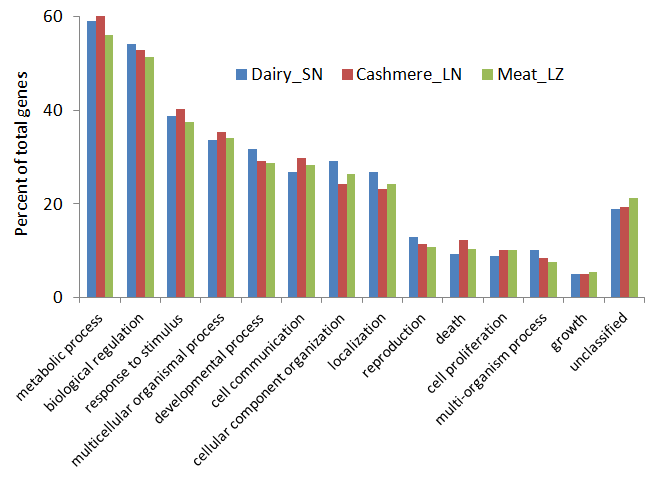


Note: the overlap part of SAGs and QTL in Supplementary Figure 20.

## Supplementary Figure 22. Association between phenotype and genotype of the 699th nucleotide of FGF5.


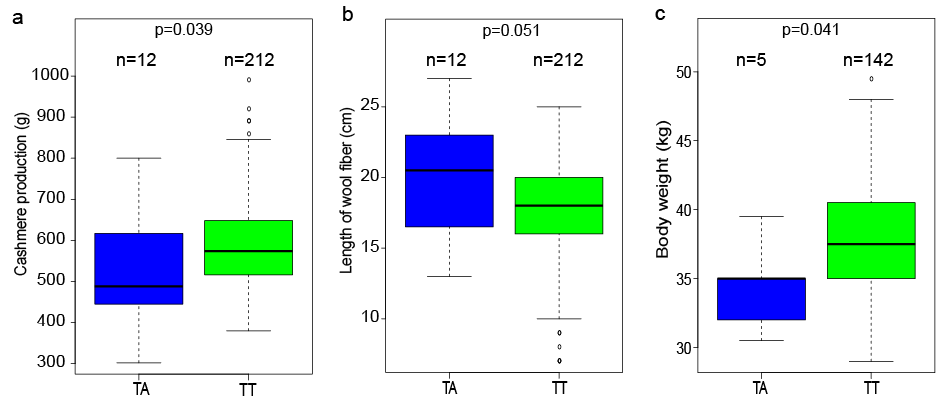


Note: (a) Cashmere production, (b) length of wool fiber, and (c) body weight. Inner Mongolian cashmere goat with economic traits were used in this association study.

# 2. SUPPLEMENTARY TABLES

## Supplementary Table 1. Summary of samples and sequencing.

| **Group** | **Individual ID** | **Uniquely mapping reads** | **Uniquely mapping bases (Gbp)** | **Mean depth** | **Available Site**  **(>=1x)** | **Mapping region**  **/Available Site (%)** | | |
| --- | --- | --- | --- | --- | --- | --- | --- | --- |
|  |  |  |  |  |  | **>10x** | **>20x** | **>30x** |
|  | SN_1 | 443,905,852 | 66.59 | 28.12 | 2,365,793,104 | 97.26 | 87.54 | 44.08 |
|  | SN_2 | 453,316,914 | 68.00 | 28.73 | 2,366,031,063 | 97.36 | 88.42 | 47.51 |
| Dairy_SN | SN_3 | 453,964,632 | 68.09 | 28.75 | 2,366,038,037 | 97.36 | 88.68 | 47.81 |
|  | SN_4 | 437,087,067 | 65.56 | 27.68 | 2,364,896,167 | 97.15 | 86.70 | 41.54 |
|  | SN_5 | 490,474,591 | 73.57 | 31.52 | 2,366,513,273 | 97.79 | 92.30 | 62.96 |
|  |  |  |  |  |  |  |  |  |
|  | LN_1 | 421,032,085 | 63.15 | 26.71 | 2,365,952,146 | 96.99 | 84.22 | 35.92 |
|  | LN_2 | 457,459,448 | 68.62 | 29.01 | 2,366,356,530 | 97.36 | 88.42 | 49.16 |
| Cashmere_LN | LN_3 | 488,410,838 | 73.26 | 30.98 | 2,366,805,849 | 97.61 | 91.11 | 59.87 |
|  | LN_4 | 409,628,033 | 61.44 | 25.99 | 2,365,463,627 | 96.83 | 82.18 | 31.73 |
|  | LN_5 | 467,870,364 | 70.18 | 29.62 | 2,366,421,567 | 97.50 | 89.86 | 52.56 |
|  |  |  |  |  |  |  |  |  |
|  | LZ_1 | 422,931,065 | 63.44 | 27.26 | 2,365,132,287 | 97.17 | 86.29 | 38.67 |
|  | LZ_2 | 475,223,862 | 71.28 | 30.60 | 2,365,999,175 | 97.60 | 91.07 | 57.90 |
| Meat_LZ | LZ_3 | 488,511,003 | 73.28 | 31.47 | 2,366,074,062 | 97.71 | 91.91 | 62.39 |
|  | LZ_4 | 432,761,783 | 64.91 | 27.88 | 2,364,881,588 | 97.25 | 87.33 | 42.49 |
|  | LZ_5 | 440,097,171 | 66.01 | 28.34 | 2,365,638,897 | 97.35 | 88.11 | 45.08 |
|  |  |  |  |  |  |  |  |  |
| Markhor | MK_1 | 440,962,023 | 66.14 | 28.37 | 2,363,604,803 | 96.30 | 83.50 | 47.59 |
|  | MK_2 | 395,111,431 | 59.27 | 25.41 | 2,362,672,679 | 95.39 | 78.21 | 31.61 |
|  |  |  |  |  |  |  |  |  |
| Sindh ibex | SI_1 | 440,160,757 | 66.02 | 27.92 | 2,364,443,130 | 96.45 | 82.73 | 34.73 |
|  | SI_2 | 518,064,525 | 77.71 | 32.85 | 2,365,504,438 | 97.43 | 89.39 | 61.92 |

## Supplementary Table 2.Non-synonymous/synonymous SNP ratios across groups.

| **Group** | **Sample size** | **Coding region SNP** | | | | **Genome Total SNP (M)** |
| --- | --- | --- | --- | --- | --- | --- |
|  |  | **Total SNP(k)** | **Nonsyn SNP(k)** | **Syn SNP(k)** | **NS/S** |  |
| **Domestic** | 15 | 129.07 | 59.73 | 69.34 | 0.86 | 19.04 |
| Dairy_SN | 5 | 77.30 | 34.93 | 42.37 | 0.82 | 11.38 |
| Cashmere_LN | 5 | 81.77 | 37.16 | 44.60 | 0.83 | 12.33 |
| Meat_LZ | 5 | 62.43 | 28.75 | 33.68 | 0.85 | 9.19 |
|  |  |  |  |  |  |  |
| **Wild** | 4 | 87.52 | 43.03 | 44.49 | 0.97 | 11.66 |
| Markhor | 2 | 32.48 | 15.14 | 17.34 | 0.87 | 4.42 |
| Sindh ibex | 2 | 60.14 | 30.91 | 29.23 | 1.06 | 7.63 |
|  |  |  |  |  |  |  |
| **Total** | 19 | 176.14 | 85.72 | 90.42 | 0.95 | 23.92 |

## Supplementary Table 3. Heterozygosity in each individual.

| **Group** | **Individual ID** | **Autosome Heterozygous SNP (M)** | **Heterozygosity（‰）** | **NS/S** | **Non-syn SNP** | **Syn SNP** |
| --- | --- | --- | --- | --- | --- | --- |
|  | SN_1 | 4.04 | 1.89 | 0.83 | 12,869 | 15,433 |
|  | SN_2 | 4.21 | 1.97 | 0.82 | 13,303 | 16,143 |
| Dairy_SN | SN_3 | 3.98 | 1.86 | 0.85 | 13,341 | 15,658 |
|  | SN_4 | 3.87 | 1.81 | 0.84 | 12,382 | 14,757 |
|  | SN_5 | 4.58 | 2.14 | 0.83 | 14,472 | 17,481 |
|  |  |  |  |  |  |  |
|  | LN_1 | 4.31 | 2.01 | 0.83 | 13,501 | 16,173 |
|  | LN_2 | 4.34 | 2.03 | 0.84 | 13,787 | 16,428 |
| Cashmere_LN | LN_3 | 4.34 | 2.03 | 0.84 | 13,669 | 16,311 |
|  | LN_4 | 3.96 | 1.85 | 0.83 | 12,533 | 15,182 |
|  | LN_5 | 4.33 | 2.02 | 0.82 | 13,762 | 16,830 |
|  |  |  |  |  |  |  |
|  | LZ_1 | 3.50 | 1.64 | 0.86 | 11,679 | 13,505 |
|  | LZ_2 | 3.53 | 1.65 | 0.88 | 11,686 | 13,333 |
| Meat_LZ | LZ_3 | 3.57 | 1.67 | 0.87 | 12,036 | 13,829 |
|  | LZ_4 | 2.43 | 1.14 | 0.88 | 8,743 | 9,893 |
|  | LZ_5 | 3.57 | 1.67 | 0.85 | 11,812 | 13,823 |
|  |  |  |  |  |  |  |
| Markhor | MK_1 | 4.29 | 1.98 | 0.85 | 14,380 | 16,940 |
|  | MK_2 | 4.22 | 1.95 | 0.84 | 14,026 | 16,652 |
|  |  |  |  |  |  |  |
| Sindh ibex | SI_1 | 4.14 | 1.92 | 1.08 | 17,997 | 16,711 |
|  | SI_2 | 4.24 | 1.96 | 1.03 | 17,098 | 16,566 |

## Supplementary Table 4. Distribution of breed-specific indels.

| **Group** | **Total Indel(#)** | **Indel in CDS(#)** | **Non-3 bp Indel in CDS(#)** |
| --- | --- | --- | --- |
| Domestication specific | 924,352 | 1,148 | 1,148 |
| Dairy_SN specific | 247,924 | 334 | 334 |
| Cashmere_LN specific | 200,744 | 270 | 270 |
| Meat_LZ specific | 122,293 | 154 | 154 |

## Supplementary Table 5. Genes with frame-shift of the domestic breeds (specific≥60% mutation rate).

| **#Indel_pos** | **Indel_bp (Alt-Ref)** | **Mutation Frequency** | **Gene** |
| --- | --- | --- | --- |
| **Domestic** |  |  |  |
| chrX_9046789 | 4 | 100 | *CSTF2* |
| chrX_121778379 | 5 | 100 | *WWC3* |
| chrX_110195018 | -2 | 93.33 | *goat_GLEAN_10017026* |
| chrX_18123805 | 1 | 86.67 | *AMOT* |
| chr5_108657062 | 1 | 70 | *goat_GLEAN_10003527* |
| chr7_874437 | -1 | 66.67 | *goat_GLEAN_10007872* |
| chr13_35086384 | -2 | 66.67 | *goat_GLEAN_10009178* |
| chr6_23929076 | -2 | 63.33 | *EIF4E* |
| chrX_82112649 | 1 | 60 | *goat_GLEAN_10007947* |
| chrX_28200725 | -1 | 60 | *goat_GLEAN_10020317* |
| chr13_51488176 | 2 | 60 | *SIRPA* |
| chr11_48859195 | -1 | 60 | *MAT2A* |
|  |  |  |  |
| **Dairy_SN** |  |  |  |
| chr22_48123983 | 5 | 90 | *NT5DC2* |
| chr15_77965180 | 1 | 90 | *GOAT_ENSBTAP00000047933-D2* |
| chr1_114066073 | -2 | 90 | *AADAC* |
| chr20_70660165 | 1 | 80 | *BRD9* |
| chr3_111428209 | 1 | 70 | *goat_GLEAN_10010624* |
| chr14_47553160 | -1 | 70 | *STAU2* |
| chrX_7707119 | -1 | 60 | *goat_GLEAN_10017902* |
| chr15_73708811 | -2 | 60 | *goat_GLEAN_10013132* |
| chr15_46452980 | -1 | 60 | *goat_GLEAN_10015100* |
| chr11_37459821 | 1 | 60 | *PRORSD1* |
|  |  |  |  |
| **Cashmere_LN** |  |  |  |
| chr4_46756125 | -1 | 70 | *goat_GLEAN_10012247* |
|  |  |  |  |
| **Meat_LZ** |  |  |  |
| chrX_118838393 | -1 | 90 | *goat_GLEAN_10004717* |
| chr2_2483381 | -1 | 90 | *goat_GLEAN_10013077* |
| chr15_7318653 | -1 | 90 | *goat_GLEAN_10015698* |
| chr10_61569910 | -1 | 90 | *goat_GLEAN_10005895* |
| chr8_109962369 | 2 | 60 | *MYT1L* |

Note: The highlighted genes (pink) discussed in the main paper; the highlighted genes (bold type) discussed in the supplementary discussion.

## Supplementary Table 6. CNV distribution in each individual.

| **Group** | **Individual_ID** | **autosome CNV(#)** | **autosome CNV_length(bp)** |
| --- | --- | --- | --- |
|  | SN_1 | 859 | 12,857,994 |
|  | SN_2 | 653 | 11,905,994 |
| Dairy_SN | SN_3 | 845 | 12,320,632 |
|  | SN_4 | 814 | 12,868,731 |
|  | SN_5 | 619 | 13,292,882 |
|  |  |  |  |
|  | LN_1 | 704 | 12,028,250 |
|  | LN_2 | 747 | 12,854,227 |
| Cashmere_LN | LN_3 | 858 | 12,200,632 |
|  | LN_4 | 427 | 10,393,948 |
|  | LN_5 | 554 | 10,685,047 |
|  |  |  |  |
|  | LZ_1 | 1,185 | 13,785,948 |
|  | LZ_2 | 840 | 12,482,198 |
| Meat_LZ | LZ_3 | 1,218 | 14,056,650 |
|  | LZ_4 | 1,094 | 13,234,797 |
|  | LZ_5 | 1,288 | 14,682,950 |
|  |  |  |  |
| Markhor | MK_1 | 668 | 11,291,062 |
|  | MK_2 | 1,228 | 14,427,062 |
|  |  |  |  |
| Sindh ibex | SI_1 | 1,003 | 12,116,812 |
|  | SI_2 | 1,228 | 13,401,632 |

## Supplementary Table 7. CNV associated genes of domestic breeds (≥60% frequency).

| **Gene** | **Copy Number of each individual** | | | |
| --- | --- | --- | --- | --- |
|  | **Dairy_SN** | **Cashmere_LN** | **Meat_LZ** | **Wild** |
| **Domestic specific** |  |  |  |  |
| GOAT_ENSBTAP00000041556-D40 | 5 3 3 4 5 | 3 3 3 2 2 | 2 4 4 4 2 | 2 2 2 2 |
| goat_GLEAN_10007289 | 2 1 1 0 2 | 1 1 1 2 2 | 0 1 1 0 1 | 2 2 2 2 |
| goat_GLEAN_10007290 | 2 1 1 0 2 | 1 1 1 2 2 | 0 1 1 0 1 | 2 2 2 2 |
| **ASIP** | 6 6 6 6 6 | 4 4 4 4 4 | 2 2 2 2 2 | 2 2 2 2 |
| **AHCY** | 6 6 6 6 6 | 4 4 4 4 4 | 2 2 2 2 2 | 2 2 2 2 |
| GOAT_ENSBTAP00000048236-D2 | 2 2 4 2 2 | 4 2 9 5 4 | 8 7 4 2 7 | 2 2 2 2 |
|  |  |  |  |  |
| Dairy_SN specific |  |  |  |  |
| GOAT_ENSBTAP00000049298-D3 | 1 1 1 2 1 | 2 2 2 2 2 | 2 2 2 2 2 | 2 2 2 2 |
| TRBV27 | 1 1 1 2 1 | 2 2 2 2 2 | 2 2 2 2 2 | 2 2 2 2 |
| GOAT_ENSP00000374922-D4 | 1 1 1 2 1 | 2 2 2 2 2 | 2 2 2 2 2 | 2 2 2 2 |
| TRBC2 | 1 1 1 2 1 | 2 2 2 2 2 | 2 2 2 2 2 | 2 2 2 2 |
| goat_GLEAN_10015246 | 1 1 1 2 1 | 2 2 2 2 2 | 2 2 2 2 2 | 2 2 2 2 |
| goat_GLEAN_10015247 | 1 1 1 2 1 | 2 2 2 2 2 | 2 2 2 2 2 | 2 2 2 2 |
| goat_GLEAN_10020568 | 3 2 4 3 3 | 2 2 2 2 2 | 2 2 2 2 2 | 2 2 2 2 |
| GOAT_ENSBTAP00000001728-D3 | 2 2 1 0 1 | 2 2 2 2 2 | 2 2 2 2 2 | 2 2 2 2 |
|  |  |  |  |  |
| Cashmere_LN specific |  |  |  |  |
| GOAT_ENSBTAP00000044328 | 2 2 2 2 2 | 1 1 0 1 2 | 2 2 2 2 2 | 2 2 2 2 |
| GOAT_ENSBTAP00000044328-D11 | 2 2 2 2 2 | 1 1 0 1 2 | 2 2 2 2 2 | 2 2 2 2 |
| GOAT_ENSBTAP00000048661-D6 | 2 2 2 2 2 | 1 1 0 1 2 | 2 2 2 2 2 | 2 2 2 2 |
| GOAT_ENSBTAP00000049767 | 2 2 2 2 2 | 1 0 2 1 0 | 2 2 2 2 2 | 2 2 2 2 |
| goat_GLEAN_10003709 | 2 2 2 2 2 | 1 0 2 1 0 | 2 2 2 2 2 | 2 2 2 2 |
| GOAT_ENSBTAP00000048661-D16 | 2 2 2 2 2 | 2 1 0 1 2 | 2 2 2 2 2 | 2 2 2 2 |
|  |  |  |  |  |
| **Meat_LZ specific** |  |  |  |  |
| *goat_GLEAN_10014190* | 2 2 2 2 2 | 2 2 2 2 2 | 0 1 2 0 0 | 2 2 2 2 |
| *PKDREJ* | 2 2 2 2 2 | 2 2 2 2 2 | 2 2 1 1 1 | 2 2 2 2 |
|  |  |  |  |  |

Note：Data with pink shadow indicate specific CNV.

## Supplementary Table 8. CNV association with economic trait in the dairy breeds.

| **Gene** | **Trait** | **n** | **P-value** | **Core** |
| --- | --- | --- | --- | --- |
| *chr5: APOL3* | Average milk lactose content | 126 | 0.013 | 0.22 |
|  | Morning milk lactose content | 125 | ***0.009*** | 0.232 |
|  | Morning milk density content | 125 | 0.045 | 0.18 |
|  | Night milk lactose content | 126 | 0.021 | 0.205 |
|  |  |  |  |  |
| *chr22: NME6* | Average milk solids-not-fat content | 144 | 0.016 | 0.2 |
|  | Average milk lactose content | 144 | 0.019 | 0.195 |
|  | Average milk density content | 144 | 0.024 | 0.188 |
|  | Morning milk solids-not-fat content | 143 | 0.02 | 0.195 |
|  | Morning milk lactose content | 143 | 0.031 | 0.181 |
|  | Morning milk density content | 143 | 0.045 | 0.168 |
|  | Night milk solids-not-fat content | 144 | 0.014 | 0.205 |
|  | Night milk lactose content | 144 | 0.013 | 0.206 |
|  | Night milk density content | 144 | 0.012 | 0.208 |
|  | Night milk acidity content | 144 | 0.038 | 0.173 |
|  | Hip width | 60 | 0.03 | 0.281 |
|  | Chest width | 60 | 0.04 | -0.265 |
|  | Waist height | 60 | ***0.001*** | -0.43 |

## Supplementary Table 9. The distribution of genes in SNP deserts.

| **Group** | **Desert region (Mb)** | **Gene number in** | |  | **QTL gene number in** | | |
| --- | --- | --- | --- | --- | --- | --- | --- |
|  |  | **genome** | **desert** |  | **genome** | **desert** | **chisq.test** |
| Dairy_SN | 277.39 | 21,389 | 3,950 |  | 3,709 | 661 | p=0.4487 |
| Cashmere_LN | 278.33 | 21,389 | 4,395 |  | 4,454 | 915 | p=1 |
| Meat_LZ | 273.00 | 21,389 | 3,447 |  | 11,581 | 1,845 | p=0.7225 |

## Supplementary Table 10. GO enrichment of the breed-shared SAGs.

| **GO_Term** | **GO levl** | **GO_Class** | **Adjusted Pv** | **x1** | **x2** | **n** | **N** | **Enrich Direct** |
| --- | --- | --- | --- | --- | --- | --- | --- | --- |
| **all shared 1196 desert genes (only GO level 4 showed)** |  |  |  |  |  |  |  |  |
| regulation of cellular metabolic process | 4 | BP | 9.99E-11 | 108 | 940 | 853 | 14,121 | Over |
| regulation of primary metabolic process | 4 | BP | 1.18E-10 | 107 | 933 | 853 | 14,121 | Over |
| regulation of macromolecule metabolic process | 4 | BP | 1.39E-10 | 102 | 879 | 853 | 14,121 | Over |
| nucleic acid binding | 4 | MF | 3.15E-08 | 166 | 1,779 | 853 | 14,121 | Over |
| intracellular membrane-bounded organelle | 4 | CC | 3.65E-07 | 122 | 1,248 | 853 | 14,121 | Over |
| cellular nitrogen compound metabolic process | 4 | BP | 5.92E-06 | 160 | 1,822 | 853 | 14,121 | Over |
| nucleobase-containing compound metabolic process | 4 | BP | 6.16E-06 | 156 | 1,768 | 853 | 14,121 | Over |
| cellular aromatic compound metabolic process | 4 | BP | 6.25E-06 | 159 | 1,811 | 853 | 14,121 | Over |
| heterocycle metabolic process | 4 | BP | 6.25E-06 | 159 | 1,811 | 853 | 14,121 | Over |
| organic cyclic compound metabolic process | 4 | BP | 1.72E-05 | 159 | 1,838 | 853 | 14,121 | Over |
| cellular macromolecule metabolic process | 4 | BP | 2.79E-05 | 230 | 2,877 | 853 | 14,121 | Over |
| macromolecule metabolic process | 4 | BP | 0.0004 | 253 | 3,328 | 853 | 14,121 | Over |
| cation binding | 4 | MF | 0.0016 | 173 | 2,191 | 853 | 14,121 | Over |
| cellular biosynthetic process | 4 | BP | 0.0028 | 142 | 1,761 | 853 | 14,121 | Over |
| regulation of cell cycle | 4 | BP | 0.0028 | 9 | 35 | 853 | 14,121 | Over |
| organic substance biosynthetic process | 4 | BP | 0.0035 | 143 | 1,784 | 853 | 14,121 | Over |
| transcription cofactor activity | 4 | MF | 0.0198 | 8 | 37 | 853 | 14,121 | Over |
|  |  |  |  |  |  |  |  |  |
| **shared 231 genes in >100 k desert** |  |  |  |  |  |  |  |  |
| cell part | 2 | CC | 0.0290 | 68 | 3,761 | 176 | 14,121 | Over |
| cell periphery | 3 | CC | 0.0002 | 16 | 298 | 176 | 14,121 | Over |
| plasma membrane | 3 | CC | 0.0005 | 15 | 285 | 176 | 14,121 | Over |
| cell adhesion | 3 | BP | 0.0034 | 11 | 194 | 176 | 14,121 | Over |
| homophilic cell adhesion | 5 | BP | 2.76E-05 | 10 | 78 | 176 | 14,121 | Over |
| guanyl-nucleotide exchange factor activity | 5 | MF | 0.0334 | 7 | 119 | 176 | 14,121 | Over |
| protein methyltransferase activity | 6 | MF | 0.0261 | 4 | 26 | 176 | 14,121 | Over |
| ARF guanyl-nucleotide exchange factor activity | 6 | MF | 0.0313 | 3 | 14 | 176 | 14,121 | Over |
| Ras protein signal transduction | 7 | BP | 0.0305 | 6 | 81 | 176 | 14,121 | Over |
| ARF protein signal transduction | 8 | BP | 0.0313 | 3 | 14 | 176 | 14,121 | Over |

## Supplementary Table 11. SAGs in the top 10 larger SNP deserts of the cashmere breed.

| **Chr** | **Start** | **End** | **Size**  **(kb)** | **Gene#** | **Gene list** |
| --- | --- | --- | --- | --- | --- |
| chrX | 59,940,000 | 62,360,000 | 2,420 | 9 | *goat_GLEAN_10020908, goat_GLEAN_10020907, goat_GLEAN_10020906, goat_GLEAN_10020905, goat_GLEAN_10020904, RPS6KA6, goat_GLEAN_10020902, POU3F4, SNRPE* |
| chrX | 63,100,000 | 64,890,000 | 1,790 | 5 | *goat_GLEAN_10020899, SH3BGRL, HMGN5, goat_GLEAN_10020897, BRWD3* |
| chrX | 58,720,000 | 59,910,000 | 1,190 | 5 | *CHM, goat_GLEAN_10020912, POF1B, ZNF711, GOAT_ENSP00000362264* |
| chr26 | 20,430,000 | 21,580,000 | 1,150 | 25 | *BTRC, goat_GLEAN_10018443, POLL, DPCD, GOAT_ENSP00000373698, FGF8, NPM3 MGEA5, KCNIP2, C10orf76 ,HPS6, RPL32, LDB1, PPRC1, NOLC1, ELOVL3PITX3GBF1, NFKB2PSD, FBXL15, CUEDC2, TMEM180, ACTR1A SUFU* |
| chrX | 84,450,000 | 85,500,000 | 1,050 | 14 | *GPR173, TSPYL2, GOAT_ENSBTAP00000051529, KDM5C, goat_GLEAN_10001514, goat_GLEAN_10001515, SMC1, ARIBC1, HSD17B10, HUWE1, GOAT_ENSBTAP00000049034, goat_GLEAN_10004217 PHF8, goat_GLEAN_10005150* |
| chr8 | 34,040,000 | 35,060,000 | 1,020 | 53 | *C16orf70, GOAT_ENSBTAP00000043400, TRADD, FBXL8, HSF4, NOL3; KIAA0895L, EXOC3L1 E2F4, ELMO3, GOAT_ENSBTAP00000043349, TMEM208, FHOD1, SLC9A5, PLEKHG4, KCTD19, LRRC36, TPPP3, ZDHHC1*  *HSD11B2, ATP6, V0D1A, GRP, FAM65, ACTCF, RLTPR*  *goat_GLEAN_10013974, PARD6A, ENKD1, C16orf86, GFOD2, RANBP10, TSNAXIP1 CENPT, THAP11, NUTF2, EDC4, GOAT_ENSBTAP00000008418, PSKH1, GOAT_ENSBTAP00000050193 PSMB10, LCAT SLC12A4, DPEP2, DDX28, DUS2, LNFATC3, ESRP2, PLA2G15 GOAT_ENSP00000402156 SLC7A6OS PRMT7, SMPD3, goat_GLEAN_10012845* |
| chrX | 71,690,000 | 72,690,000 | 1,000 | 22 | *goat_GLEAN_10013446 CXCR3 GOAT_ENSBTAP00000028460 OGTTAF1 GOAT_ENSBTAP00000005370, ITGB1BP2, NONO, ZMYM3, GJB1, GOAT_ENSBTAP00000027332 NLGN3, MED12, IL2RG, GOAT_ENSP00000411354-D2, FOXO4, goat_GLEAN_10013462, SNX12 GOAT_ENSBTAP00000049500, SLC7A3, TEX11, DLG3* |
| chrX | 75,700,000 | 76,570,000 | 870 | 2 | *ARGOAT_ENSP00000363822-D2* |
| chrX | 82,730,000 | 83,440,000 | 710 | 2 | *LAS1LZC3H12B* |
| chrX | 67,050,000 | 67,750,000 | 700 | 7 | *GOAT_ENSP00000355170 , goat_GLEAN_10020883 ,COX7B, MAGT1, ATRX, FGF16, goat_GLEAN_10020878* |

Note：Genes in red indicate shared by all domestic breeds.

## Supplementary Table 12. SAGs in the top 10 larger SNP deserts of the dairy breed.

| **Chr** | **Start** | **End** | **Size(kb)** | **Gene#** | **Gene list** |
| --- | --- | --- | --- | --- | --- |
| chrX | 63,100,000 | 65,670,000 | 2,570 | 9 | *goat_GLEAN_10020899, SH3BGRL, HMGN5, goat_GLEAN_10020897, BRWD3, FAM46D, goat_GLEAN_10020894, TBX22, goat_GLEAN_10020892* |
| chrX | 59,940,000 | 62,360,000 | 2,420 | 9 | *goat_GLEAN_10020908, goat_GLEAN_10020907, goat_GLEAN_10020906, goat_GLEAN_10020905, goat_GLEAN_10020904, RPS6KA6, goat_GLEAN_10020902, POU3F4, SNRPE* |
| chrX | 65,690,000 | 67,740,000 | 2,050 | 15 | *GPR174, P2RY10, LPAR4, goat_GLEAN_10020888, GOAT_ENSBTAP00000036753, TAF9B, PGK1, goat_GLEAN_10020885, GOAT_ENSP00000355170 goat_GLEAN_10020883, COX7B, MAGT1, ATRX, FGF16, goat_GLEAN_10020878* |
| chr9 | 67,090,000 | 68,870,000 | 1,780 | 13 | *GOAT_ENSP00000275235-D2 goat_GLEAN_10006376, ADAT2, PEX3, FUCA2 goat_GLEAN_10006380, PHACTR2, LTV1, ZC2HC1B GOAT_ENSP00000388045 SF3B5, STX11, UTRN* |
| chrX | 82,180,000 | 83,440,000 | 1,260 | 6 | *VSIG4, goat_GLEAN_10008085, MSN goat_GLEAN_10008087, LAS1LZC3H12B* |
| chrX | 58,720,000 | 59,920,000 | 1,200 | 5 | *CHM, goat_GLEAN_10020912, POF1B, ZNF711, GOAT_ENSP00000362264* |
| chrX | 68,900,000 | 70,090,000 | 1,190 | 9 | *ABCB7, goat_GLEAN_10006428, KIAA2022, RLIM SLC16A2, goat_GLEAN_10006424 goat_GLEAN_10006423, ZCCHC13, goat_GLEAN_10006422* |
| chrX | 75,700,000 | 76,500,000 | 800 | 2 | *AR, GOAT_ENSP00000363822-D2* |
| chr2 | 35,910,000 | 36,550,000 | 640 | 6 | *LY75-CD302, MARCH7, goat_GLEAN_10020517, BAZ2B, WDSUB1, TANC1* |
| chr17 | 34,140,000 | 34,680,000 | 540 | 3 | *SPATA5, NUDT6, FGF2* |

Note：Genes in red indicate shared by all domestic breeds.

## Supplementary Table 13. SAGs in the top 10 larger SNP deserts of the meat breed.

| **Chr** | **Start** | **End** | **Size(kb)** | **Gene#** | **Gene list** |
| --- | --- | --- | --- | --- | --- |
| chrX | 74,400,000 | 76,850,000 | 2,450 | 4 | *YIPF6, GOAT_ENSP00000347710-D2, AR, GOAT_ENSP00000363822-D2* |
| chrX | 85,360,000 | 86,270,000 | 910 | 9 | *PHF8, goat_GLEAN_10005150, FAM120, CWNK3, TSR2, FGD1, GNL3L, goat_GLEAN_10000688, MAGED2* |
| chr12 | 24,550,000 | 25,430,000 | 880 | 3 | *goat_GLEAN_10020469, NBEA, MAB21L1* |
| chrX | 80,240,000 | 80,850,000 | 610 | 3 | *EDA2R, goat_GLEAN_10003552, goat_GLEAN_10003551* |
| chr12 | 34,670,000 | 35,150,000 | 480 | 7 | *IFT88, GOAT_ENSBTAP00000051202, GJB6 GJB2GJA3, GOAT_ENSP00000413744, PSPC1* |
| chrX | 31,340,000 | 31,820,000 | 480 | 4 | *goat_GLEAN_10020331, goat_GLEAN_10020332, XIAP, STAG2* |
| chr16 | 42,120,000 | 42,580,000 | 460 | 2 | *GOAT_ENSBTAP00000017839, RERE* |
| chr16 | 53,090,000 | 53,530,000 | 440 | 1 | *RABGAP1L* |
| chr7 | 65,440,000 | 65,870,000 | 430 | 5 | *CLINT1, GOAT_ENSBTAP00000051274 goat_GLEAN_10016376, goat_GLEAN_10016375, goat_GLEAN_10016374* |
| chr9 | 42,270,000 | 42,700,000 | 430 | 1 | *GOAT_ENSBTAP00000044216* |

Note：Genes in red indicate shared by all domestic breeds.

## Supplementary Table 14. A list of genes overlapping between DAGs and SAGs.

|  | **SAG** | | | **Gene Description** | **Function** | |
| --- | --- | --- | --- | --- | --- | --- |
| **DAG** | **Dairy**  **_SN** | **Cashmere**  **_LN** | **Meat**  **_LZ** |
| ● | ● | ● | ● | TNFSF13 (tumor necrosis factor (ligand) superfamily, member 13) | | Associated with immunodeficiency  [1] |
| ● | ● | ● | ● | *SENP3 (*sentrin-specific protease 3*)* | |  |
| ● | ● | ● | ● | *CD68 (*CD68 antigen*)* | |  |
| ● | ● | ● | ● | TP53 (tumor protein p53) | | Candidate for muscle development[2] |
| ● | ● | ● | ● | EFNB3 (ephrin-B3) | |  |
| ● | ● | ● | ● | *STAM* (signal transducing adaptor molecule) | |  |
| ● | ● | ● | ● | *DNAH2 (*dynein heavy chain, axonemal*)* | |  |
| ● | ● | ● | ● | SOX15 (transcription factor SOX, other) | | Associated with skeletal muscle regeneration[3] |
| ● | ● | ● | ● | FXR2 (Fragile X Mental Retardation, Autosomal Homolog 2) | | Fxr2 knockout mice were hyperactive[4] |
| ● | ● | ● | ● | *SCAF1 (*SR-Related CTD-Associated Factor 1) | |  |
| ● | ● | ● | ● | AHRR (aryl hydrocarbon receptor repressor) | |  |
| ● | ● | ● | ● | RRM1 (ribonucleoside-diphosphate reductase subunit M1) | |  |
| ● | ● | ● | ● | ATP1B2 (sodium/potassium-transporting ATPase subunit beta) | | ATP1B1 is candidate gene for response to hypoxia in pig[5] |
| ● | ● | ● | ● | *CYB5D1 (*Cytochrome B5 Domain Containing 1*)* | |  |
| ● | ● | ● | ● | *CYSTM1 (*Cysteine-Rich Transmembrane Module Containing 1 *)* | |  |
| ● | ● | ● | ● | *PFDN1 (*prefoldin subunit 1*)* | |  |
| ● | ● | ● | ● | *RRAS (*Ras-related protein R-Ras*)* | |  |
| ● | ● | ● | ● | *IRF3*(interferon regulatory factor 3*)* | |  |
| ● | ● | ● | ● | *GOAT_ENSBTAP00000041460* | |  |
| ● | ● | ● | ● | NR6A1 (germ cell nuclear factor) | | Influence vertebrae number[6] |
| ● | ● | ● | ● | *BCL2L12 (*BCL2-Like 12*)* | |  |
| ● | ● | ● | ● | KDM6B (Lysine (K)-Specific Demethylase 6B) | | Under selection in rabbit[7] |
| ● | ● | ● | ● | *RANBP6 (*RAN Binding Protein 6*)* | |  |
| ● | ● | ● | ● | *EIF4A1 (*Eukaryotic Translation Initiation Factor 4A1*)* | |  |
| ● | ● | ● | ● | *NAB2 (*  NGFI-A Binding Protein 2 (EGR1 Binding Protein 2)*)* | |  |
| ● | ● | ● | ● | *STAT6 (*signal transducer and activator of transcription 6*)* | | Association with carcass traits in cattle[8] |
| ● | ● | ● | ● | STIM1 (Stromal Interaction Molecule 1) | | Associated with immune regulation  [9] |
| ● | ● | ● | ● | *TMEM88 (*Transmembrane Protein 88*)* | |  |
| ● | ● | ● | ● | *HARBI1 (*Harbinger Transposase Derived *)* | |  |
| ● | ● | ● | ● | *EXOC3 (*exocyst complex component 3*)* | |  |
| ● | ● | ● | ● | *IGIP (*IgA-Inducing Protein*)* | |  |
| ● | ● | ● | ● | *LSMD1 (*small nuclear ribonucleoprotein B and B'*)* | |  |
| ● | ● | ● | ● | *PURA (*Purine-Rich Element Binding Protein A*)* | |  |
| ● | ● | ● | ● | *MPDU1 (*mannose-P-dolichol utilization defect 1*)* | |  |
| ● | ● | ● | ● | *SHBG (*Sex Hormone-Binding Globulin*)* | |  |
| ● | ● | ● | ● | *GOAT_ENSP00000369833* | |  |
| ● | ● | ● | ● | *GOAT_ENSP00000378079* | |  |
| ● | ● | ● | ● | *KIAA2026* | |  |
| ● | ● | ● | ● | *RERE (*arginine-glutamic acid dipeptide (RE) repeats*)* | |  |
| ● | ● | ● | ● | *goat_GLEAN_10018912 ()* | |  |
| ● | ○ | ● | ● | *RHOG (*Ras homolog gene family, member G*)* | | promotes neural progenitor cell proliferation[10] |
| ● | ○ | ● | ● | SHOC2 (Soc-2 Suppressor Of Clear Homolog) | | regulates neurite outgrowth[11] |
| ● | ○ | ● | ● | *LRP1* (low density lipoprotein-related protein 1) | | Associated with lipid metabolism[12] |
| ● | ○ | ● | ● | *RCN3 (*Reticulocalbin 3, EF-Hand Calcium Binding Domain*)* | |  |
| ● | ○ | ● | ● | *GOAT_ENSBTAP00000039644-D3* | | |
| ● | ○ | ● | ● | *GOAT_ENSBTAP00000051850* | |  |
| ● | ○ | ● | ● | *PRRG2 (*Proline Rich Gla (G-Carboxyglutamic Acid) 2*)* | |  |
| ● | ○ | ● | ● | *ART1 (*ADP-ribosyltransferase 1*)* | |  |
| ● | ○ | ● | ● | *CHRNA10 (*nicotinic acetylcholine receptor alpha-10*)* | |  |
| ● | ○ | ● | ● | *PGAP2 (*Post-GPI Attachment To Proteins 2*)* | |  |
| ● | ○ | ● | ● | ADRA2A (adrenergic receptor alpha-2A) | | Associated with personality traits[13] |
| ● | ○ | ● | ● | *TMEM194A (*Transmembrane Protein 194A*)* | |  |
| ● | ○ | ● | ● | *NUP98 (*nuclear pore complex protein Nup98-Nup96*)* | |  |
| ● | ○ | ● | ● | *NR5A1 (*steroidogenic factor 1*)* | |  |
| ● | ○ | ● | ● | *NOSIP (*nitric oxide synthase-interacting protein*)* | |  |
| ● | ○ | ● | ● | *goat_GLEAN_10015165* | |  |
| ● | ● | ● | ○ | *SGOL1 (*shugoshin-like 1*)* | |  |
| ● | ● | ● | ○ | *KIF1B (*Kinesin Family Member 1B *)* | |  |
| ● | ● | ● | ○ | *NUPL1 (*nucleoporin p58/p45*)* | |  |
| ● | ● | ● | ○ | *GOAT_ENSP00000416371* | |  |
| ● | ● | ○ | ○ | *YEATS2 (*YEATS Domain Containing 2*)* | |  |
| ● | ● | ○ | ○ | *NDUFB2 (*NADH Dehydrogenase (Ubiquinone) 1 Beta Subcomplex, 2*)* | |  |
| ● | ○ | ● | ○ | *PCAF (*histone acetyltransferase*)* | |  |
| ● | ○ | ● | ○ | *MTMR6 (*Myotubularin Related Protein 6*)* | |  |
| ● | ○ | ○ | ● | *WRAP53 (*WD Repeat Containing, Antisense To TP53*)* | |  |
| ● | ○ | ○ | ● | *BRAF (*B-Raf proto-oncogene serine/threonine-protein kinase*)* | |  |
| ● | ○ | ○ | ● | *GOAT_ENSP00000318247* | |  |

## Supplementary Table 15. GO enrichment of DAGs.

| **GO term** | **Description** | **P-value** | **FDR q-value** | **Enrichment (N, B, n, b)** | **Genes** |
| --- | --- | --- | --- | --- | --- |
| GO:0000059 | protein import into nucleus, docking | 5.14E-04 | 1.00E+00 | 57.33 (13072,8,57,2) | NUP98 - nucleoporin 98kda |
|  |  |  |  |  | RANBP6 - ran binding protein 6 |
|  |  |  |  |  | PURA - purine-rich element binding protein a |
|  |  |  |  |  | SOX15-sry (sex determining region y)-box 15 |
|  |  |  |  |  | FXR2 - fragile x mental retardation, autosomal homolog 2 |
|  |  |  |  |  | NAB2 - ngfi-a binding protein 2 (egr1 binding protein 2) |
|  |  |  |  |  | STAT6 - signal transducer and activator of transcription 6, interleukin-4 induced |
|  |  |  |  |  |  |
|  |  |  |  |  |  |
| GO:0010629 | negative regulation of gene expression | 7.37E-04 | 1.00E+00 | 2.71 (13072,1101,57,13) | AHRR - aryl-hydrocarbon receptor repressor |
|  |  |  |  |  | TP53 - tumor protein p53 |
|  |  |  |  |  | NR6A1 - nuclear receptor subfamily 6, group a, member 1 |
|  |  |  |  |  | NUP98 - nucleoporin 98kda |
|  |  |  |  |  | BCL2L12 - bcl2-like 12 (proline rich) |
|  |  |  |  |  | RERE - arginine-glutamic acid dipeptide (re) repeats |
|  |  |  |  |  | NUPL1 - nucleoporin like 1 |
|  |  |  |  |  | YEATS2 - yeats domain containing 2 |

Note: GO enrichment analysis of genes residing with a window with ZHp < -3 and ZFst>3.

## Supplementary Table 16. TAGs in chromosomal regions.

| **#Chr** | **Pos** | **ZFst** | **XP-EHH** | **GeneList** |
| --- | --- | --- | --- | --- |
| **Dairy_SN** |  |  |  |  |
| chr2 | 36400000 | 4.29 | 2.58 | BAZ2B, WDSUB1 |
| chr9 | 89800000 | 5.04 | 2.53 | goat_GLEAN_10005927 |
| chr9 | 68600000 | 4.14 | 3.61 | UTRN |
| chr9 | 68700000 | 4.06 | 3.95 | / |
| chr9 | 68100000 | 4.43 | 4.34 | UTRN |
| chr8 | 73600000 | 4.18 | 1.91 | UBAP2, DCAF12 |
| chr10 | 44300000 | 4.98 | 1.84 | **VPS13C** |
|  |  |  |  |  |
| **Cashmere_LN** | |  |  |  |
| chr6 | 92100000 | 5.49 | 2.85 | **FGF5**, goat_GLEAN_10013558 |
| chr6 | 92200000 | 4.62 | 2.73 | / |
| chr7 | 27000000 | 7.04 | 2.15 | **PRDM6** |
| chr8 | 7500000 | 4.43 | 2.32 | goat_GLEAN_10003434, BLK |
| chr8 | 12500000 | 5.74 | 2.72 | / |
| chr8 | 12600000 | 4.03 | 1.95 | / |
| chr13 | 60600000 | 4.38 | 2.15 | BASE, C13H20ORF71, BPIFA1, BPIFB1 |
| chr13 | 62100000 | 4.50 | 2.12 | NCOA6, GGT7 |
| chr13 | 56000000 | 5.59 | 2.38 | RAB22A, goat_GLEAN_10011473, goat_GLEAN_10011472 |
| chr14 | 7900000 | 4.25 | 2.29 | / |
| chr20 | 62000000 | 4.51 | 2.79 | DAP, ANKRD33B |
| chr20 | 62100000 | 4.06 | 3.36 | ANKRD33B |
| chr24 | 43800000 | 4.09 | 3.42 | / |
| chr25 | 1500000 | 4.27 | 2.12 | goat_GLEAN_10004920, RPL3L, NDUFB10, RPS2, RNF151, TBL3, NOXO1, GFER, SYNGR3, ZNF598, goat_ENSP00000191922 |
|  |  |  |  |  |
| **Meat_LZ** |  |  |  |  |
| chr1 | 1.49E+08 | 4.54 | 2.68 | ETS2 |
| chr6 | 78500000 | 4.35 | 2.79 | / |
| chr7 | 57500000 | 4.39 | 2.80 | goat_ENSBTAP00000017199, goat_GLEAN_10018710, **SLC26A2**, **HMGXB3** |
| chr9 | 42400000 | 4.11 | 2.38 | goat_ENSBTAP00000044216 |
| chr9 | 42500000 | 4.08 | 3.54 | / |
| chr13 | 76200000 | 4.99 | 2.83 | TMEM189-UBE2V1 |
| chr15 | 57800000 | 4.43 | 2.47 | MPPED2 |
| chr24 | 58100000 | 5.15 | 2.41 | goat_ENSBTAP00000030954, goat_GLEAN_10002842, SEC11C, goat_GLEAN_10002840, GRP |
| chr28 | 3700000 | 4.20 | 2.74 | / |
| chr28 | 7400000 | 4.13 | 2.75 | HEATR1 |

# 3. SUPPLEMENTARY DISCUSSION

## 3.1 SNP-desert-associated genes (SAGs)

We examined the large SNP deserts that are more than 100 kb in length as well as the top 10 larger deserts unique to each domestic breed. For the top 10 SNP deserts found in each breed, the cashmere breed does not have any unique loci and all have shared SAGs with other breeds (Supplementary Table 11). On the contrary, the meat and dairy breeds both have unique loci to their own, four for the meat and two for the dairy.

The dairy breed has two unique SNP deserts, one on chromosome 9 and the other on chromosome 17 (Supplementary Table 12). The *SPATA5-**NUDT6-**FGF2* desert on chromosome 17 is a region of 540 kb. *FGF2* (fibroblast growth factor 2), which is a member of the fibroblast growth factor (FGF) family, is reported to control mammary ductal elongation when the epithelium proliferates **[14]**. The large chromosome 9 desert is composed of imprinted genes **[15]**.

The meat breed-associated large SNP deserts genes are: *XIAP-STAG2* of X chromosome, *RABGAP1L,* and *CLINT1* (Supplementary Table 13). *XIAP* (X-linked inhibitor of apoptosis) gene play a role in innate immunity by the finding that *XIAP* is involved in the function of Dectin-1[16]*. STAG2* (stromal antigen 2) encodes a component of the cohesin complex, its micro duplication may be responsible for the specific clinical findings in the human cohesinopathy **[17]**. A deletion of *RABGAP1L* (RAB GTPase activating protein 1-Like) is associated with the risk of systemic lupus erythematosus **[18]**. And *CLINT1*, also known as epsinR and epsin4, is associated with schizophrenia and psychotic disorders **[19]**.

## 3.2 Domestication-associated genes (DAGs)

Strong trait-driven selection often leads to selective sweeps with conditional beneficial variants, together with adjacent neutral sequences, resulting in reduced heterozygosity and increased differentiation between populations around the selected sites **[7]**. To detect the sequence signature of selective sweeps over large genomic regions, we defined 67 domestication-associated genes (DAGs) in a collective genomic length of 3.2 Mb (Fig.3).

This set of genes may contribute to behavior, immunity, and morphological differences between domestic and wild goats. First, genes directly influencing nervous system and behavior include *ADRA2A,* and *FXR2*. For instance, *ADRA2A* (alpha-2-adrenergic receptors) is a G protein-coupled receptor that regulate neurotransmitter release, and an *ADRA2A* mutation exhibits personality traits in adolescents **[13]**; *FXR2* (fragile X mental retardation, autosomal homolog 2), is a RNA binding protein containing two KH domains and one RCG box. It is reported that *FXR2* is required for the presence of behavioral circadian rhythms and *FXR2* knockout mice were hyperactive **[20]**. Second, *TNFSF13* (tumor necrosis factor (ligand) superfamily, member 13) and *STIM1* (stromal interaction molecule 1) regulate B-cell development **[21]** and T cell-mediated immune regulation during chronic infection **[9]**, respectively. Third, morphological difference involves several genes; *NR6A1* (germ cell nuclear factor), a member of the nuclear hormone receptor family, is suggested to be a candidate for QTL that affect the number of vertebrates **[6]**, as one of the most characteristic morphological changes in domestic pigs **[22]**. *LRP1* (low-density lipoprotein receptor-related protein 1) is a large cell surface receptor, critical in lipid metabolism through regulating leptin signaling and energy homeostasis, and regulates food intake and energy homeostasis in the adult central nervous system **[12]**. *STAT6,* is a member of the STAT family of transcription factors, acts as a mediator of leptin signaling and has been associated with body weight as well as carcass and growth efficiency traits**[8]**. Another important gene, *SOX15* (sex determining region Y-Box 15), is shown to be involved in regulating cell fate in embryos**[3]**. All these genes are appealing candidates for further investigation.

In addition, we detected two frame-shifts showing as high frequency indels in domestic goats. One involves *EIF4E* (eukaryotic translation initiation factor 4E), a gene associated with repetitive behaviors in human **[23]**, and the other, *SIRPA* (signal-regulatory protein alpha) is a member of the immunoglobulin superfamily, whose polymorphisms are found to associate with increased risk of oligozoospermia **[24]** (Supplementary table 5).

# 4. SUPPLEMENTARY METHODS

## 4.1 Samples

A total of 19 individuals represent three domestic breeds, 5 individuals from each, the dairy breed Saanen, the cashmere breed Liaoning, and the meat breed Leizhou, as well as 4 wild goats, two of Markhor and two of Sindh ibex. The Saanen goat is the most popular dairy breed that is originated in Switzerland, highly selected for milk production (white/cream coat). The Liaoning cashmere goat has been bred in Liaoning province, known for its high-yield cashmere (white to cream coat). The Leizhou goat is a native Chinese breed, used for meat production, sampled from Guangdong province (solid black and a few with solid brown). Markhor (Capra falconeri) is a wild goat distribution in Pakistan, India, Tajikistan, Afghanistan, Turkmenistan and Uzbekistan. It is categorized as Endangered on the IUCN Red List (<http://www.iucnredlist.org/details/3787/0>) and is probably the origin of domesticated goat breeds. Sindh ibex (Capra aegagrus blythi) from Pakistan is rather stocky animals with thick-set body and strong limbs terminating in broad hooves. Skin biopsies or blood samples were collected and genomic DNA was extracted by using Omega Tissue & Blood Kit (Omega Bio-tek, Norcross, GA, USA).

| **Sample_name** | **Organism** | **Breed** | **Age** | **Sex** | **Tissue** | **Collection_date** | **Store_cond** |
| --- | --- | --- | --- | --- | --- | --- | --- |
| Dairy_SN1 | blood | Saanen goat | 3.5 | female | blood | 2008 | -80 |
| Dairy_SN2 | blood | Saanen goat | 2 | female | blood | 2008 | -80 |
| Dairy_SN3 | blood | Saanen goat | 4 | female | blood | 2008 | -80 |
| Dairy_SN4 | blood | Saanen goat | 3 | female | blood | 2008 | -80 |
| Dairy_SN5 | blood | Saanen goat | 1.5 | female | blood | 2008 | -80 |
| Cashmere_LN1 | blood | Liaoning cashmere goat | 2.2 | female | blood | 2006 | -80 |
| Cashmere_LN2 | blood | Liaoning cashmere goat | 1.5 | female | blood | 2006 | -80 |
| Cashmere_LN3 | blood | Liaoning cashmere goat | 2 | female | blood | 2006 | -80 |
| Cashmere_LN4 | blood | Liaoning cashmere goat | 3 | female | blood | 2006 | -80 |
| Cashmere_LN5 | blood | Liaoning cashmere goat | 2.5 | female | blood | 2006 | -80 |
| Meat_LZ1 | blood | Leizhou goat | 3.5 | female | blood | 2007 | -80 |
| Meat_LZ2 | blood | Leizhou goat | 4 | female | blood | 2007 | -80 |
| Meat_LZ3 | blood | Leizhou goat | 1 | female | blood | 2007 | -80 |
| Meat_LZ4 | blood | Leizhou goat | 2.5 | female | blood | 2007 | -80 |
| Meat_LZ5 | blood | Leizhou goat | 1.5 | female | blood | 2007 | -80 |
| Markhor1 | skin biopsy | wild goat | 2 | male | skin biopsy | 2012 | -80 |
| Markhor2 | skin biopsy | wild goat | 2.5 | male | skin biopsy | 2012 | -80 |
| Sindh ibex1 | skin biopsy | wild goat | 3 | male | skin biopsy | 2013 | -80 |
| Sindh ibex2 | skin biopsy | wild goat | 1.5 | male | skin biopsy | 2013 | -80 |

## 4.2 Sequencing and analysis

### 4.2.1 Sequencing

DNA concentrations were measured by NanoDrop 2000 (Thermo Fisher Scientific), and were sheared with Covaris S220 Sonicator (Covaris) to target of 300-400 bp average size. Fragmented DNA was purified using Sample Purification Beads (Illumina). Adapter-ligated libraries were prepared with the TruSeq Nano DNA Sample Prep Kits (Illumina) according to Illumina-provided protocol. DNA concentrations of the resulting sequencing libraries were measured with the Qubit 2.0 fluorometer dsDNA HS Assay (Thermo Fisher Scientific). Quantities and sizes of the resulting sequencing libraries were analyzed using Agilent BioAnalyzer 2100 (Agilent).  The 500 bp insert size libraries were used in cluster formation on an Illumina cBOT cluster generation system with HiSeq X HD PE Cluster Kits (Illumina). Paired-end sequencing is performed using an Illumina HiSeq X10 following Illumina-provided protocols for 2×150 paired-end sequencing.

### 4.2.2 Read mapping

Before alignment to the reference, we filtered the raw sequencing reads by removing low-quality reads which meets one or more of the following criterias: 1) N-content more than 10%; 2) >60% read length below Q7; 3) reads overlapping >10 bp with the adapter sequence and a maximum of 2 bp mismatches to the adaptor sequence; 4) paired-end reads overlapped by >10 bp with others; 5) duplicated reads. We also trimmed up to 10 bp at the 5’-end or 30 bp at the 3’-end of a read if the local N-content is >20%.

We used BWA 0.5.9 to map the clean pair-end reads from each accession onto the reference genome of *Capra hircus* genome V1 (<http://goat.kiz.ac.cn/>)[25]. The command ‘aln -t 4 -e 10’ was used to find the suffix array coordinates of good hits of each read. Then we used the command ‘sample -a 500’ to convert suffix array coordinates into chromosomal coordinates and paired reads. Other parameters were set to the defaults. We filtered the alignments as follow: 1) mapping quality score lower than 20; 2) non-unique alignments; and 3) duplicated alignments.

### 4.2.3 Sequence variation

First, ANGSD was used to call SNP in population scale using with the parameters referring to a previous publication [26]. We filtered out each locus with minimum depth <8 in all individuals, and call a heterozygous SNP in one individual only when both allele are supported by at least 4 reads. Second, Dindel v1.01 was used to call short indels (1-5 bp) in each individual [27]. We first call an indel only when the non-ref allele is covered by at least 2 read on each strand. Then we filtered out the results which met one or more of the following criteria：1) Quality reported by Dindel below 20; 2) reference homopolymer length longer than 10 bp; 3) length of insertion or deletion longer than 5 bp. Third, software Control-FREEC was used to detect copy number variation (CNV) based on pair-wise comparison [28]. With 1-kb window, we compared the coverage depth between the window and the average depth and identified CNV regions different from the reference. We merged the overlapped CNV regions among different samples. Taken together, a total of 23,924,294 SNPs, 1,899,827 indels (with a size range of 1-5 bp) and 2,317 CNV were identified (Table 1).

## 4.3 Experimental validation of SNP calling

Stringent filtering criteria (see SNP calling) were used and identified 23,924,294 SNPs in the combined domestic and wild goat data. To validate the calling accuracy rate, we designed an independent assay that targets 5 samples, each with 160 loci, a total of 800 SNP loci. The assay uses a capture-NGS-based genotyping method (Genesky Biotechnologies, Shanghai, China). We obtained reliable genotyping calls for 740 loci and a validation rate of 97.43% (721/740).

## 4.4 Phylogenetic analysis

We performed principle component analysis (PCA) with all population-scale autosomal SNPs using the package EIGENSOFT [29]. The phylogenetic tree was constructed based on all autosomal SNPs, with the evolutionary distances measured by p-distance with PHYLIP [30].

## 4.5 SNP desert and SNP-desert-associated genes (SAGs)

Based on the SNP data, we computed SNP rate in different sliding windows, ranging from 2 to 20 kb. We normalized the SNP rates over the length of ≥8 depth aligned sequence (same with the cutoff depth in SNP calling) in each bin rather than the bin size, and bins with less than 1 kb of aligned sequence were rejected.

We found that the distribution of SNP rate shared a similar pattern in different sliding window. Both the mean and medium SNP rate of the dairy breed and the cashmere breed are larger than the meat breed. There is a slight bimodality in the SNP rate distribution with 10 kb window of the meat breed and the cashmere breed (Supplementary Figure 13). A similar effect had been previously reported[31].

We used a 10 kb bin size for our following analyses. Based on the SNP rate in 10 kb sliding window, we picked out the windows with lowest 10% SNP rate of the genome data. SNP rate of these picked windows is lower than 1.95 SNP/kb in the dairy breed, 2.21 SNP/kb in the cashmere breed and 1.26 SNP/kb in the meat breed. Then we join up these windows as longer region if the gap between them ≤10 kb. We defined these low SNP-rate windows or region as “SNP desert”. Many of these SNP deserts are much larger than 20 kb, and some are up to a megabase in size. We identified a total of 277.39 Mb regions as SNP deserts in the dairy breed, 278.33 Mb in the cashmere breed, and 273 Mb in the meat breed. Gene with >30% region residing in a SNP desert, is defined as SNP-desert-associated genes (SAGs). We identified 3,950 SAGs in the dairy breed, 4,395 SAGs in the cashmere breed, and 3,447 SAGs in the meat breed (Supplementary Table 6).

## 4.6 Domestication-associated genes or DAGs

To detect genes under positive selection (regions under domestication sweeps) during the domesticate history; we first combined the three domestic breeds as a single domestic gene pool. Then, using all the 23,924,294 autosomal SNP that presented in domestic and wild population, we calculate the population differentiation level (Fst) between domestic goats and wild goats, and the pooled heterozygosity (Hp) in domestic goats.

For Hp analysis, using a 100 kb sliding window, we first determined the number of reads corresponding to the most and least abundant SNP alleles (nMAJ and nMIN), Hp=2∑nMAJ∑nMIN/(∑nMAJ+∑nMIN)^2^, transformed Hp into ZHp: ZHp= (Hp-μHp)/ σHp. We applied a threshold of ZHp = -3, 81 windows resided as outlies (ZHp<-3). With the same 100 kb window, we calculated the fixation index (Fst) between 15 domestic and 4 wild goats. We then transformed Fst into ZFst: ZFst= (Fst-μFst)/ σFst. We applied a threshold of ZFst > 3 for detecting high detergency regions, 250 windows were detected. In the 81 ZHp < -3 windows, 32 (39.5%) have a high divergence between wild and domestic goats (ZFst > 3). 67 genes residing in these 32 extracted regions were indicated as domestication-association genes (DAGs).

## 4.7 Trait-associated genes (TAGs)

For each breed, we compared the levels of polymorphism and divergence between this breed and other breeds (Fst and XP-EHH) to identify trait-associated genes (TAGs) for this breed. To identify the candidate selective genes for each trait, we measured the pairwise Fst and HKA test between one domestic breed and pool of other two breeds. The windows pass the threshold of ZFst = 4 and top 1% XP-EHH scores were extracted as candidate selective sweep regions. Genes residing in these extracted regions were indicated as candidate selective genes.

We computed a measure of population genetic differentiation, Fst, between the dairy breed and others. The rationale for this test is that selective driven differentiation can be captured from differences in allele frequencies between the dairy and other breeds. Outliers in the Fst distribution indicate positive selection. We computed Fst for all windows as described before.

### 4.7.1 Cross-population extended haplotype homozygosity (XP-EHH) scan

We estimated the cross-population extended haplotype homozygosity (XP-EHH) with software selscan (v1.2.0). The threshold for identifying candidate genes was set to the top 1% percentile outliers.

### 4.7.2 QTL mapping

We downloaded known sheep and cow QTL data from Animal QTLdb and qualified the data by filtering out “trait association” with p>0.05, aligned the genome sequences of sheep and goat with lastz (version 1.02.00), based on the axt file produced by lastz, and mapped the sheep QTL to goat chromosomes.

## 4.8 Signatures of artificial selection Validation Samples

To validate the sequence variations at population level, we genotyped by sequencing PCR amplified fragments from 7 domestic breeds, including 99 Saanen, 85 Liaoning cashmere, 23 Leizhou, 16 Dera Din Panah (DDP), 30 Guanzhong, 26 Inner Mongolian cashmere, and 24 Hainan goats. The samples of Markhor and Sind ibex were collected from skin biopsies, Quetta, Pakistan. Blood samples of DDP breeds were collected in Pakistan, and others were collected in China.

## 4.9 Copy number variations and association analysis

Copy number variation was genotyped by using the AccuCopy assay, which is a CNV genotyping method based on multiplex competitive amplification by Genesky Biotechnologies (Shanghai, China). The basic molecular principle of AccuCopy has been described elsewhere as a reliable and efficient tool for CNV genotyping [32]. We used this technology for quantitative analysis of copy numbers of 12 genes for the DNA samples from 130 dairy goats. In total, twelve multiplex PCR panels were designed to amplify 12 target segments.

# 5. REFERENCES

1. Kutukculer N, Gulez N, Karaca NE, Aksu G, Berdeli A: **Three different classifications, B lymphocyte subpopulations, TNFRSF13B (TACI), TNFRSF13C (BAFF-R), TNFSF13 (APRIL) gene mutations, CTLA-4 and ICOS gene polymorphisms in Turkish patients with common variable immunodeficiency,** *J Clin Immunol*, vol. **32**, no.7, pp**.** 1165-1179, 2012.

2. Verardo LL, Nascimento CS, Silva FF, Gasparino E, Martins MF, Toriyama E, Faria VR, Botelho ME, Costa KA, Lopes PS, Guimaraes SE: **Identification and validation of differentially expressed genes from pig skeletal muscle,** *J Anim Breed Genet*, vol. **130**, no.7, pp**.** 372-381, 2013.

3. Ito M: **Function and molecular evolution of mammalian Sox15, a singleton in the SoxG group of transcription factors,** *Int J Biochem Cell Biol*, vol. **42**, no.7, pp**.** 449-452, 2010.

4. Bontekoe CJ, McIlwain KL, Nieuwenhuizen IM, Yuva-Paylor LA, Nellis A, Willemsen R, Fang Z, Kirkpatrick L, Bakker CE, McAninch R, et al: **Knockout mouse model for Fxr2: a model for mental retardation,** *Hum Mol Genet*, vol. **11**, no.7, pp**.** 487-498, 2002.

5. Li M, Tian S, Jin L, Zhou G, Li Y, Zhang Y, Wang T, Yeung CK, Chen L, Ma J, et al: **Genomic analyses identify distinct patterns of selection in domesticated pigs and Tibetan wild boars,** *Nat Genet*, vol. **45**, no.7, pp**.** 1431-1438, 2013.

6. Mikawa S, Morozumi T, Shimanuki S, Hayashi T, Uenishi H, Domukai M, Okumura N, Awata T: **Fine mapping of a swine quantitative trait locus for number of vertebrae and analysis of an orphan nuclear receptor, germ cell nuclear factor (NR6A1),** *Genome Res*, vol. **17**, no.7, pp**.** 586-593, 2007.

7. Carneiro M, Rubin CJ, Di Palma F, Albert FW, Alfoldi J, Barrio AM, Pielberg G, Rafati N, Sayyab S, Turner-Maier J, et al: **Rabbit genome analysis reveals a polygenic basis for phenotypic change during domestication,** *Science*, vol. **345**, no.7, pp**.** 1074-1079, 2014.

8. Rincon G, Farber EA, Farber CR, Nkrumah JD, Medrano JF: **Polymorphisms in the STAT6 gene and their association with carcass traits in feedlot cattle,** *Anim Genet*, vol. **40**, no.7, pp**.** 878-882, 2009.

9. Desvignes L, Weidinger C, Shaw P, Vaeth M, Ribierre T, Liu M, Fergus T, Kozhaya L, McVoy L, Unutmaz D, et al: **STIM1 controls T cell-mediated immune regulation and inflammation in chronic infection,** *J Clin Invest*, vol. **125**, no.7, pp**.** 2347-2362, 2015.

10. Fujimoto S, Negishi M, Katoh H: **RhoG promotes neural progenitor cell proliferation in mouse cerebral cortex,** *Mol Biol Cell*, vol. **20**, no.7, pp**.** 4941-4950, 2009.

11. Leon G, Sanchez-Ruiloba L, Perez-Rodriguez A, Gragera T, Martinez N, Hernandez S, Anta B, Calero O, Garcia-Dominguez CA, Dura LM, et al: **Shoc2/Sur8 protein regulates neurite outgrowth,** *PLoS One*, vol. **9**, no.7, pp**.** e114837, 2014.

12. Liu Q, Zhang J, Zerbinatti C, Zhan Y, Kolber BJ, Herz J, Muglia LJ, Bu G: **Lipoprotein receptor LRP1 regulates leptin signaling and energy homeostasis in the adult central nervous system,** *PLoS Biol*, vol. **9**, no.7, pp**.** e1000575, 2011.

13. Maestu J, Allik J, Merenakk L, Eensoo D, Parik J, Veidebaum T, Harro J: **Associations between an alpha 2A adrenergic receptor gene polymorphism and adolescent personality,** *Am J Med Genet B Neuropsychiatr Genet*, vol. **147B**, no.7, pp**.** 418-423, 2008.

14. Wang X, Maltecca C, Tal-Stein R, Lipkin E, Khatib H: **Association of bovine fibroblast growth factor 2 (FGF2) gene with milk fat and productive life: an example of the ability of the candidate pathway strategy to identify quantitative trait genes,** *J Dairy Sci*, vol. **91**, no.7, pp**.** 2475-2480, 2008.

15. Iglesias-Platas I, Court F, Camprubi C, Sparago A, Guillaumet-Adkins A, Martin-Trujillo A, Riccio A, Moore GE, Monk D: **Imprinting at the PLAGL1 domain is contained within a 70-kb CTCF/cohesin-mediated non-allelic chromatin loop,** *Nucleic Acids Res*, vol. **41**, no.7, pp**.** 2171-2179, 2013.

16. Latour S, Aguilar C: **XIAP deficiency syndrome in humans,** *Semin Cell Dev Biol*, vol. **39**, no.7, pp**.** 115-123, 2015.

17. Leroy C, Jacquemont ML, Doray B, Lamblin D, Cormier-Daire V, Philippe A, Nusbaum S, Patrat C, Steffann J, Colleaux L, et al: **Xq25 duplication: the crucial role of the STAG2 gene in this novel human cohesinopathy,** *Clin Genet*, vol., no.7, pp**.**, 2015.

18. Kim JH, Jung SH, Bae JS, Lee HS, Yim SH, Park SY, Bang SY, Hu HJ, Shin HD, Bae SC, Chung YJ: **Deletion variants of RABGAP1L, 10q21.3, and C4 are associated with the risk of systemic lupus erythematosus in Korean women,** *Arthritis Rheum*, vol. **65**, no.7, pp**.** 1055-1063, 2013.

19. Tang RQ, Zhao XZ, Shi YY, Tang W, Gu NF, Feng GY, Xing YL, Zhu SM, Sang H, Liang PJ, He L: **Family-based association study of Epsin 4 and Schizophrenia,** *Mol Psychiatry*, vol. **11**, no.7, pp**.** 395-399, 2006.

20. Zhang J, Fang Z, Jud C, Vansteensel MJ, Kaasik K, Lee CC, Albrecht U, Tamanini F, Meijer JH, Oostra BA, Nelson DL: **Fragile X-related proteins regulate mammalian circadian behavioral rhythms,** *Am J Hum Genet*, vol. **83**, no.7, pp**.** 43-52, 2008.

21. Dillon SR, Gross JA, Ansell SM, Novak AJ: **An APRIL to remember: novel TNF ligands as therapeutic targets,** *Nat Rev Drug Discov*, vol. **5**, no.7, pp**.** 235-246, 2006.

22. Rubin CJ, Megens HJ, Martinez Barrio A, Maqbool K, Sayyab S, Schwochow D, Wang C, Carlborg O, Jern P, Jorgensen CB, et al: **Strong signatures of selection in the domestic pig genome,** *Proc Natl Acad Sci U S A*, vol. **109**, no.7, pp**.** 19529-19536, 2012.

23. Erickson CA, Posey DJ, Stigler KA, Mullett J, Katschke AR, McDougle CJ: **A retrospective study of memantine in children and adolescents with pervasive developmental disorders,** *Psychopharmacology (Berl)*, vol. **191**, no.7, pp**.** 141-147, 2007.

24. Xu M, Qin Y, Qu J, Lu C, Wang Y, Wu W, Song L, Wang S, Chen F, Shen H, et al: **Evaluation of five candidate genes from GWAS for association with oligozoospermia in a Han Chinese population,** *PLoS One*, vol. **8**, no.7, pp**.** e80374, 2013.

25. Li H, Durbin R: **Fast and accurate long-read alignment with Burrows-Wheeler transform,** *Bioinformatics*, vol. **26**, no.7, pp**.** 589-595, 2010.

26. Liu S, Lorenzen ED, Fumagalli M, Li B, Harris K, Xiong Z, Zhou L, Korneliussen TS, Somel M, Babbitt C, et al: **Population genomics reveal recent speciation and rapid evolutionary adaptation in polar bears,** *Cell*, vol. **157**, no.7, pp**.** 785-794, 2014.

27. Albers CA, Lunter G, MacArthur DG, McVean G, Ouwehand WH, Durbin R: **Dindel: accurate indel calls from short-read data,** *Genome Res*, vol. **21**, no.7, pp**.** 961-973, 2011.

28. Boeva V, Popova T, Bleakley K, Chiche P, Cappo J, Schleiermacher G, Janoueix-Lerosey I, Delattre O, Barillot E: **Control-FREEC: a tool for assessing copy number and allelic content using next-generation sequencing data,** *Bioinformatics*, vol. **28**, no.7, pp**.** 423-425, 2012.

29. Patterson N, Price AL, Reich D: **Population structure and eigenanalysis,** *PLoS Genet*, vol. **2**, no.7, pp**.** e190, 2006.

30. Retief JD: **Phylogenetic analysis using PHYLIP,** *Methods Mol Biol*, vol. **132**, no.7, pp**.** 243-258, 2000.

31. Wang L, Hao L, Li X, Hu S, Ge S, Yu J: **SNP deserts of Asian cultivated rice: genomic regions under domestication,** *J Evol Biol*, vol. **22**, no.7, pp**.** 751-761, 2009.

32. Wu X, Lu Y, Ding Q, You G, Dai J, Xi X, Wang H, Wang X: **Characterisation of large F9 deletions in seven unrelated patients with severe haemophilia B,** *Thromb Haemost*, vol. **112**, no.7, pp**.** 459-465, 2014.
