# Supplementary material for: Genome-wide definition of selective sweeps reveals molecular evidence of trait-driven domestication among elite goat (Capra species) breeds for the production of dairy, cashmere, and meat
Source: Gigascience. 2018 Aug 27;7(12):giy105. doi: 10.1093/gigascience/giy105 (PMC6287099; doi:10.1093/gigascience/giy105)
Supplement: Response_to_Reviewer_Comments(Original_Submission).pdf [file giy105_response_to_reviewer_comments(original_submission).pdf]

Author's Response To Reviewer Comments

Close

Dear Dr. Hans Zauner
Thank you very much for handling our manuscript "Genome-wide definition of selective sweeps reveals molecular evidence of trait-driven domestication among elite goat (Capra species) breeds for the production of dairy, cashmere, and meat" (GIGA-D-17-00226). We appreciate all comments from the reviewers, which are extremely helpful for us to improve our manuscript. We have now revised our manuscript accordingly as highlighted in colored and quotation-marked. Because of additional figures, we attach a list of current figures and tables, which are referenced to the last version so that the revision can be readily tracked.
We list all responses as follows:
From editor:
1. The section has been extensively rewritten.
2. The language has been professionally edited by an English editing service agency, Springer Nature Language Editing.

****
From REVIEWERS
****
Reviewer #1:

In this manuscript the authors have identified several polymorphisms in domestic goat breeds that could be linked to their domestication process.
The authors have also shown that these polymorphisms could be good molecular markers for genetic mapping and breeding.
These findings are interesting and the authors have presented some novel data. However, there are some major issues that need to be addressed. In addition, in its present state the overall report is difficult to follow for the English reader and requires editing.

Response: Thanks for your positive comments and encouragement. We have substantially revised the manuscript, and the manuscript has been professionally edited by an English editing service agency, Springer Nature Language Editing.

* Are the conclusions adequately supported by the data shown? No
- Poor discussion. It does not allow to link the results found with the conclusions about the biological meaning. I suggest the authors to develop more the discussion part, to make clearer to the reader the impact of the findings.
- Poor conclusions

Response: We have substantially revised the manuscript to emphasize major points.

* Does the manuscript adhere to the journal's guidelines on minimum standards of reporting? No
- Organisms: report source, species, strain, sex, age, husbandry, inbred and strain characteristics of transgenic and mutant animals --> Lack of information in the materials and methods section

Response: We add the information (source, species, sex) in the Materials and Methods section (Line 329-331). And some more details are added as supplementary methods 4.1.

- Availability of Data and Materials --> Have the SNPs found (T217C for instance) being submitted to the genebank dbSNP and publicly available? If so, please add the reference

Response: We did not find goat SNP collection at Genebank. We submitted our goat SNP data to EBI. Due to the journal policy, we will release the accession numbers as the genome sequence data release at NCBI.

Comments:

- Line 37 "Surprisingly": I would just expect that, isn't it?. A breed selected for production traits, would have less SNP content in the genes of interest for which have been selected.

Response: We also think there should be less SNP content in the gene of interest for which have been selected in the trait-selected breeds. We deleted the word “Surprisingly” in line37.

- Lines 112-113: "...indicating recent introduction of genetic heterogeneity...": Do you have data sustaining this assumption? References?

Response: The Saanen breed is a famous dairy breed from Switzerland. It has been introduced to many parts of the world, giving rise to numerous local hybrids. For instance, it was brought to China in 1920s.

[1] Jean-Paul Dubeuf. An international panorama of goat selection and breeds. Livestock Science, 2009, 120(3): 225-231.
[2] Breeds of domestic animal and poultry in China, sheep and goat breeds in China writing group, Sheep and goat breeds in China.1988. (In Chinese).

- Lines 111-114 "the dairy breed appears to have slightly more unique SNPs," and Supplementary figure 7: In this figure in fact, dairy breed and cashmere breed seem to have similar number of SNP, however, meat breed shows a really lower number of SNPs compared to the other two. What do you think that could be due to?

Response: We are thinking of two obvious factors. First, as we show in Figure 1, the 5 representative individuals of the meat breed exhibit closer relationship that leaves less within-breed polymorphisms as compared to the other two breeds. Second, the Leizhou breed itself is also known to be heavily inbreeded in its recent history, and such practice may lead to an effect of purifying selections.

- A high number of polymorphisms shared between dairy and cashmere in general. Cashmere breeds in fact, usually have a dual-purpose: dairy and Is it the case of this breed? Does the LN cashmere breed come from dairy breeds? Does by chance could come from any of the dairy breeds included in this work? That will explain these results and similarities.

Response: Liaoning Cashmere is a famous cashmere breed in China. Its within-breed trait selection began about 1980s. Before this selection for cashmere, it might be actually raised

for two purpose, cashmere and milk.

[1] Suggestion for breed selection of Liaoning cashmere goat. Zhao QT, Modern Journal of Animal Husbandry and Veterinary Medicine 1981, 21-23. (In Chinese).

- Paragraph from lines 161-170: It should be rewritten, I don't think what it is explained is clear.

Response: (lines 164-172), We used principal component analysis (PCA) and phylogeny reconstruction to evaluate the population structure of the domestic breeds. First, based on our genome-wide SNP data, we found that Sindh ibex is genetically closer to the domestic breeds as compared to Markhor, consistent with previous reports. Second, our PCA result suggests that the domestic breeds and the wild breeds are both distant and distinct (Fig. 1a), where, as the neighbor-joining tree shows, the two Chinese domestic breeds are closer to each other and the Saanen breed is closer to the wild goats than the other two domestic breeds. Third, all results collectively suggest that the domestication traits for dairy production may occur ahead of cashmere and meat in goat domestication (Fig. 1).

- Figure 1B "the Saanen breed is rather close to the wild goats" Not clear in the figure, maybe trying to make a zoom of this part of the branch?

Response: Both Figure1a (component 2) and 1b suggest this point although in 1b the distance is very subtle.

- Lines 168-170 "all results suggest that the domestication traits of dairy production may occur ahead of cashmere and meat in the domestication history of goats": There are already references suggesting the fact that goats could primarily have be domesticated for dairy purposes, so you should include them.

Response: Yes. We added the following reference to the manuscript (Line 172).

[1] Jean-Paul Dubeuf. An international panorama of goat selection and breeds. Livestock Science, 2009, 120(3): 225-231.

- Section TAGs in the dairy breed: Various TAGs, however, the authors does not explain how and why this could be due to. It needs more concretion.

Response: It is true that the dairy breeds have more TAGs due to more SNP desert but we do not have idea why but suggest that it is historic on one hand and function-related on the other. We did not speculate much as the dataset is not big enough for further scrutiny.

- T217C SNP: Lines 268-272: The SNP seems to only exist in domestic goats, what about in wild goats? Why do you think the explanation of this SNP is due to the largest body of dairy goats when it is in this breed where the most frequent allele is T as in concordance with the rest of the species analyzed? Shouldn't you look for an explanation in the meat breeds where the presence of C is dominant?

Response: We revised the manuscript and attempt to explain the points.
In Figure 5, we compared the difference of the GDF5 T217C locus in the two meat breeds

(Leizhou and Hainan) and the 4 wild goats, where the C allele is dominant in the domestic meat breeds and the T allele is dominant in the wild goats. Since the T allele is also seen in the dairy goats, we suggest that T is ancestral where as C is selected. In addition, we have looked into the breeding history of Leizhou and learnt that the body size has been smaller than Sannen and Liaoning cashmere goats. GDF5 is a member of the TGF-beta superfamily, which is involved in height [1] and multiple skeletal structures [2].

[1] S Sanna, Jackson, A U, Nagaraja, R, et al. Common variants in the GDF5-UQCC region are associated with variation in human height. Nature genetics 2008; 40: 198-203.
[2] Settle SH Jr, Rountree RB, Sinha A, Thacker A, Higgins K, Kingsley DM. Multiple joint and skeletal patterning defects caused by single and double mutations in the mouse Gdf6 and Gdf5 genes. Dev Biol. 2003 Feb 1; 254(1):116-30.

- QTL mapping: What about taking QTL data also from cows? Wouldn't it be more informative?

Response: We used the cattle information that includes cow data. And there has not been independent QTL dataset for cow.

- I find the presence of SNP deserts really interesting. But I find a lack of further explanation. Why do you think there is a SNP desert along the RSRC1 sequence (Fig.2)? What could this gene have as special? Could you take a look to the wild species or to other species to compare this phenomenon?

Response: For defining SNP desert, we randomly choose one gene (RSRC1) as an example. There are a lot of SNP desert genes in our dataset (Supplementary tables 11, 12, 13). We added data from wild goat, human, and cheetah to illustrate this phenomenon. it is clear that the domestic goat breeds have lower SNP rates in the RSRC1 gene locus when compared to the wild goat and other species (Supplementary Figure 16).

Figures:
- Figure 1a: Please add the % of the variability explained in the PCA graph.
- Figure 1b: For me was not that clear to see in the neighbor tree the branch were dairy and wild breeds are together, maybe a zoom of the figure?

Response: We added the percentage of SNPs that contribute to the distribution in PCA graph.

English correction is needed. Some examples:
- Line 38: "breeds are classified into Gene Ontology categories"
- Line 40: " trait-specificity. Whereas..."
- Line 50-51: "crucial factors were selected including docility.."
- Line 58: "dramatically around 200 years ago..."
- Line 58-59: "Selection increased intensively.."
- Line 60: " among breed was seriously reduced"
- Line 76: " it is still lagging..."
- Sentence in lines 99-100 "In addition, validated our SNP..". It needs to be re-written
- Sentence in lines 211-215. It needs to be re-written
- Sentence in lines 315-317 "To validate them in a larger population..." It needs to be re-written, I don't understand the sentence

Response: We have revised the manuscript extensively, and the manuscript has been professionally edited by an English editing service agency (Springer Nature Language Editing).

Others:
- Line 77: " cattle , pig , dog , and chicken ." Remove space between words and punctuations/grammatical marks.

Response: We extensively proofread the entire manuscript accordingly.

Reviewer #2: Main: line 388: HKA is a method to detect signatures of selection for macroevolution, therefore related to selection between species (Vitti et al., 2013). Therefore, it is not suited to compare populations within a species, but you can apply it to the previous comparison (wild vs domestic). In its place, I would suggest the application of methods such as LKT (Lewontin-Krakauer Test), FLK, XP-EHH or XP-CLR, which have results comparable with Fst.

Response: We have re-analyzed this part with XP-EHH and revised the manuscript accordingly (lines 236-239 and lines 394-396).

lines 336-341: What is the reads length produced from the sequencer?

Response: The average raw reads length is 150 bp. We have added this information in Methods (line 340).

line 343: Why use such an old version of BWA (v 0.5.9, released in 2011) when the more "modern" 0.7.12 is available since 2014?

Response: We acquired the raw data in 2012, so BWA 0.5.9 was used. We tested the alignment of 5 samples using BWA 0.7.12, and the genotype calls between the two versions are 99% identical. We have also randomly selected 160 SNPs and validated them with capture-NGS-based genotyping method (Genesky Biotechnologies, Shanghai, China), and the accuracy is 97.43%.

line 344: Why use the aln alignment algorithm instead of mem?

Response: BWA-MEM is faster and more accurate in long-read alignment. For Illumina sequence reads, there are high error rate at the ends of sequence reads. BWA-backtrack algorithm (aln/sampe) trims low-quality bases from the 3'-end before alignment. Our data is generated with the Illumina platform, so we choose BWA-backtrack algorithm (aln/sampe) for sequence quality control.

[1] http://bio-bwa.sourceforge.net/
[2] Li H. and Durbin R. (2009) Fast and accurate short read alignment with Burrows-Wheeler Transform. Bioinformatics, 25:1754-60.

lines 357-358: You call a SNP if you have at least 4 reads per allele, but only 2 reads for indels: why this difference?

Response: SNP calls correlate to the sequence error rate in the Illumina platform, where the quality of base calling decreases when the accuracy of SNP calling is lower. The correlation between indel quality and base calling errors is relatively poorer. In other words, miscalled indels are rarer than SNPs.

lines 366-370: please add references for PCA (EIGENSOFT), FRAPPE and PHYLIP software.

Response: We added two references in Methods (line375 and line 377). In this version, we eliminated FRAPPE.

[1] PCA (EIGENSOFT) N Patterson, Price, A L, and Reich, D. Population structure and eigenanalysis. PLoS Genet 2006; 2: e190.
[2] PHYLIP J D Retief. Phylogenetic analysis using PHYLIP. Methods Mol Biol 2000; 132: 243-58.

lines 371-377: does the SNP deserts regions considers the indels? If not, why did you excluded them? in addition, it could be interesting to compare the SNP deserts regions with some well-established method to detect low diversity regions (e.g. ROH)

Response: We did not include indels when screening SNP deserts. The number of SNPs is 10 times more than indels so they have little effect on SNP deserts. ROH method is based on allele frequencies. In our small population, the distribution of allele frequency may differ when a large population is used. Therefore, we use the number of SNPs in a window rather than frequency itself.

line 379: Is Hp the pooled heterozygosity cited by Bahbahani et al. (2017; DOI: 10.3389/fgene.2017.00068)? If so, it should be cited properly, referring to some works that compare different species, and defining what kind of selection it identifies.

Response: Hp, pooled heterozygosity. We added a reference (Result line 387). Hp is used to detect putative selective sweeps during domestication(DAG).

[1] CJ Rubin, Zody MC, Eriksson J, Meadows JR, Sherwood E, Webster MT, Jiang L, Ingman M, Sharpe T, Ka S, et al. "Whole-genome resequencing reveals loci under selection during chicken domestication," Nature, vol. 464, no.7, pp. 587-591, 2010.

Close
