# Supplementary material for: Genome-wide definition of selective sweeps reveals molecular evidence of trait-driven domestication among elite goat (Capra species) breeds for the production of dairy, cashmere, and meat
Source: Gigascience. 2018 Aug 27;7(12):giy105. doi: 10.1093/gigascience/giy105 (PMC6287099; doi:10.1093/gigascience/giy105)
Supplement: Reviewer_1_Report_(Original_Submission) -- Judit Salces Ortiz — 10/24/2017 Reviewed [file giy105_reviewer_1_report_(original_submission).pdf]

Reviewer Report

Title: Genome-wide definition of selective sweeps reveals molecular evidence of trait-driven domestication among elite goat (Capra species) breeds for the production of dairy, cashmere, and meat

Version: Original Submission **Date:** 10/24/2017

Reviewer name: Judit Salces Ortiz

Reviewer Comments to Author:

In this manuscript the authors have identified several polymorphisms in domestic goat breeds that could be linked to their domestication process.
The authors have also shown that these polymorphisms could be good molecular markers for genetic mapping and breeding.
These findings are interesting and the authors have presented some novel data. However, there are some major issues that need to be addressed. In addition, in its present state the overall report is difficult to follow for the English reader and requires editing.

* Are the conclusions adequately supported by the data shown? No

- Poor discussion. It does not allow to link the results found with the conclusions about the biological meaning. I suggest the authors to develop more the discussion part, to make clearer to the reader the impact of the findings.
- Poor conclusions

* Does the manuscript adhere to the journal's guidelines on minimum standards of reporting? No

- Organisms: report source, species, strain, sex, age, husbandry, inbred and strain characteristics of transgenic and mutant animals --> Lack of information in the materials and methods section
- Availability of Data and Materials --> Have the SNPs found (T217C for instance) being submitted to the genebank dbSNP and publicly available? If so, please add the reference

Comments:

- Line 37 "Surprisingly": I would just expect that, isn't it?. A breed selected for production traits, would have less SNP content in the genes of interest for which have been selected.

- Lines 112-113: "...indicating recent introduction of genetic heterogeneity...": Do you have data sustaining this assumption? References?

- Lines 111-114 "the dairy breed appears to have slightly more unique SNPs," and Supplementary figure 7: In this figure in fact, dairy breed and cashmere breed seem to have similar number of SNP, however, meat breed shows a really lower number of SNPs compared to the other two. What do you think that could be due to?

- A high number of polymorphisms shared between dairy and cashmere in general. Cashmere breeds in fact, usually have a dual-purpose: dairy and Is it the case of this breed? Does the LN cashmere breed comes from dairy breeds? Does by chance could come from any of the dairy breeds included in this work? That will explain these results and similarities.

- Paragraph from lines 161-170: It should be rewritten, I dont think what it is explained is clear.
- Figure 1B "the Saanen breed is rather close to the wild goats" Not clear in the figure, maybe trying to make a zoom of this part of the branch?

- Lines 168-170 "all results suggest that the domestication traits of dairy production may occur ahead of cashmere and meat in the domestication history of goats": There are already references suggesting the fact that goats could primarily have be domesticated for dairy purposes, so you should include them.

- Section TAGs in the dairy breed: Various TAGs, however, the authors does not explain how and why this could be due to. It needs more concretion

- T217C SNP: Lines 268-272: The SNP seems to only exist in domestic goats, what about in wild goats? Why do you think the explanation of this SNP is due to the largest body of dairy goats when it is in this breed where the most frequent allele is T as in concordance with the rest of the species analyzed? Shouldn't you look for an explanation in the meat breeds where the presence of C is dominant?

- QTL mapping: What about taking QTL data also from cows? Wouldn't it be more informative?

- I find the presence of SNP deserts really interesting. But I find a lack of further explanation. Why do you think there is a SNP desert along the RSRC1 sequence (Fig.2))? What could this gene have as special? Could you take a look to the wild species or to other species to compare this phenomenon?

Figures:

- Figure 1a: Please add the % of the variability explained in the PCA graph.
- Figure 1b: Fr me was not that clear to see in the neighbor tree the branch were dairy and wild breeds are together, maybe a zoom of the figure?

English correction is needed. Some examples:

- Line 38: "breeds are classified into Gene Ontology categories"
- Line 40: " trait-specificity. Whereas..."
- Line 50-51: "crucial factors were selected including docility.."
- Line 58: "dramatically around 200 years ago..."
- Line 58-59: "Selection increased intensively.."
- Line 60: " among breed was seriously reduced"
- Line 76: " it is still lagging..."
- Sentence in lines 99-100 "In addition, validated our SNP..". It needs to be re-written
- Sentence in lines 211-215. It needs to be re-written
- Sentence in lines 315-317 "To validate them in a larger population..." It needs to be re-written, I don't understand the sentence

Others:
- Line 77: " cattle , pig , dog , and chicken ." Remove space between words and punctuations/grammatical marks.

Methods

Are the methods appropriate to the aims of the study, are they well described, and are necessary controls included? Yes

Conclusions

Are the conclusions adequately supported by the data shown? No

Reporting Standards

Does the manuscript adhere to the journal's guidelines on minimum standards of reporting? No

Choose an item.

Statistics

Are you able to assess all statistics in the manuscript, including the appropriateness of statistical tests used? No, and I do not feel adequately qualified to assess the statistics.

Quality of Written English

Please indicate the quality of language in the manuscript: Needs some language corrections before being published

Declaration of Competing Interests

Please complete a declaration of competing interests, considering the following questions:

- Have you in the past five years received reimbursements, fees, funding, or salary from an organisation that may in any way gain or lose financially from the publication of this manuscript, either now or in the future?
- Do you hold any stocks or shares in an organisation that may in any way gain or lose financially from the publication of this manuscript, either now or in the future?
- Do you hold or are you currently applying for any patents relating to the content of the manuscript?
- Have you received reimbursements, fees, funding, or salary from an organization that holds or has applied for patents relating to the content of the manuscript?
- Do you have any other financial competing interests?
- Do you have any non-financial competing interests in relation to this paper?

If you can answer no to all of the above, write 'I declare that I have no competing interests' below. If your reply is yes to any, please give details below.

I declare that I have no competing interests

I agree to the open peer review policy of the journal. I understand that my name will be included on my report to the authors and, if the manuscript is accepted for publication, my named report including any attachments I upload will be posted on the website along with the authors' responses. I agree for my

report to be made available under an Open Access Creative Commons CC-BY license (http://creativecommons.org/licenses/by/4.0/). I understand that any comments which I do not wish to be included in my named report can be included as confidential comments to the editors, which will not be published.

I agree to the open peer review policy of the journal

To further support our reviewers, we have joined with Publons, where you can gain additional credit to further highlight your hard work (see: https://publons.com/journal/530/gigascience). On publication of this paper, your review will be automatically added to Publons, you can then choose whether or not to claim your Publons credit. I understand this statement.

Yes Choose an item.
