# Supplementary material for: Genome-wide definition of selective sweeps reveals molecular evidence of trait-driven domestication among elite goat (Capra species) breeds for the production of dairy, cashmere, and meat
Source: Gigascience. 2018 Aug 27;7(12):giy105. doi: 10.1093/gigascience/giy105 (PMC6287099; doi:10.1093/gigascience/giy105)
Supplement: Reviewer_2_Report_(Original_Submission) -- Alessandra Stella — 11/16/2017 Reviewed [file giy105_reviewer_2_report_(original_submission).pdf]

Reviewer Report

Title: Genome-wide definition of selective sweeps reveals molecular evidence of trait-driven domestication among elite goat (Capra species) breeds for the production of dairy, cashmere, and meat

Version: Original Submission **Date:** 11/16/2017

Reviewer name: Alessandra Stella

Reviewer Comments to Author:

Main: line 388: HKA is a method to detect signatures of selection for macroevolution, therefore related to selection between species (Vitti et al., 2013). Therefore, it is not suited to compare populations within a species, but you can apply it to the previous comparison (wild vs domestic). In its place, I would suggest the application of methods such as LKT (Lewontin-Krakauer Test), FLK, XP-EHH or XP-CLR, which have results comparable with Fst.

lines 336-341: What is the reads length produced from the sequencer?
line 343: Why use such an old version of BWA (v 0.5.9, released in 2011) when the more "modern" 0.7.12 is available since 2014?
line 344: Why use the aln alignment algorithm instead of mem?
lines 357-358: You call a SNP if you have at least 4 reads per allele, but only 2 reads for indels: why this difference?
lines 366-370: please add references for PCA (EIGENSOFT), FRAPPE and PHYLIP software.
lines 371-377: does the SNP deserts regions considers the indels? If not, why did you excluded them? in addition, it could be interesting to compare the SNP deserts regions with some well-established method to detect low diversity regions (e.g. ROH)
line 379: Is Hp the pooled heterozygosity cited by Bahbahani et al. (2017; DOI: 10.3389/fgene.2017.00068)? If so, it should be cited properly, referring to some works that compare different species, and defining what kind of selection it identifies.

Methods

Are the methods appropriate to the aims of the study, are they well described, and are necessary controls included? No

Conclusions

Are the conclusions adequately supported by the data shown? Yes

Reporting Standards

Does the manuscript adhere to the journal's guidelines on minimum standards of reporting? Yes

Choose an item.

Statistics

Are you able to assess all statistics in the manuscript, including the appropriateness of statistical tests used? Yes, and I have assessed the statistics in my report.

Quality of Written English

Please indicate the quality of language in the manuscript: Acceptable

Declaration of Competing Interests

Please complete a declaration of competing interests, considering the following questions:

- Have you in the past five years received reimbursements, fees, funding, or salary from an organisation that may in any way gain or lose financially from the publication of this manuscript, either now or in the future?
- Do you hold any stocks or shares in an organisation that may in any way gain or lose financially from the publication of this manuscript, either now or in the future?
- Do you hold or are you currently applying for any patents relating to the content of the manuscript?
- Have you received reimbursements, fees, funding, or salary from an organization that holds or has applied for patents relating to the content of the manuscript?
- Do you have any other financial competing interests?
- Do you have any non-financial competing interests in relation to this paper?

If you can answer no to all of the above, write 'I declare that I have no competing interests' below. If your reply is yes to any, please give details below.

I declare that I have no competing interests

I agree to the open peer review policy of the journal. I understand that my name will be included on my report to the authors and, if the manuscript is accepted for publication, my named report including any attachments I upload will be posted on the website along with the authors' responses. I agree for my report to be made available under an Open Access Creative Commons CC-BY license (http://creativecommons.org/licenses/by/4.0/). I understand that any comments which I do not wish to be included in my named report can be included as confidential comments to the editors, which will not be published.

I agree to the open peer review policy of the journal

To further support our reviewers, we have joined with Publons, where you can gain additional credit to further highlight your hard work (see: https://publons.com/journal/530/gigascience). On publication of this paper, your review will be automatically added to Publons, you can then choose whether or not to claim your Publons credit. I understand this statement.

Yes Choose an item.
